# Impacts of systemic milieu on cerebrovascular and brain aging: insights from heterochronic parabiosis, blood exchange, and plasma transfer experiments

**DOI:** 10.1007/s11357-025-01657-y

**Published:** 2025-05-23

**Authors:** Rafal Gulej, Roland Patai, Anna Ungvari, Attila Kallai, Stefano Tarantini, Andriy Yabluchanskiy, Derek M. Huffman, Michael J. Conboy, Irina M. Conboy, Mika Kivimäki, Anna Csiszar, Zoltan Ungvari

**Affiliations:** 1 Vascular Cognitive Impairment, Neurodegeneration, and Healthy Brain Aging Program, Department of Neurosurgery, University of Oklahoma Health Sciences Center, Oklahoma City, OK USA; 2 Oklahoma Center for GeroScience and Healthy Brain Aging, University of Oklahoma Health Sciences Center, Oklahoma City, OK USA; 3 International Training Program in Geroscience, Doctoral College/Institute of Preventive Medicine and Public Health, Semmelweis University, Budapest, Hungary; 4 Institute of Preventive Medicine and Public Health, Semmelweis University, Budapest, Hungary; 5 Jozsef Fodor Center for Prevention and Healthy Aging, Semmelweis University, Budapest, Hungary; 6 Doctoral College, Health Sciences Division, Semmelweis University, Budapest, Hungary; 7 Stephenson Cancer Center, University of Oklahoma, Oklahoma City, OK USA; 8 Department of Molecular Pharmacology, Albert Einstein College of Medicine, Bronx, NY 10461 USA; 9 Institute for Aging Research, Albert Einstein College of Medicine, Bronx, NY USA; 10 Department of Medicine, Albert Einstein College of Medicine, Bronx, NY USA; 11 Department of Bioengineering, University of California Berkeley, Berkeley, CA 94720 USA; 12 UCL Brain Sciences, University College London, London, UK; 13 University of Helsinki, Helsinki, Finland; 14 Doctoral College/Department of Translational Medicine, International Training Program in Geroscience, Semmelweis University, Budapest, Hungary

**Keywords:** Neuroendocrine theory, Endocrine, Humoral, Geroscience, Senescence, Neurodegeneration, Endothelial dysfunction, Cerebral microcirculation, Cerebral blood flow, Systemic, Regulation, Systemic determinants, Ageing, Vascular aging, Cerebral circulation, Cerebrovascular aging, Endothelial, Neuroinflammation

## Abstract

Aging is a complex biological process that detrimentally affects the brain and cerebrovascular system, contributing to the pathogenesis of age-related diseases like vascular cognitive impairment and dementia (VCID) and Alzheimer’s disease (AD). While cell-autonomous mechanisms that occur within cells, independent of external signals from neighboring cells or systemic factors, account for some aspects of aging, they cannot explain the entire aging process. Non-autonomous, paracrine and endocrine, pathways also play a crucial role in orchestrating brain and vascular aging. The systemic milieu modulates aging through pro-geronic and anti-geronic circulating factors that mediate age-related decline or confer rejuvenative effects. This review explores the impact of systemic factors on cerebrovascular and brain aging, with a particular focus on findings from heterochronic parabiosis, blood exchange, and plasma transfer experiments. We discuss how these factors influence fundamental cellular and molecular processes of aging and impact cerebrovascular endothelial function, neurovascular coupling mechanisms, blood–brain barrier integrity, neuroinflammation, capillary density, and amyloid pathologies, with significant consequences for cognitive function. Additionally, we address the translational potential and challenges of modifying the systemic milieu to promote brain health and prevent age-related cognitive impairment.

## Introduction

The aging global population has created a pressing societal challenge, with numerous nations, including the European Union (EU) member states (e.g., Germany, Italy, Hungary), the United States of America (USA), Japan, and China, experiencing significant demographic shifts [1]. Age-related cognitive impairment and dementia have thus become formidable public health concerns [2–5]. Beyond diminishing individual quality of life, these conditions impose substantial societal and economic burdens. Currently, around 50 million people worldwide live with dementia, a number projected to nearly triple by 2050, reaching 152 million [6–8]. For example, in the USA alone, approximately 6.2 million individuals are affected by dementia, while in Europe, around 10.5 million people are diagnosed, with this number anticipated to grow to 13 million by 2030 [7, 9]. The economic toll of dementia is immense, with the cost of dementia care estimated at $1.3 trillion USD in 2021, a figure expected to rise exponentially as the aging population expands [10]. In addition to economic costs, the emotional toll on families and caregivers is profound, as they navigate both the practical and psychological challenges of providing care. This increasing prevalence of cognitive impairment and dementia necessitates a thorough understanding of the underlying mechanisms to support the development of effective interventions.

Within the scope of age-related cognitive impairment, two primary conditions stand out: vascular cognitive impairment and dementia (VCID) [4, 5, 11, 12] and neurodegeneration, with Alzheimer’s disease (AD) being the most studied [10]. VCID stems from a spectrum of cerebrovascular pathologies and is a significant contributor to cognitive decline, representing a key public health issue [4, 5, 11, 12]. AD, the most common neurodegenerative cause of dementia, is marked by the accumulation of abnormal protein aggregates, including beta-amyloid plaques and tau tangles, causally linked to neuronal dysfunction and loss. The clinical hallmarks of both VCID and AD include progressive memory impairment, cognitive decline, and behavioral changes [4, 5, 11–13]. Both VCID and AD are diseases intrinsically linked to the aging process [14–17], as their pathogenesis involves the cellular and molecular mechanisms of aging. Furthermore, both conditions encompass a spectrum of age-related cerebrovascular pathologies [4, 5, 11–13, 18, 19]. To develop effective interventions for their prevention and treatment, it is imperative to gain a comprehensive understanding of the underlying mechanisms governing the processes of brain and cerebrovascular aging.

Aging itself is a complex biological process with multi-level interactions that transcend individual cells, involving intricate communication between cells, tissues, and organ systems [15, 16, 20, 21]. This process can be dissected into cell-autonomous mechanisms, processes that occur within cells, independent of external signals from neighboring cells or systemic factors, and non-intrinsic mechanisms, which encompass paracrine and endocrine regulation of cell fates and responses [15, 16, 21]. Cell-autonomous mechanisms of aging, spanning a diverse array of processes and pathways [15, 16, 21], include oxidative stress-related pathways, mitochondrial dysfunction, diminished resistance to molecular stressors, genomic instability, telomere attrition, cellular senescence, epigenetic alterations, disruptions in protein homeostasis (referred to as “proteostasis”), and the acquisition of a proinflammatory phenotype [14, 22–43]. These cell-autonomous processes, operative within the cerebral microvasculature and the brain itself, significantly contribute to the decline in cellular functions and are widely recognized as fundamental drivers of vascular and brain aging.

However, it is increasingly evident that in multicellular organisms, the cell-autonomous mechanisms alone are insufficient to explain aging’s complexity at the cell, tissue, or organismal level. Growing evidence highlights the pivotal role of cell extrinsic regulatory mechanisms, which control cell behavior in a tissue, as well as inter-organ communication, and are the major drivers of organismal (whole body) aging and longevity [44–47]. These cell non-autonomous mechanisms orchestrate cellular aging processes in various organs, including the cardiovascular system and the brain [15, 16, 48–63], and are thought to be responsible for the synergistic decline of multiple organ functions with age. Furthermore, they play a central role in shaping the overall aging trajectories of individuals by coordinating aging processes across different organ systems. These pathways originate from various organ systems in the body, including the endocrine system, immune system, adipose tissue, central nervous system, and gastrointestinal tract. Mediators released by these systems enter the bloodstream, exerting systemic effects that simultaneously influence the aging rates of multiple organs. Within this intricate web of non-autonomous mechanisms, the circulation serves as a conduit for various bioactive factors, encompassing hormones, proteins, peptides, lipid mediators, metabolites, and circulating exosomes harboring a diverse array of biomolecules. These circulating factors can be broadly categorized based on their impact on aging processes.

While we acknowledge that no gene or protein evolved explicitly to promote aging, several factors exhibit properties that, when elevated or diminished, tend to accelerate or decelerate age-related decline. Here, we define “pro-geronic” factors as those whose levels typically rise with age and are associated with the emergence of aging phenotypes and accelerated functional decline. Conversely, we refer to “anti-geronic” factors as those that exert rejuvenative effects, often linked to youthful cellular and systemic changes and whose levels decline with aging. This conceptual framework allows us to better study the roles of these factors in tissue homeostasis and aging processes at both the whole-body level and within specific organ systems [64, 65]. Understanding these non-cell-autonomous factors and their contributions to cerebrovascular and brain aging represents an intriguing frontier in geroscience research.

This review aims to navigate the complex landscape of research investigating the role of systemic, non-autonomous signaling pathways in shaping the aging processes, with a specific emphasis on the brain and cerebrovascular system. Through the exploration of experimental paradigms, including heterochronic parabiosis [50], heterochronic blood exchange without sharing organs [66], and plasma transfer and dilution, our aim is to illuminate the interactions between systemic factors and the canonical aging pathways within vital physiological systems. Furthermore, we explore the intricate interplay between the extrinsic to cells signaling pathways and the pathogenic cascades that underlie dysfunction within the brain and cerebral microvasculature. We also discuss the effects of putative pro-geronic and anti-geronic changes in systemic milieu offering a comprehensive perspective on the multifaceted mechanisms governing brain and cerebrovascular aging with insights on rejuvenation.

## Pathophysiology of cerebrovascular and brain aging

Brain aging involves progressive alterations across neural circuits, metabolic processes, structural integrity, and cellular dynamics, all of which culminate in diminished brain function and increased vulnerability to neurodegenerative diseases [23, 67–77]. Neural circuits become disrupted, impairing communication between brain regions and leading to cognitive deficits. Synaptic dysfunction becomes evident, with reduced synaptic density and spine number compromising the brain’s plasticity and adaptability [23, 67–77]. These changes contribute to impaired memory, learning, and executive functions, all hallmarks of cognitive aging [78]. Alongside these synaptic changes, there is a gradual decline in adult neural progenitor cells and a marked decrease in neurogenesis, particularly within neurogenic niches such as the dentate gyrus of the hippocampus and the ventricular-subventricular zone [79, 80]. Neurogenesis is further hampered by age-related alterations in the cellular environment, including changes in the extracellular matrix and the supply of supportive trophic factors [78, 81]. Together, these processes drive cognitive impairments and reduce the brain’s resilience to injuries. 

At the structural level, aging brains show reductions in both gray and white matter. Decreased gray matter density, due to neuronal and synaptic loss, and white matter degradation (primarily through demyelination) impair communication between brain regions and reduce processing speed [23, 67–77]. Pathological brain aging is associated with increased presence of neurofibrillary tangles and amyloid deposits, creating an environment conducive to neurodegenerative processes seen in AD and other dementias. Age-related pathological changes in the brain disrupt cellular homeostasis and induce neuroinflammation, further accelerating cognitive decline [82–84].

The brain has high oxygen and energy demands but has no significant reserves. Preservation of normal brain function thus depends on the maintenance of adequate cerebral blood flow [85–89]. This is ensured by a dense network of cerebral microvessels, with a total length of 600 km in the human brain. Virtually every neuron is supplied by its own capillary. Importantly, aging is associated with multifaceted functional and structural impairment of cerebral microcirculation, which contributes to functional decline in the aging brain and the genesis of age-related cognitive impairment [90–96]. These include a decline in cerebral blood flow and capillary perfusion, impaired neurovascular coupling responses [97–113], capillary rarefaction [110, 114–116] and impaired autoregulation of cerebral blood flow [117–120]. Aging also alters the barrier function of the cerebromicrovascular endothelial cells, which results in blood–brain barrier disruption and increased neuroinflammation [114, 121–124], contributing to the pathogenesis of neurodegenerative diseases. Neural stem cells in the dentate gyrus and the ventricular-subventricular zone are localized to specialized neurovascular niches consisting of a microvascular plexus, in which factors secreted or transported by the cerebromicrovascular endothelial cells regulate activation and differentiation of neural stem cells. Age-related changes in the phenotype and function of cerebromicrovascular endothelial cells contribute to neuronal stem cell dysfunction, reduced neurogenesis, and inadequate repair processes [81, 125–129].

There is also a spectrum of age-related vascular pathologies, ranging from atherosclerosis in the large vessels and consequential development of larger strokes to specific microvascular pathologies (including microinfarcts, microhemorrhages, leukoaraiosis, and cerebral amyloid angiopathy), all of which promote a cognitive decline in elderly patients [119, 130–146]. Strong evidence from epidemiological, clinical and experimental studies suggests that in older adults aging-induced cerebromicrovascular dysfunction and neurovascular injury critically contribute to the pathogenesis of neurodegenerative diseases, including AD [3, 147–150]. Transcriptomic analysis of the aging brain identified complex cell type-specific gene expression signatures, highlighting critical cellular and molecular processes underlying cerebrovascular and brain aging, including mitochondrial and oxidative stress-related processes, cellular senescence, and inflammatory mechanisms [151–160]. Understanding and targeting the cell non-autonomous signaling pathways, which modulate the cerebrovascular and brain aging processes, is expected to have a major role in preserving brain health and preventing cognitive impairment and dementia in older individuals.

## Circulating factors, the systemic milieu, and experimental approaches to study their role in aging

Historically, it has been observed that aging across different cells and organs follows a coordinated trajectory, suggesting an orchestrated aging process regulated by intercellular and inter-organ communication. Early hypotheses proposed that systemic, humoral factors might mediate these aging processes, forming the basis of neuroendocrine theories of aging [161]. According to these theories, hormones and other signaling molecules circulating in the bloodstream play central roles in synchronizing age-related changes throughout the body. A popular version of the neuroendocrine hypothesis of aging suggests that shifts in hormonal balance with age disrupt cellular homeostasis, contributing to the gradual decline observed in various organ systems. Over the past two decades, a significant body of research has substantiated the role of circulating factors (pro- and anti-geronic) in modulating aging. This has been demonstrated through a variety of experimental paradigms, including in vitro studies and in vivo mouse models, which have illuminated the mechanisms through which these factors influence cellular and organismal aging.

**Fig. 1:**
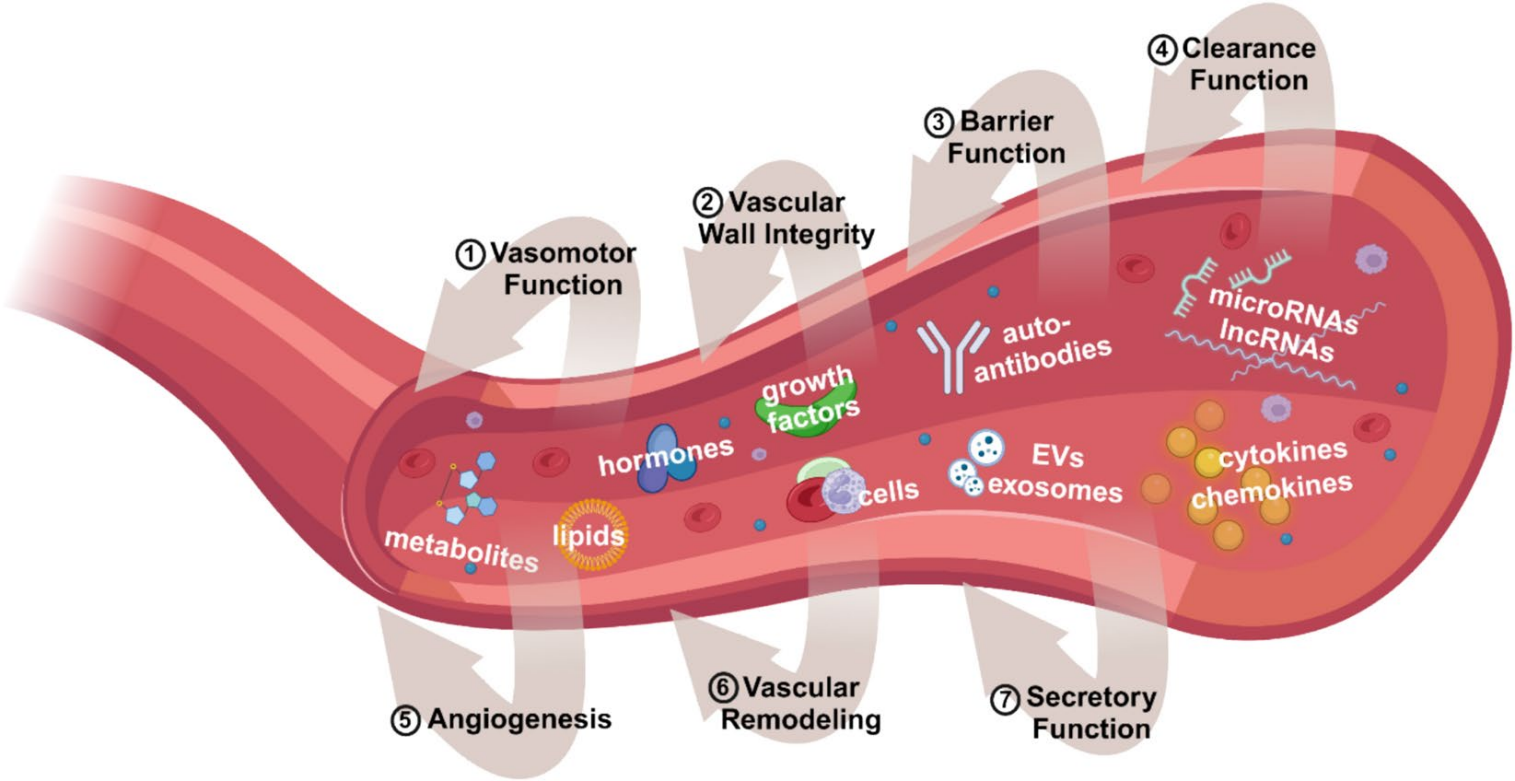
The systemic milieu and its influence on cerebrovascular health. The systemic milieu encompasses a diverse array of bioactive components, including acellular elements (e.g., hormones, cytokines, antibodies, lipids, metabolites, RNA species), cells (e.g., leukocytes, thrombocytes, circulating endothelial progenitor cells), and extracellular vesicles (EVs). These circulating factors collectively modulate age-related changes in the structure, function, and phenotype of the cerebral vasculature. Endothelial cells, as the first line of contact, are profoundly influenced by these circulating factors, which regulate critical functions such as vasomotor control, barrier integrity, molecular clearance, angiogenesis, and secretory activities. Age-related alterations in these systemic factors play a pivotal role in the progression of vascular dysfunction and the development of age-associated chronic diseases. This figure underscores the dynamic interplay between the systemic environment and cerebrovascular health, highlighting the multifaceted contributions of cellular and acellular components to vascular aging and disease

Importantly, experimental work using heterochronic parabiosis, where young and old animals are surgically joined to share a common blood circulation, has demonstrated that mammalian aging has a certain plasticity [63, 79, 162–172]. These studies have demonstrated that exposing aged animals to young blood, or young animals to aged blood, can transpose aging phenotypes [63, 66, 79, 162–175]. Exposure to young blood can mitigate age-related pathologies in older animals, while young animals exposed to aged blood often exhibit accelerated aging phenotypes [170, 176–179]. It has been noted that in the heterochronic parabiosis model, the observed benefits to old mice may not solely arise from exposure to young blood but also from access to young organs, such as the kidneys, liver, and immune system, which contribute to systemic homeostasis and regeneration. Subsequent, better-controlled experiments, in which only the blood was exchanged between young and old mice, highlighted the pivotal role of the aged circulatory milieu in driving rapid and robust geronic phenotypes in young animals, including those affecting the brain [180, 181]. These findings underscore the potency of circulating factors in modulating aging at the cell- and tissue-level across distant organs, demonstrating the systemic impact of the circulatory environment on aging processes. Particularly relevant to our discussion is the role of cell-free plasma compartment, which harbors an array of bioactive molecules, such as proteins, peptides, steroid hormones, lipid mediators, micropeptides, metabolites, bacterial degradation products, and circulating exosomes. Each of these circulating factors has the potential to wield a distinct physiological effect, playing either a promotive or protective role in aging. The diversity of these plasma components emphasizes the broad influence that systemic factors can have on cellular aging, disease progression, and even the rejuvenation of aged tissues. In addition to soluble factors, various circulating cells, such as endothelial progenitor cells and stem cells, are suspended within the plasma, contributing further to the systemic pro- and anti-geronic effects through their regenerative or inflammatory potential (Fig. 1).

The systemic milieu, comprising this intricate array of humoral factors, profoundly influences brain and vascular aging. Vascular cells, including endothelial cells, smooth muscle cells, and pericytes, are in direct contact with these circulating factors, making them particularly susceptible to the influence of pro- and anti-geronic signals (Fig. 1). Moreover, the blood–brain barrier (BBB) serves as a critical interface, allowing selective plasma components to permeate into the brain parenchyma, where they can modulate the aging process in brain cells. Beyond direct effects on brain cells, circulating factors impact brain aging indirectly by altering microvascular function, including blood flow regulation, barrier permeability, and neurovascular coupling. This dynamic interplay between systemic factors and vascular health significantly shapes brain aging and susceptibility to neurodegenerative conditions. Notably, the relationship between systemic factors and brain aging is bidirectional. The aging brain itself influences the systemic milieu through neuroendocrine signals, inflammatory mediators, and other secreted factors that enter the circulation. This reciprocal exchange underscores the interconnectedness of brain and body aging, suggesting that changes in one system can propagate across the organism, impacting overall health and aging trajectories.

**Fig. 2:**
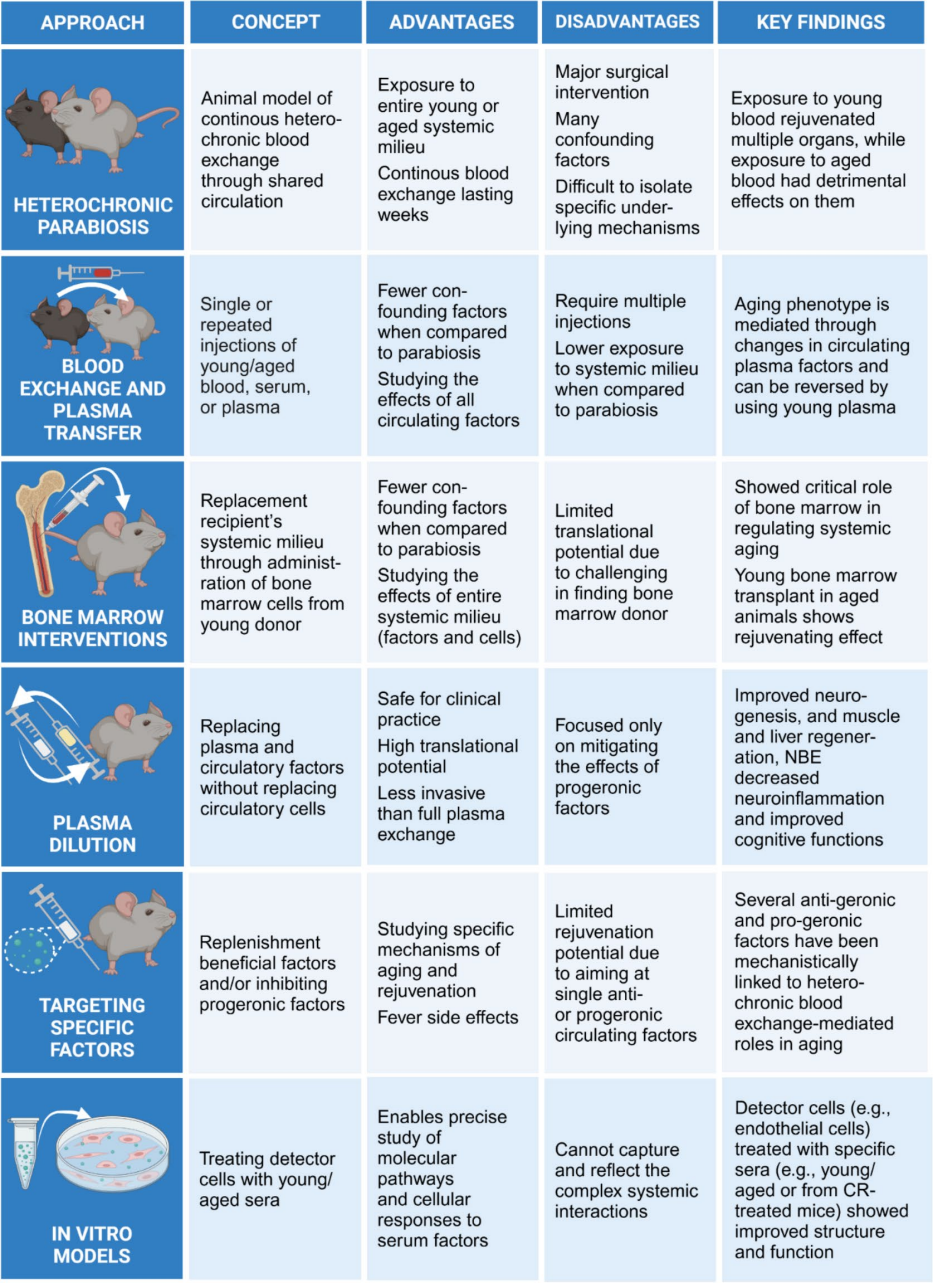
Experimental approaches for investigating cell non-autonomous mechanisms of organismal aging. This figure summarizes six widely utilized methodologies for studying the influence of systemic factors and cell non-autonomous mechanisms on organismal aging. The approaches include heterochronic parabiosis, heterochronic blood and plasma exchange, bone marrow transplantation, plasma dilution, targeted modulation of aging-related circulating factors, and in vitro models using heterochronic sera. The table provides an overview of each method’s core concept, advantages, limitations, and key findings, offering insights into their applications in aging research. These experimental strategies highlight the systemic milieu’s pivotal role in regulating age-related changes in vasculature, cellular function, and overall organismal health

In the following sections, we describe in detail several key experimental paradigms designed to probe the effects of circulating factors on aging. These include heterochronic parabiosis, heterochronic blood apheresis, and plasma transfer experiments. In addition, we discuss plasma dilution and its potential to reduce pro-aging factors by replacing a portion of the blood plasma. Finally, we explore in vitro models that allow researchers to dissect the effects of circulating factors on cellular mechanisms (Fig. 2). Together, these innovative approaches have generated compelling evidence that highlights the profound impacts of pro-geronic and anti-geronic factors, offering critical insights into the regulation of brain and vascular aging.

### Heterochronic parabiosis

Parabiosis, a surgical technique first introduced in the nineteenth century [182], involves joining two animals to create a shared physiological system. This is typically achieved by suturing their skin together, allowing a shared capillary network to form as the join heals over the course of one to two weeks in mammals. The surgical union and the shared circulation of the two organisms allow researchers to investigate the impact of circulating factors from one animal on the physiological and pathophysiological processes in the other**.**

The surgical techniques for parabiosis have undergone refinements over the years. Initially, animals were joined by making abdominal skin incisions and suturing adjacent skin flaps together to create shared capillary network between parabionts. In 1933, Bunster and Meyer introduced the method to additionally suture the animals’ femora and scapulae to provide increased pairing stability and decreased separation risk [183]. Further improvements in the late 1950s included shallow sutures between thoracic and abdominal muscles, reducing the formation of body pockets and improving survival [172, 184]. Subsequent refinements introduced the anastomosis of peritoneal cavities for enhanced circulatory exchange [172, 184]. A recent protocol by Yang and coworkers introduced a series of improvements, including behavior-based animal matching, a longer longitudinal incision, subcutaneous tissue alignment, and metal clips for suture support [185]. While rodents are the most commonly used animals in parabiosis experiments, other models, including chicks, amphibians, fish, and zebrafish, have been utilized to study systemic factors.

**Fig. 3:**
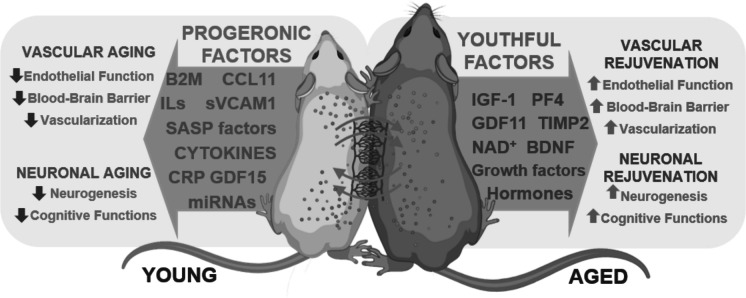
Heterochronic parabiosis and cerebrovascular aging. Heterochronic parabiosis involves surgically connecting two animals of different ages (young and aged, center) to share a common circulatory system, enabling researchers to study the influence of systemic factors on aging processes across tissues and organs. This model has identified several pro-aging (“progeronic”) factors, such as eotaxin- 1 (CCL11), pro-inflammatory cytokines, and SASP components, which contribute to vascular and neuronal aging. Conversely, youthful systemic factors, including growth factors (IGF- 1, BDNF), metabolites (e.g., NAD^+^), and anti-inflammatory mediators, have demonstrated rejuvenating effects, improving endothelial function, neurogenesis, and cognitive performance. The interplay between these factors highlights their critical roles in cerebrovascular and brain aging, presenting novel opportunities for therapeutic intervention to counteract neurovascular decline and age-related cognitive impairments

Over the years, parabiosis has evolved and been employed in various contexts, such as studying the effects of aging, diet, exercise, and diseases on the systemic milieu and organism homeostasis [172]. Notably, these studies led to the discovery of important factors like leptin, which regulates satiety, and parathyroid hypertensive factor, responsible for blood pressure regulation, among others [172, 184]. In heterochronic parabiosis, animals of different ages are paired, providing insights into the influence of age-related factors on the aging process. Heterochronic parabiosis gained renewed interest in the twentieth century due to advancements in geroscience and the pioneering work of McCay in 1957 [186] and Ludwig and Elashoff in 1972 [187], suggesting that pairing with a young partner possesses anti-aging effects (Fig. 3). This pioneering work inspired many groups to explore this direction, which resulted in several studies demonstrating that exposure to young systemic milieu through heterochronic parabiosis lead to amelioration of aging in several organs and systems, including cardiovascular and musculoskeletal, as well as liver, kidneys, and the brain [50, 170, 188, 189]. Of note, there is still an outstanding discrepancy among the few studies of life-extension from heterochronic parabiosis as to whether it works or not. The results from McCay were on too few animals to be conclusive, while Ludwig showed life extension, only in the female rat cohort and not in male rats. A recent study by Yankova, suggests if male mice are joined for three months and then disconnected, there is no life extension in the old partners, and a ~ 6-month reduction in lifepan in the young partners [190]. So, despite over 50 years, the question, does heterochronic parabiosis extend lifespan, is not yet answered.

Despite its utility, the parabiosis model has limitations beyond the distress it initially imposes on joined animals. It is not just blood that is shared - confounding factors include access to co-partner organs and glands (e.g., thyroid, thymus, liver, kidney), exposure to partner’s pheromones due to constant close proximity, and social enrichment, potentially affecting behavior and physical activity. Even a resident tissue may cross-engraft: the partners share circulation for such an extendend period of time that it is possible that a young immune system to variably cross-engraft and prematurely age in the old partner’s “niche,” despite exposure to a mixed young circulation, an idea suggested by Butenko in 1980 [191]. Addressing these challenges requires appropriate control groups and limits interpretation. Additionally, technical difficulties arise because most laboratory equipment is designed for single-animal studies. To circumvent these limitations, innovative approaches have emerged to investigate the systemic milieu’s effects (Fig. 2).

### Blood exchange and plasma transfer

Recent studies showed that repeated injections of young plasma (four to eight i.v. injections over the period of 10–24 days) rejuvenated aged murine brains, while repeated aged plasma injections led to impaired cognitive function, decreased hippocampal neurogenesis, and activation of microglia [62, 176, 192, 193]. On the other hand, a transfer of 50% young blood into old mice did not improve their hippocampal neurogenesis or short term memory, while such single transfer of 50% old blood into young mice, quickly (in 1–2 weeks) diminished cognition, and caused neurogenic decline, as well as aging and senescnece of multiple organ sytems [180]. Therefore, it would be interesting to answer, how the small amounts of young plasma are able to offset aging in the old, when a young animal retaining 50% of its young blood ages and senesces after receiving old blood. While plasma injections have greater translational potential than parabiosis, a procedure that should only be used to get a better understanding of the role of systemic milieu, it remains elusive how long the effects of plasma injections would persist, as well as their effects and safety in humans (Fig. 2). In 2019, FDA warned patients considering young donor plasma infusions for the treatment of age-related diseases, as the proposed intervention has no proven clinical benefits while being associated with serious risks, such as infectious contamination, allergic reactions, overload of the circulatory system, or transfusion-related acute lung injury. Numerous small clinical trials using young donor plasma infusions are currently undergoing. Three recent clinical trials (15–18 participants) showed that repeated plasma infusions are well tolerated and associated with minor adverse effects. Plasma infusions showed moderate improvements for Parkinson’s disease patients, but not in progressive supranuclear palsy [194, 195]. A larger within-patient study, showed that injection of plasma fraction GRF6019 is well tolerated and protected aged patients from the cognitive decline over the time of 5.6 months [196]. The safety and preliminary results of clinical trials on plasma transfer are discussed in further sections of this review.

### Targeting specific factors

Given the risks and limited understanding of clinical benefits associated with whole plasma infusions, a more promising strategy for anti-aging interventions may lie in targeting or restoring specific circulating factors and pathways. Aging is marked by complex shifts in plasma levels of proteins and other soluble mediators and metabolites [161, 197–200]. Experimental paradigms such as heterochronic parabiosis and blood/plasma exchange have shed light on a suite of systemic factors that influence organismal aging. Studies in rodent models have demonstrated that increasing the levels of certain factors (e.g., growth differentiation factor 11 (GDF- 11), oxytocin) or reducing certain aging-elevated factors (e.g., CCL11, osteopontin, beta- 2 microglobulin (B2M)) can promote rejuvenation within specific organs or exert multi-systemic effects [50, 79, 193, 201–204]. Of note, blood shedding of B2M, i.e., the invariant chain of MHC-I, is expected to increase with inflammaging, hence, reducing the systemic levels of B2M might be less effective than targeting the underlying cause of the age-related excess in inflammation. These findings highlight the potential of targeting individual circulating factors to achieve organ-specific or even widespread anti-aging outcomes. While these preclinical studies are promising, there remains uncertainty about whether modulating levels of these factors in humans would yield comparable benefits. Targeting specific molecules or pathways could theoretically replicate the pro-youthful effects observed in parabiosis and plasma exchange without the associated risks of full blood or plasma transfusion. This precision-based approach would allow for interventions tailored to the mechanisms most relevant to specific aspects of aging, thus maximizing efficacy while minimizing unwanted side effects.

However, it is possible that modifying a single factor or pathway will not be sufficient to significantly prevent or reverse the broad effects of aging (Fig. 2). Aging is a multifactorial process involving a complex interplay of pathways and signals. Therefore, a comprehensive strategy that targets multiple factors and pathways may be required to produce substantial and durable anti-aging effects. For example, simultaneously attenuating the aging-elevated TFG-beta signaling through Alk5 inhibitor and adding the aging-diminished oxytocin rejuvenated multiple organs, muscle and brain, in 2-year-old mice [205]. Future research must rigorously investigate which groups of factors, when modulated in concert, could yield optimal outcomes. Further study is also essential to determine safe and effective methods for targeting these factors, whether through pharmacological agents, gene editing, or biotechnological innovations. The review’s later sections delve into the specific roles of individual rejuvenating and pro-geronic factors in cerebrovascular and brain aging, providing a detailed analysis of their effects and potential as therapeutic targets.

### Plasma dilution

Small animal models of human therapeutic plasma exchange (TPE) which dilute plasma and return blood cells synchronically have been used to study heterochronic systemic aging (Fig. 2) [167, 206, 207]. In mice, neutral blood exchange (NBE; replacing plasma with 5% albumin in saline) demonstrated rejuvenating effects on all three germ layer tissues: muscle, liver, and brain [180, 206, 207]. As compared to only partial positive effects of heterochronic parabiosis, NBE shows more robust tissue rejuvenation, where for all measured parameters, e.g., inflammation, fibrosis, tissue maintenance, repair, and functional performance, old animals become statistically indistinguishable from young [173, 175, 206, 207]. For example, as was found in 2004 and repeated in 2011, heterochronic parabiosis improves hippocampal neurogenesis of old mice by ~ 2-fold [79, 173], yet NBE increases it by ~ tenfold to the levels akin of young animals [206]. In addition, NBE signficantly improves cognitive function, decreases neuroinflammation, loweres cellular senescence, and rejuveantes peripheral tissues, increasing muscle repair, and reducing liver adiposity [206, 208]. Interestingly, the attenuation of circulating age-elevated proteins by NBE leads to youthfully restored angiogenic, immune, and tissue remodeling factors, e.g., erythropoietin, CXCL4, CXCR2, MIP2, tissue inhibitor of metalloproteinases 2 (TIMP2), GDF-15, matrix metalloproteinase-9 (MMP-9), and endothelin; while the levels of CCL11 and VCAM-1 remain unaffected. The significance of these factors is discussed in further parts of this review [206]. Importantly, clinical studies suggest that in people rounds of TPE rejuvenate systemic proteome, reduce the protein noise and hence, the biological age. Further, senescence and DNA damage become lower in circulating PBMCs and productive lympohid/myeloid ratios are restored [206, 209]. It has been long known that TPE can remove harmful autoantibodies, complement factors, cytokines, adipokines, triglycerides, and others [210, 211]; it is a standard procedure used as a treatment of thrombocytopenic purpura, Goodpasture syndrome, and other autoimmune disorders [180]. While TPE is relatively safe, its known side effects include changes in blood coagulation, thrombocytopenia, anti-heparin immune response, citrate toxicity, inadvertent activation of complement factors, neutrophils and endothelial cells, leading to renal damage. Other side effects are hemolysis, air embolism, urticaria, muscle cramps, and paresthesias [212, 213]. These side effects occur in 4-7% and 27% of treatments when 5% albumin and fresh frozen plasma (FFP) are used, respectively [214]. It is unknown how frequently one needs to repeat TPE to attenuate tissue aging, e.g., the levels of most cytokines return to baseline within a day or two after a round of TPE, and in relatively healthy adults, cumulative side effects might eventually negate the beneficial rejuvenation effects [209, 214].

### Modifying the systemic milieu through bone marrow interventions

The systemic milieu is intricately connected to the functional status of the bone marrow. Components of the blood affected include the cellular compartment, such as hematopoietic stem cells (HSCs) and immune cells, as well as the myriad of factors produced by these cells, ranging from cytokines to antibodies. The bone marrow serves as the primary site for the production and regulation of these essential blood components. Given this tight connection between bone marrow function and the systemic milieu, it becomes conceivable that modifying the bone marrow could offer a powerful approach to alter the entire systemic environment.

One of the most well-established methods for modifying the systemic milieu involves bone marrow transplantation (BMT). This procedure entails the replacement of an individual’s bone marrow with that from a donor, typically a healthy young donor. BMT has been widely employed in clinical settings to treat various hematological disorders and cancers, primarily by replenishing the patient’s hematopoietic system with healthy donor-derived cells [215]. BMT has provided remarkable insights into the plasticity of the systemic milieu and its potential impact on aging (Fig. 2). Studies have demonstrated that BMT can lead to substantial rejuvenating effects, even in aged recipients. BMT or the delivery of a specific subpopulation of young HSCs into an aged host can result in the rejuvenation of the hematopoietic niche [216] and production of a new generation of blood cells with enhanced regenerative capacity and immune function. This, in turn, can contribute to improved overall health, extended lifespan, and the attenuation of age-related degenerative processes. A study in mice revealed that a partial replacement of aged bone marrow cells with bone marrow cells transplanted from young mice prolonged lifespan for almost 40%, emphasizing anti-aging properties of young BMT [217]. This underscores the profound anti-aging properties harbored by young bone marrow cells. Importantly, the benefits of this intervention extended beyond mere longevity. In aged mice subjected to young BMT, a range of positive outcomes were observed. These included the preservation of physical activity, maintenance of synaptic health, prevention of age-associated cognitive decline, and the attenuation of microglial activation. Strikingly, these beneficial effects persisted even 6 months after the BMT, offering promising and enduring rejuvenation prospects [218]. These results underscore the pivotal role of peripheral HSCs in the aging process and suggest that replacing aged HSCs with their youthful counterparts can effectively slow down the age-related decline in organ functions.

One intriguing aspect of BMT is its potential to influence not only peripheral tissues but also the central nervous system. Emerging evidence suggests that microglia [219], the resident immune cells of the brain, and cerebromicrovascular endothelial cells [220] can be impacted by BMT. When young bone marrow cells are transplanted into aged recipients, the rejuvenating effects extend beyond the bloodstream. Microglia and cerebromicrovascular endothelial cells derived from the transplanted HSCs have been shown to infiltrate and repopulate the brain of transplant recipients [219, 220]. This phenomenon introduces a new level of complexity to the systemic modification of aging, as both the brain’s resident immune cells and capillary endothelial cells play a crucial role in maintaining brain health and cognitive function. The repopulation of microglia and endothelial cells in the brain following BMT offers a potential mechanism through which systemic interventions can exert beneficial effects on microvascular vasodilator and barrier function [221, 222], neuroinflammation [223], synaptic plasticity [224, 225], and overall brain resilience, contributing to improved cognitive outcomes and brain health in aging. Further research in this exciting area may uncover novel strategies to enhance brain function and mitigate age-related neurodegenerative conditions through the modulation of the systemic milieu.

Despite the promising potential of young HSC administration and BMT in rejuvenation and modifying the systemic milieu, there are significant challenges to consider. Identifying suitable donors and minimizing the risks associated with transplantation remain critical concerns, limiting the translational potential of these interventions. Obtaining human HSCs from sources like umbilical cord blood yields relatively low quantities of hematopoietic stem and progenitor cells, often requiring the use of two cords for a single adult transplantation procedure. To overcome this limitation, researchers have proposed various methods to increase the dose of HSCs and enhance their homing and engraftment potential [216, 226]. Additionally, it remains unclear which specific cell populations are responsible for the rejuvenation observed after BMT. Furthermore, achieving anti-aging results could also be possible by revigorating endogenous HSC niches within an aged organism. Several approaches have been proposed to rejuvenate aged HSCs, and one noteworthy factor is osteopontin [227], which has been suggested as a key player in ameliorating aging-related changes in HSCs. While plasma levels of osteopontin increase with age, its expression in bone marrow stroma declines in aged mice, correlating with poorer engraftment and a loss of cell polarity [201, 227]. Nevertheless, it remains to be explored whether and to what extent rejuvenating endogenous HSCs can effectively prevent the aging process. Importantly, BMT not only affects the cellular composition of the blood but also influences the production of factors and signaling molecules by the newly introduced HSCs and their progeny. These factors, which include cytokines, chemokines, and other bioactive molecules, can modulate immune responses, inflammation, and tissue repair mechanisms throughout the body. Understanding their role in the anti-aging effect of BMT warrants further in-depth studies. Additionally, the potential impact of aged HSCs on a young organism has yet to be investigated thoroughly, raising intriguing questions about the bidirectional influence of these cells on aging.

### In vitro* models*

In addition to in vivo experimental paradigms such as heterochronic parabiosis and plasma transfer, researchers have also harnessed in vitro models to dissect the effects of circulating factors on cellular mechanisms underlying aging. A pioneering approach by Rafael de Cabo and colleagues involves the use of serum treatment of detector cells [228–232]. In this method, target cells are exposed to serum collected from humans or laboratory animals of varying ages or those subjected to specific anti-aging interventions, such as caloric restriction [228–232]. This model enables researchers to directly assess the net influence of circulating factors on cellular responses related to aging (Fig. 2) [232]. This approach has proven particularly valuable in demonstrating the existence of circulating mediators that drive the endothelial-protective, anti-geronic effects of caloric restriction [229, 233]. One key advantage of this model is its adaptability: serum samples from animals with various interventions or genetic backgrounds can reveal specific factors driving aging-related effects. This allows researchers to isolate molecular pathways, signaling cascades, and gene expression patterns influenced by circulating factors. As a bridge between whole-organism findings and cellular mechanisms, this model significantly enhances our understanding of the systemic factors governing aging and their implications for age-related diseases.

### Assessment of the pro- and anti-geronic effects of systemic factors

Understanding the rejuvenating (anti-geronic) and aging-promoting (pro-geronic) effects of systemic factors requires a multi-level approach encompassing transcriptomic, molecular, and physiological dimensions. Investigating the rejuvenative effects of young blood or the aging-promoting effects of old blood thus involves a comprehensive analysis across these levels, capturing a holistic view of systemic factors’ impact on aging processes.

#### Transcriptomic level

Transcriptomic studies enable the identification of gene expression changes driven by exposure to young versus old blood. By comparing gene expression profiles, researchers can pinpoint pathways and genes modulated by systemic factors, providing insight into the cellular mechanisms underlying aging and rejuvenation. Advanced analytical tools, such as Ingenuity IPA Upstream Regulator Analysis, can identify upstream regulators, including transcription factors, hormones, cytokines, and microRNAs, that influence these gene expression patterns [171]. This approach reveals potential molecular drivers that mediate the effects of systemic factors, offering a detailed understanding of the regulatory landscape.

#### Molecular level

At the molecular level, assessing specific proteins, metabolites, and other biomolecules associated with aging and rejuvenation sheds light on the biochemical pathways modulated by systemic factors. By examining biomarkers like those related to cellular senescence, oxidative stress, or mitochondrial function, researchers can determine how young blood mitigates or old blood accelerates age-related molecular changes. Quantifying changes in these molecules provides insight into the underlying mechanisms through which systemic factors impact cellular aging.

#### Physiological level

Physiological measurements are essential for understanding the systemic impact of circulating factors on aging and rejuvenation. Parameters such as cognitive function, blood flow, microvascular endothelial health, BBB integrity, long-term potentiation (LTP) and cellular physiological functions such as mitochondrial function and ROS production offer a window into the organism-level effects of young or old blood. In animal models like heterochronic plasma exchange, for example, behavioral tests can reveal the effects of young blood on cognitive aging.

To gain a nuanced understanding of aging modulation by systemic factors, it is critical to integrate findings across the transcriptomic, molecular, and physiological levels. By correlating gene expression changes with molecular marker alterations and physiological outcomes, researchers can achieve a cohesive view of how circulating factors drive the aging process. This integrated approach allows for a detailed map of how pro- and anti-geronic factors shape cellular and organismal aging, advancing our understanding of potential interventions for promoting healthy aging.

## Role of systemic factors present in blood in regulation of aging processes relevant for cerebrovascular and brain aging

Recent research, including studies employing heterochronic parabiosis, blood exchange, and plasma transfer experiments, has provided compelling evidence that the constituents within young blood (or plasma) possess the capacity to reverse age-related alterations observed in various physiological systems. These rejuvenating effects have been observed not only in the cardiovascular system [51, 189, 234, 235] and the brain [48–51, 56, 62, 66] but also in several other vital organs, including the musculoskeletal system [236, 237] and the liver [238]. While it is worth noting that not all investigations have yielded consistent results [239], a substantial body of work supports the notion that young blood harbors factors with anti-aging properties [238]. Conversely, factors present in old blood (or plasma) have been reported to drive aging phenotypes in multiple organs of young experimental animals, including the immune system, brain, and the vasculature [66, 79, 240, 241].

For instance, influential studies conducted by Villeda et al. [62] and Katsimpardi et al. [50] have shed light on the rejuvenating potential of young blood, particularly in the context of the aged hippocampus. Heterochronic parabiosis experiments conducted by these researchers have demonstrated that exposure to the systemic milieu of youthful animals can lead to improvements in neuronal proliferation, differentiation, dendritic spine density, neuronal activity, plasticity, and hippocampus-dependent learning and memory among aged mice [50, 62]. These findings underscore the remarkable impact of young blood on cognitive functions and brain health in aging individuals.

Heterochronic parabiosis studies also provided strong evidence that a relatively short-term exposure to young blood can rejuvenate endothelial function and mitigate vascular phenotypic changes in old mice [51, 62]. Transcriptomic analyses of aortic tissues [51] and cerebromicrovascular endothelial cells [165] have pinpointed numerous genes whose expression levels deviate in the aged phenotype but revert toward a more youthful state when exposed to young blood. Conversely, exposure to old blood can expedite vascular aging processes [241], further emphasizing the pivotal role of blood-based factors in influencing the aging trajectories of vascular endothelial cells. Notably, vascular endothelial cells, which maintain direct contact with all blood components, appear significantly influenced by the factors present in circulation [51, 241]. Encouragingly, the rejuvenation of aging brain functions by these circulating anti-geronic factors, even those that do not readily cross the blood–brain barrier, suggests that the BBB does not impede neuronal rejuvenation by blood-borne factors. These findings align with the growing recognition of the central role played by cerebromicrovascular health in brain aging [17].

In addition, studies employing young plasma transfer in vivo [62, 192, 242–245] and serum treatment approaches in vitro [228, 230–232, 246–248] have further advanced our understanding of the malleability of cellular aging by blood-derived factors. These experiments have demonstrated that aged cells can acquire a more youthful phenotype in response to factors found in the acellular fraction of blood from younger animals. Importantly, this rejuvenation of cellular properties is associated with the restoration of physiological functions in old animals, highlighting the potential for interventions aimed at enhancing the systemic milieu to combat age-related declines.

While these rejuvenating effects on multiple physiological systems are remarkable, understanding how these systemic factors influence the intricate cellular mechanisms of aging is essential for a comprehensive grasp of the rejuvenation process. In the following sections, we will first explore how circulating factors interact with the endothelial cells that form the BBB, the critical interface between the systemic circulation and the brain. We discuss how these endothelial cells, being the first point of contact for circulating factors, play a central role in mediating both direct and indirect effects on brain aging. Following this discussion, we will examine how these systemic factors influence key aging processes such as oxidative stress, mitochondrial function, genomic stability, and other molecular pathways that are pivotal in the progression of cerebrovascular and brain aging.

### Role of the cerebromicrovascular endothelial cells and the blood–brain barrier in mediating the effects of circulating factors on brain aging

The cerebromicrovascular endothelial cells (CMVECs), which line the microvessels of the brain, serve as the first point of contact between the systemic circulation and the CNS [148, 249, 250]. CMVECs, supported by the basement membrane, pericytes, astrocytic end-feet, and extracellular matrix, form the neurovascular unit (NVU) - a structural and functional component of the BBB [250]. Beyond their role as a physical barrier, CMVECs are essential for maintaining brain homeostasis through their secretory, transport, and clearance functions [17, 147, 250, 251]. Equipped with a complex repertoire of receptors and transporters, CMVECs are finely tuned to sense, transduce, and respond to systemic cues.

**Fig. 4:**
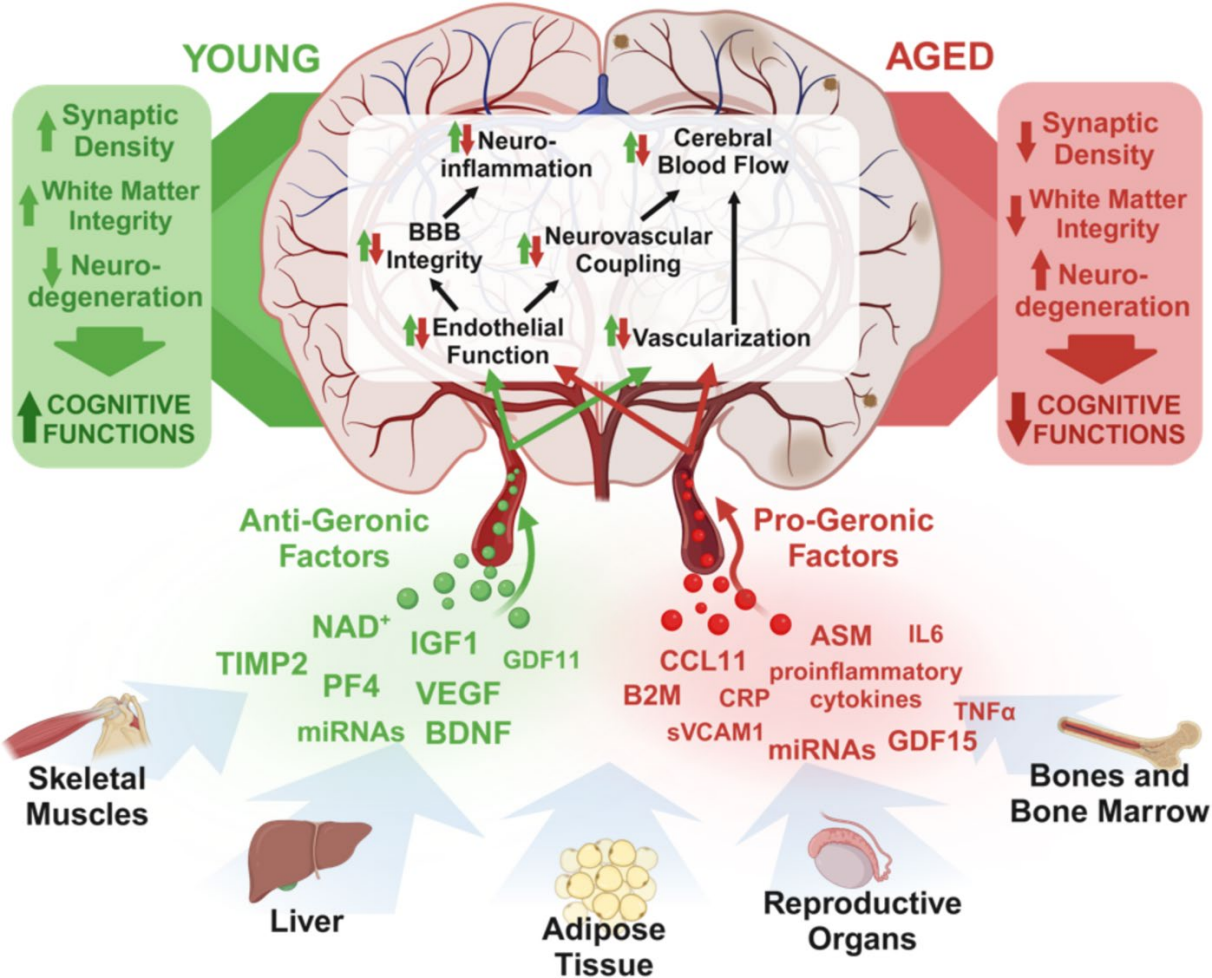
Systemic factors in cerebrovascular and brain aging. The systemic milieu undergoes profound age-related changes that significantly influence cerebrovascular and brain aging. Studies on cell non-autonomous mechanisms have highlighted circulating factors with either anti-geronic or pro-geronic properties. Young blood-enriched factors such as IGF- 1, NAD^+^, VEGF, and GDF11 enhance endothelial function, maintain BBB integrity, and support neurovascular coupling and vascularization. These factors promote cerebrovascular health, improve brain perfusion, and reduce neuroinflammation, collectively preserving synaptic density, white matter integrity, and cognitive function. Additional anti-geronic factors, including TIMP2 and PF4, exert neuroprotective effects critical for cognitive resilience and healthy aging. Conversely, aging is marked by elevated levels of pro-inflammatory cytokines, SASP factors, and harmful microRNAs, alongside other pro-geronic mediators such as CCL11, B2M, and GDF15. These factors drive endothelial dysfunction, BBB breakdown, microvascular rarefaction, and brain hypoperfusion. These cerebrovascular impairments contribute to neuroinflammation, synaptic loss, white matter degradation, and progressive cognitive decline, underpinning age-related neurodegenerative diseases. Abbreviations: BBB, blood–brain barrier; SASP, senescence-associated secretory phenotype; NAD^+^, nicotinamide adenine dinucleotide; IGF- 1, insulin-like growth factor 1; GDF11, growth differentiation factor 11; VEGF, vascular endothelial growth factor; TIMP2, tissue inhibitor of metalloproteinases 2; PF4, platelet factor 4

Heterochronic parabiosis experiments demonstrated that CMVECs are particularly sensitive to anti-geronic factors enriched in young systemic milieu [151, 252]. Single-cell RNA sequencing data indicate that among different brain cell populations, CMVECs exhibit the most pronounced transcriptomic rejuvenation in response to young blood [32], consistent with the idea that circulating factors reach these cells at the highest concentration due to their direct contact with the bloodstream [151, 252]. The CMVECs’ responsiveness to youthful factors has been also observed at the functional level. Heterochronic parabiosis studies demonstrated that 6 weeks of exposure to young blood improved both vasodilatory capacity and barrier integrity in the cerebral microvasculature of aged mice [177, 178]. Conversely, endothelial cells are highly susceptible to the detrimental effects of pro-inflammatory and pro-oxidative factors present in aged blood [163–165, 168, 171]. Studies show that exposure to an aged systemic milieu disrupts CMVEC barrier integrity, impairs their transport and secretory functions, and reduces neurovascular coupling responses in young heterochronic parabionts (Fig. 4) [163, 164]. These disruptions may promote neuroinflammation, neuronal dysfunction, and neurodegeneration. Far from being passive structural elements of the BBB, CMVECs play an active role in brain health by regulating vascular tone to meet metabolic demands, secreting essential signaling molecules, and mediating the selective transport of substances to the brain parenchyma. Positioned at the outermost edge of the NVU, they serve as the first line of response to systemic environmental changes. These findings underscore the pivotal role of CMVECs in brain aging and highlight their dual responsiveness to anti-geronic and pro-geronic factors. Alterations in their sensing, transport, and clearance functions driven by systemic aging processes underscore their central role in the regulation of cerebrovascular and brain health, as discussed further in subsequent subsections.

#### The BBB as a gatekeeper

The BBB acts as a highly selective gatekeeper between the bloodstream and the brain, maintaining brain homeostasis and protecting it from harmful substances [249, 250]. By tightly regulating the passage of molecules, the BBB ensures that essential nutrients and signaling molecules can enter the brain, while preventing the entry of harmful substances. This selective permeability is fundamental for maintaining proper neuronal function and brain homeostasis [249, 250]. As the organism ages, the integrity of the BBB declines, leading to increased permeability [114, 253–257]. This is a hallmark of brain aging and contributes to neuroinflammation, neuronal dysfunction, and neurodegenerative diseases [249, 250]. The breakdown of the BBB allows the entry of harmful substances from the bloodstream into the CNS, including pro-inflammatory cytokines and pro-oxidative mediators, which trigger microglial activation, neuroinflammation, neuronal death, and neurodegeneration [249, 250]. Research, particularly from heterochronic parabiosis experiments, has revealed that the ability of young blood to restore BBB integrity is a key contributor to its anti-aging effects on the CNS [164]. These studies demonstrate that exposure to a youthful systemic milieu enhances BBB function, shielding the brain from harmful circulating factors. Conversely, young animals exposed to aged blood exhibit increased BBB permeability, heightened neuroinflammation, and accelerated brain aging, highlighting the detrimental impact of pro-geronic factors present in aged blood [164]. The interaction between circulating plasma factors and CMVECs plays a key role in regulating what substances can access the brain. Brain endothelial cells can either maintain BBB integrity or contribute to its breakdown, depending on the nature of these circulating factors. Youthful plasma contains anti-geronic factors such as growth factors and hormones (e.g., insulin-like growth factor-1 (IGF-1) [258]), which promote the health of endothelial cells and strengthen the BBB [164]. In contrast, circulating pro-geronic factors found in aged blood, such as inflammatory cytokines and oxidative stress-inducing molecules, can significantly compromise BBB integrity by increasing endothelial permeability (Fig. 4) [259, 260]. Taken together, the regulation of BBB integrity stands out as a critical mechanism through which systemic factors influence brain aging processes. These findings highlight the therapeutic potential of targeting BBB function by modulating these regulatory mechanisms to address brain aging and reduce the risk of neurodegenerative diseases.

#### Endothelial secretory function and brain health

Through their interaction with circulating factors, CMVECs play a direct role in modulating brain aging by altering their secretory profile [15, 16]. Depending on the circulating factors they encounter, brain endothelial cells can either secrete protective molecules that support brain health or release harmful factors that accelerate aging processes. Exposure to anti-geronic circulating factors, such as growth factors and hormones from young blood (e.g., IGF-1) induces endothelial cells to secrete neuroprotective mediators, like NO, which exert pro-survival effects improves vasodilation and ensures adequate blood flow to the brain [109, 261–264]. NO is critical for maintaining vascular tone and preventing age-related decline in neurovascular coupling, which is essential for cognitive health. Endothelial cells exposed to anti-geronic factors also secrete growth factors such as brain-derived neurotrophic factor (BDNF), which support the neurogenic niche [265, 266], particularly in regions like the hippocampus [50]. These endothelium-derived growth factors help sustain neurogenesis and synaptic plasticity, both critical for memory retention and cognitive function [266]. Moreover, in the presence of anti-geronic factors, endothelial cells decrease the secretion of pro-inflammatory cytokines, thereby reducing neuroinflammation and protecting against the development of neurodegenerative diseases [165]. Conversely, when exposed to pro-geronic factors, CMVECs respond by releasing additional pro-geronic molecules, which exacerbate aging-related processes in the brain [152, 165, 266, 267].

#### Endothelial clearance function and brain health

In addition to regulating vascular tone and selective permeability, endothelial cells play a crucial role in the clearance of harmful substances from the blood and brain [249]. The clearance function of endothelial cells is vital for maintaining brain homeostasis and preventing the accumulation of potentially damaging molecules, such as metabolic byproducts, misfolded proteins, and inflammatory agents [249]. This clearance process is particularly important in the context of brain aging, where the efficiency of removing waste products declines, contributing to neurodegenerative conditions like AD [249, 268, 269]. The clearance of substances across the BBB is mediated by various transporters, receptors, and signaling pathways that are regulated by circulating factors [250]. These processes ensure that toxic agents and metabolic wastes are removed from the brain. Dysregulation of these clearance mechanisms during aging can lead to the accumulation of harmful substances in the brain, exacerbating cognitive decline and neurodegenerative diseases [249, 268, 269]. In the context of AD, the accumulation of Aβ plaques in the brain is a hallmark of neurodegeneration [270]. Endothelial cells at the BBB express transport proteins such as low-density lipoprotein receptor-related protein 1 (LRP1), which facilitate the clearance of Aβ from the brain into the bloodstream [271, 272]. Circulating factors, including IGF-1, play a key role in regulating this clearance function [273–275]. Anti-geronic factors, such as IGF-1 and growth differentiation factor-11 (GDF11), have been shown to regulate expression of key transport proteins and Aβ clearance, thereby impacting amyloid plaque formation [273–275]. In contrast, pro-geronic factors found in aged blood, such as inflammatory cytokines (e.g., tumor necrosis factor α (TNF-α), IL-6, IL-1β) and oxidative stress-inducing molecules, impair the clearance function of endothelial cells leading to worsened Aβ clearance and increased deposition of toxic aggregates in the brain [276, 277]. Yet, parabiosis studies have produced mixed results. In aged mutant amyloid precursor protein (APP) mice with established AD-like pathology, exposure to young blood, via parabiosis or intravenous plasma administration, conferred beneficial proteomic and cognitive effect [58]. However, these interventions did not significantly impact amyloid plaque burden, suggesting a selective effect on certain aspects of neurodegeneration, while their influence on Aβ clearance and long-established amyloid pathology remains less clear [58].

In parallel with endothelial-mediated clearance at the BBB, the glymphatic system represents another essential waste elimination pathway in the brain [278–280]. This perivascular network facilitates the convective flow of cerebrospinal fluid (CSF) through the brain parenchyma, enabling the removal of interstitial solutes, including Aβ and tau, via perivascular drainage routes. Importantly, glymphatic function is closely coupled with vascular integrity and endothelial health, as arterial pulsatility [281] and perivascular aquaporin-4 (AQP4) channels [282–285], anchored to astrocytic endfeet, drive the movement of CSF through the glymphatic channels. Age-related endothelial dysfunction, reduced vascular compliance, and neuroinflammation have all been shown to impair glymphatic function, thereby reducing solute clearance and exacerbating protein aggregation in the aging brain [279]. Furthermore, glymphatic and endothelial pathways may interact at multiple levels; for example, impaired endothelial clearance of Aβ may lead to overload of the glymphatic route, and vice versa. Circulating factors that restore endothelial function, such as IGF-1, may also indirectly enhance glymphatic flow [286, 287]. These interlinked systems underscore the importance of maintaining cerebrovascular health for effective waste clearance and highlight the potential of systemic interventions to target both BBB and glymphatic clearance mechanisms [286, 287].

### Effects of circulating factors on cellular oxidative stress

**Fig. 5:**
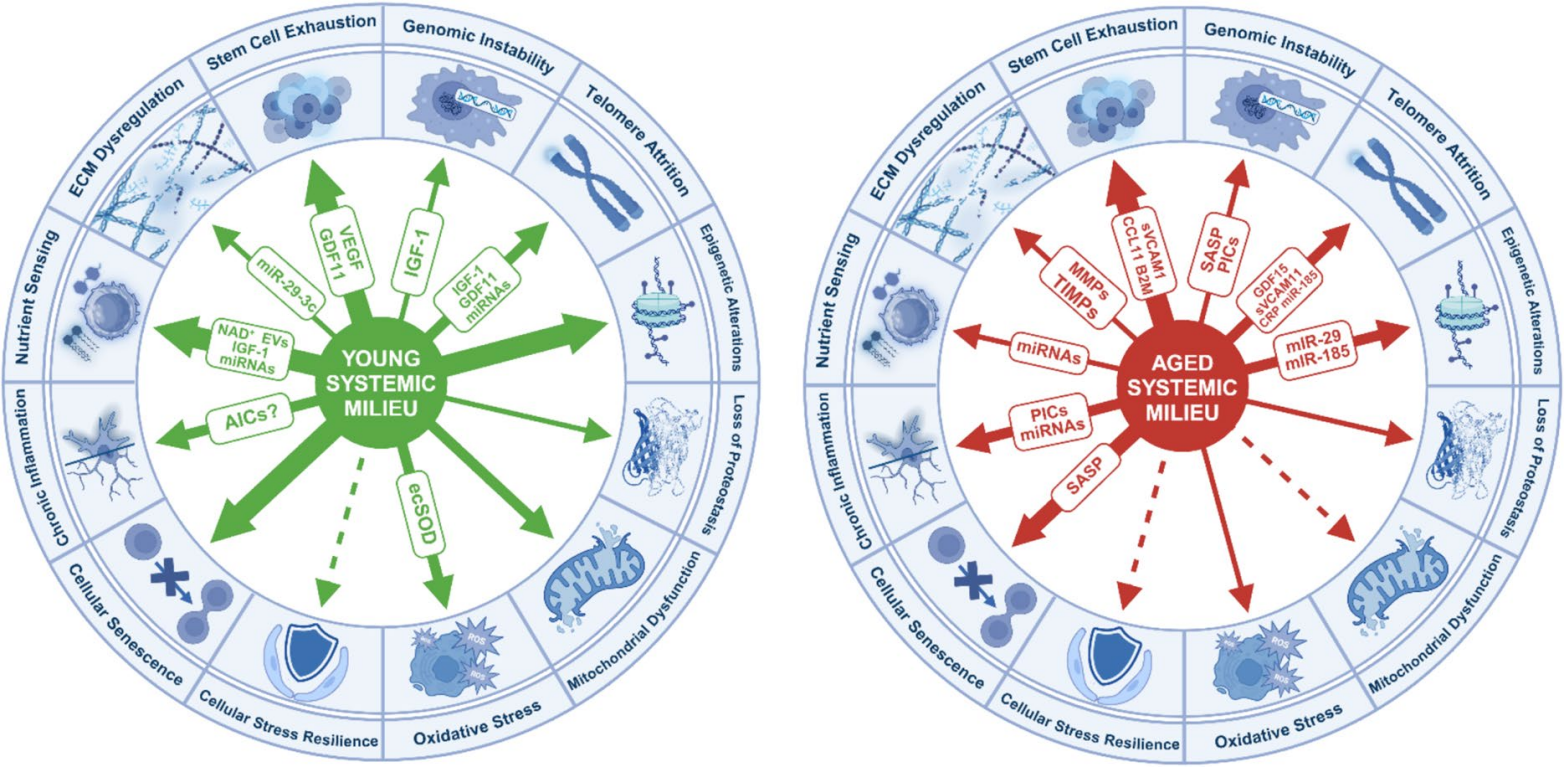
Comparative effects of young and aged systemic milieu on hallmarks of aging. This figure illustrates the differential impact of young (left) and aged (right) systemic milieus on cellular and molecular hallmarks of aging. The young systemic milieu is enriched with anti-geronic factors such as IGF-1, VEGF, GDF11, NAD^+^, and beneficial microRNAs. These factors promote cellular stress resilience, support genomic stability, enhance mitochondrial function, and counteract inflammation and oxidative stress, thereby maintaining tissue homeostasis and delaying aging phenotypes. In contrast, the aged systemic milieu is characterized by pro-geronic factors, including SASP factors, pro-inflammatory cytokines (PICs) and harmful microRNAs (e.g., miR-29, miR-185). These factors exacerbate chronic inflammation, oxidative stress, and mitochondrial dysfunction, leading to genomic instability, cellular senescence, and telomere attrition. Collectively, these age-associated changes drive tissue dysfunction and accelerate aging. The outer ring highlights the core hallmarks of aging affected by these systemic environments. The figure underscores the importance of systemic factors in modulating organismal aging and highlights potential targets for therapeutic interventions aimed at reversing age-related pathologies

Oxidative stress is one of the hallmarks of aging, and it plays an important role in both cardiovascular and cerebrovascular aging (Fig. 5) [17, 103, 105, 107, 110, 111, 113, 257, 288–292] and brain aging [26, 43, 293]. Increased production of reactive oxygen species (ROS) by mitochondrial sources [103, 107, 294], NAD(P)H oxidases (NOX enzymes) [43, 113, 119, 295], and impaired antioxidant defenses (e.g., due to age-related impairment of Nrf2-driven antioxidative pathways [108, 296–300]) contribute to increased cellular oxidative stress in aging. Increased oxidative stress has been causally linked to aging-induced senescence, chronic low-grade inflammation, vasomotor dysfunction, and pathologic remodeling in the aged vasculature [15, 16, 133]. Attenuation of cellular oxidative stress (e.g., by pharmacological induction of Nrf2-driven antioxidative mechanisms, administration of mitochondria-targeted antioxidants, or activation of SIRT1-dependent pathways) was shown to improve endothelium-mediated vasodilation, rescue neurovascular coupling responses and capillarization of the brain, and improve cognitive performance in animal models of aging [100, 102, 103, 107, 111, 119, 290, 301–308]. Additionally, exacerbation of cellular oxidative stress (e.g., by adipogenic [108, 257] or methionine-rich [309–317] diets, or genetic impairment of antioxidant defenses [108, 296, 300, 318]) was shown to accelerate vascular aging processes, impairing endothelium-mediated vasodilation and neurovascular coupling responses, decreasing capillarization of the brain and negatively impacting cognition.

Extant heterochronic parabiosis studies suggest that the presence of young blood decreases oxidative stress in tissues of aged animals [179, 319–321]. Importantly, our recent study provides direct evidence that the presence of young blood in the circulation of aged heterochronic parabiont mice significantly attenuates production of ROS in their aortas, rescuing endothelium-mediated vasorelaxation [179]. A putative young blood-enriched factor, growth differentiation factor 11 can be involved in mediating these improvements [322]. The effects of old blood on endothelial oxidative stress and endothelium-mediated vasorelaxation in the aortas of young heterochronic parabiont mice appear to be less evident [179, 241]. Further studies are evidently needed to determine how young and old blood affect oxidative stress, endothelial function and neurovascular coupling responses in the cerebral microcirculation [179, 241].

On the basis of the transcriptomic changes in the aorta of aged heterochronic parabionts, it seems that young blood-induced mitochondrial rejuvenation is responsible for its marked antioxidative effects [179]. Exposure of aged rodents to young blood was also associated with increased iron-reducing ability in plasma, increased glutathione levels, decreased production of reactive nitrogen species, and decreased lipid peroxidation and protein carboxylation [323]. As an additional mechanism, circulating muscle-derived extracellular SOD (ecSOD) was suggested to contribute to the young blood-mediated reduction of systemic oxidative stress [324].

The concept that circulating factors regulate critical anti-aging, oxidative stress-related pathways in the vasculature is also supported by the findings that treatment with sera obtained from caloric restricted rats and non-human primates attenuate ROS production and up-regulate SIRT1- and Nrf2-regulated antioxidative pathways in cultured endothelial cells [233, 247, 325]. Future studies should identify the individual pro- and anti-geronic factors present in the circulation that regulate endothelial mitochondrial and/or NOX-dependent ROS production and thereby impact endothelial vasodilation and neurovascular function.

### Effects of circulating factors on mitochondrial function and phenotype

The aging processes in both the vascular system [16, 103, 105, 107, 294, 303, 307, 325–333] and the brain [26, 293, 334, 335] are marked by intricate changes in mitochondrial biogenesis and energy metabolism, which associate with significant increases in the production of ROS by mitochondria. Consequently, unraveling the impact of circulating factors on mitochondrial function and phenotype stands as a central inquiry in the field of geroscience.

**Fig. 6:**
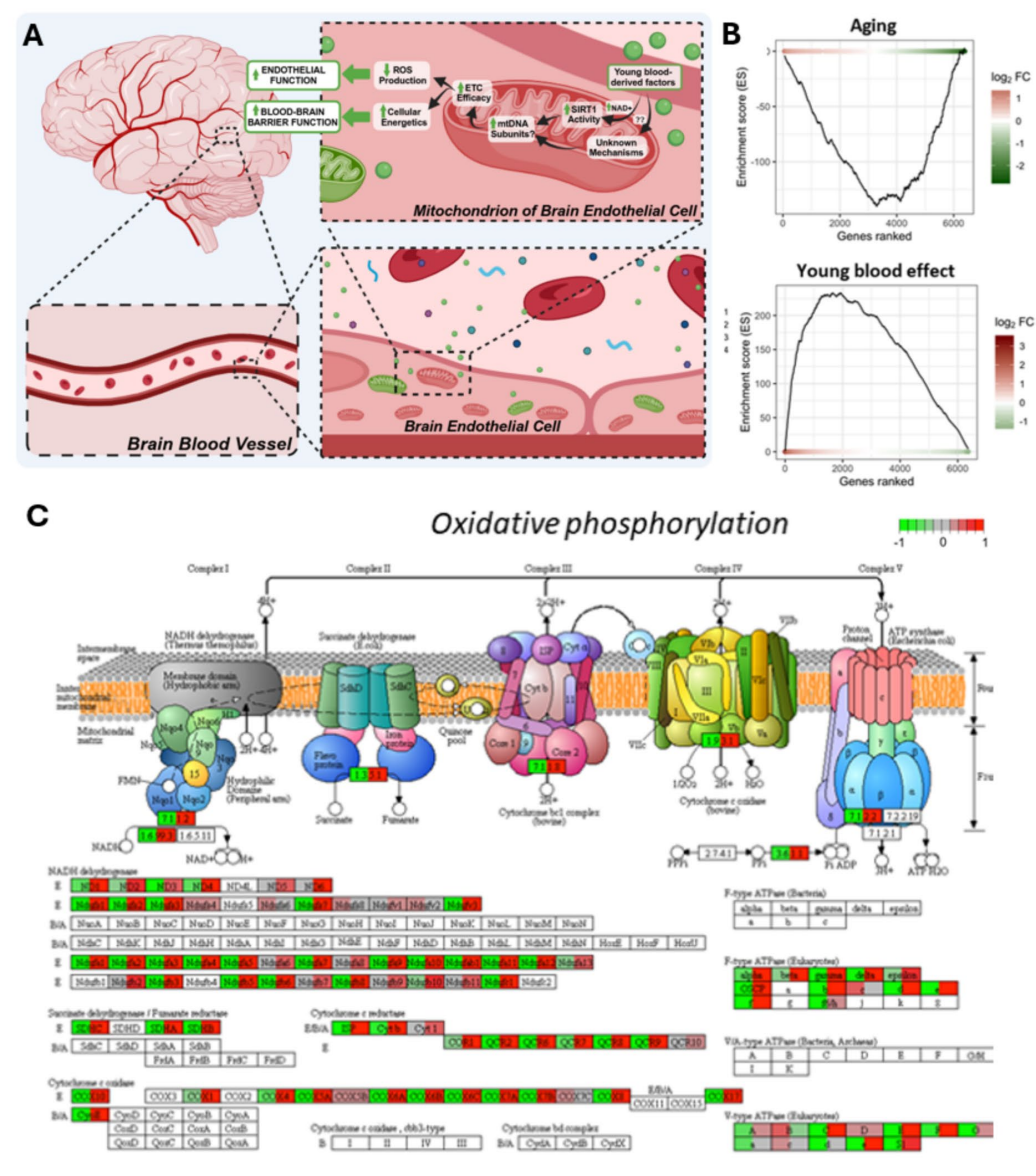
Role of mitochondrial function in brain endothelial cells during aging and young blood-mediated rejuvenation.** A** Schematic representation of the brain microvasculature and its endothelial cells, highlighting the critical role of mitochondrial health in maintaining blood–brain barrier integrity and endothelial vasodilatory function. SIRT1 activation serves as a key regulator of mitochondrial function, cellular energetics, and the modulation of reactive oxygen species (ROS) production in brain endothelial cells. These mitochondrial processes are significantly altered with aging but can be restored by rejuvenating factors present in young blood, which enhance mitochondrial function and cerebromicrovascular health. **B** Gene set enrichment analysis (GSEA) depicting the enrichment profiles of oxidative phosphorylation (OXPHOS)-related genes in brain endothelial cells during aging (top) and following young blood exposure (bottom). Aging leads to the downregulation of OXPHOS-related genes (negative enrichment score), while young blood exposure reverses this trend by upregulating these genes (positive enrichment score), demonstrating the restorative influence of young systemic factors on mitochondrial pathways. **C** Detailed illustration of the oxidative phosphorylation pathway, showing the structural organization of mitochondrial ETC complexes (complexes I–V). Heatmaps represent mitochondrial subunits affected by aging (downregulated: red) and those restored by young blood (upregulated: green). These findings underscore the central role of mitochondrial function in endothelial cell aging and the rejuvenating effects of young systemic factors on mitochondrial health and cerebromicrovascular integrity. The plots showing the mitochondrion-related gene expression changes in heterochronic parabionts used in this figure are reproduced with permission from the publisher. [51]

In a recent investigation, we utilized RNA-seq to scrutinize transcriptomic alterations in the aorta associated with aging and heterochronic parabiosis in mice [51]. This analysis unveiled over 200 discordant genes whose expression levels diverged in the aged phenotype but shifted back toward a more youthful state in response to exposure to young blood in aged heterochronic parabionts [51]. Intriguingly, pathway analysis revealed that the beneficial vascular effects mediated by young blood-regulated genes encompassed mitochondrial rejuvenation (Fig. 6) [51]. Consistent findings were corroborated by subsequent studies that employed single-cell RNA-seq techniques [162, 165]. These studies revealed the rejuvenation of the mitochondrial transcriptome within cerebromicrovascular endothelial cells of aged heterochronic parabionts in response to exposure to young blood [165]. These collective results emphasize the profound impact of systemic factors on mitochondrial function and gene expression, particularly within the context of cerebrovascular health. Mitochondrial rejuvenation through exposure to young blood has also been observed in various organs, including skeletal muscle [237, 336] and liver [238]. In the context of aged mice, exposure to young blood triggers increased mitochondrial remodeling and heightened autophagosomal activity [237, 336]. Conversely, when young mice are exposed to aged blood, a decline in mitochondrial content, alterations in mitochondrial morphology, a significant reduction in the activity of mitochondrial complex IV, and impaired mitochondrial respiration in skeletal muscle are observed [237, 336]. To gain a comprehensive understanding, future studies should aim to identify the specific circulating mediators responsible for mitochondrial rejuvenation by young blood, while also exploring the effects of young and old blood on mitochondrial function and mitochondrial oxidative stress within the brain.

### Effects of circulating factors on genomic instability

Maintaining genomic integrity is essential for cellular function and overall organismal health. DNA damage, mutations, and genomic instability accumulate with age and are associated with functional decline in the vascular [337, 338] and central nervous systems [339], and promote various age-related diseases, including cancer [340], cardiovascular diseases [341], and neurodegenerative disorders [342, 343]. Genomic maintenance mechanisms, such as DNA repair pathways and DNA damage response (DDR) mechanisms, play a critical role in preventing/delaying the development of these age-related diseases [344]. Recent research has started to unveil the influence of circulating factors on genomic maintenance processes [105, 345]. Specifically, studies have explored how systemic factors, such as those present in young blood or plasma (e.g., IGF-1 [346, 347]), may affect DNA repair and genomic stability. Pro-geronic factors present in old blood (including SASP factors and inflammatory cytokines such as TNFα [348]) may impair DNA repair mechanisms and increase DNA damage (Fig. 5). While the influence of circulating factors on genomic maintenance is an exciting area of investigation, several key questions remain. Heterochronic parabiosis experiments and blood exchange studies should provide insights into the potential rejuvenating effects of young blood on genomic maintenance. Future investigations should explore how exposure to a young systemic milieu can impact DNA repair capacity and the accumulation of DNA damage in various tissues, including the vascular system and the brain.

### Effects of circulating factors on telomere shortening

One of the hallmarks of aging, contributing to genomic instability, is telomere attrition [21]. Telomeres are protective caps at the ends of chromosomes that safeguard the integrity of genetic information during cell division. With each round of cell division, telomeres naturally shorten. When telomeres reach a critically short length, they trigger cellular responses such as apoptosis or senescence, in which the cell permanently exits the cell cycle. Senescent cells lose their function and, through the senescence-associated secretory phenotype (SASP), release inflammatory and damaging molecules that negatively affect neighboring cells, exacerbating tissue dysfunction [349–351]. The role of systemic factors in promoting cellular senescence is discussed in detail in a subsequent section.

The rate of telomere shortening varies between cell types and tissues, and although neurons in the human brain exhibit relatively slow telomere attrition due to their limited division, the endothelial cells of the micro- and macrovasculature show a markedly accelerated rate of telomere shortening [337, 338, 352]. This heightened rate of telomere attrition in endothelial cells leads to their senescence, loss of function, and an increased risk of vascular inflammation and cardiovascular diseases. The vulnerability of endothelial cells to telomere shortening is attributed to both intrinsic factors (e.g., oxidative stress and mitochondrial dysfunction) and extrinsic factors (e.g., diet, environmental pollutants, irradiation, and chronic stress) that enhance telomere attrition [353, 354].

Telomeres are maintained by the telomerase complex, which consists of the telomerase reverse transcriptase (TERT), the telomerase RNA component (TERC), and associated proteins. Together, they add repetitive sequences to the ends of telomeres, counteracting the natural shortening that occurs with each cell division. Telomeres are also regulated by proteins such as telomere repeat-binding factors (TRF1 and TRF2) and protection of telomere 1 (POT1), which protect telomeres from degradation and abnormal repair. Several studies, including those using heterochronic parabiosis, suggested that exposure to young blood improves the expression of key components of the telomerase complex in various tissues, including the heart, liver, and kidneys [355, 356]. In contrast, exposure to aged blood has been found to decrease the expression of TERT and TERC, without significantly affecting telomere length [356]. These findings suggest that systemic factors can modulate telomerase activity, which in turn impacts telomere maintenance and cellular aging. Growth factors present in young blood, such as GDF11 and IGF-1, likely play key roles in maintaining telomere length and protecting against telomere shortening [357, 358]. Experimental evidence suggests that GDF11 may be a crucial mediator of telomere maintenance [358]. Studies have shown that the genetic deletion of GDF11 results in telomere shortening by reducing the expression of TERT and TERC [357]. IGF-1 has also been positively correlated with telomere length (Fig. 5) [359–361].

In contrast, systemic pro-geronic factors, particularly those associated with oxidative stress, inflammation, and cellular senescence, are linked to accelerated telomere shortening and dysfunction [362–364]. Factors such as GDF-15, C-reactive protein (CRP), interleukin-1 beta (IL-1β), soluble intercellular adhesion molecule-1 (sICAM-1), and soluble vascular cell adhesion molecule-1 (sVCAM-1) have been associated with reduced telomerase activity and telomere shortening [362–364]. These inflammatory mediators, which are elevated in aging blood, not only contribute to endothelial dysfunction but also drive telomere attrition by increasing oxidative stress and impairing telomerase activity. Emerging research also suggests that microRNAs (miRNAs) regulate telomere dynamics, and their levels fluctuate with age. miRNA-185, for example, has been identified as a negative regulator of telomere maintenance [365]. It targets POT1, a critical protein for telomere protection. As the levels of miRNA-185 increase with aging, it contributes to telomere dysfunction by reducing POT1 expression. This suggests that some age-related miRNAs in circulation may mediate the detrimental effects of aged blood on telomeres. Moreover, other miRNAs have been identified as potential regulators of telomere length, either promoting telomere shortening in aged cells or stabilizing telomeres in younger, healthier cells. While many studies have found correlations between plasma biomarkers and telomere length [359, 366], further research is needed to determine the causality of these factors in modulating telomere dynamics across different tissues and in response to circulating systemic factors.

Although significant progress has been made in understanding how circulating factors modulate telomere dynamics, much remains to be discovered. Future studies should aim to identify the specific pro-geronic and anti-geronic factors in blood that regulate telomerase activity and telomere length across various organs, particularly in the cerebrovascular system and endothelial cells. These insights could lead to novel therapeutic strategies aimed at mitigating age-related telomere shortening and preventing the cellular dysfunctions associated with telomere attrition.

### Effects of circulating factors on epigenetic changes

Epigenetic modifications are pivotal regulators of gene expression and play a crucial role in the aging process [367]. This intricate regulatory system encompasses DNA methylation, histone modifications, and non-coding RNA molecules. All of these mechanisms are influenced by both environmental factors and the systemic milieu, including factors present in circulating blood. Recent studies, particularly those employing heterochronic parabiosis and blood exchange experiments, have provided critical insights into how circulating factors from young blood can rejuvenate epigenetic profiles in aging tissues (Fig. 5) [238, 367].

DNA methylation, the addition of methyl groups to DNA, is a key epigenetic mechanism that either silences or activates genes depending on the location and context of the methylation marks. Aging is associated with specific patterns of DNA hypermethylation at repressive gene sites and hypomethylation at active gene sites, contributing to the altered gene expression patterns seen in age-related diseases [367–372]. Studies employing heterochronic parabiosis and blood exchange experiments have revealed that exposure to young blood can influence DNA methylation patterns in various tissues [238].

In recent years, epigenetic clocks have emerged as powerful biomarkers of biological age, offering means to estimate an individual’s biological aging trajectory relative to their chronological age [368–372]. These clocks are based on DNA methylation patterns at specific CpG sites, which reflect cumulative environmental and genetic influences on aging. Biological age assessments have been further validated in studies using heterochronic parabiosis and blood exchange models, where exposure to young blood reduced the epigenetic age of aged animals. Remarkably, exposure to young blood has demonstrated a profound effect in reducing the epigenetic age of the liver, as evidenced by multiple epigenetic clock models [238]. Interestingly, these effects persisted even after the experimental pairing was ended, indicating the robustness of young blood’s impact on biological age [238]. Additionally, Horvath’s epigenetic clock showed a significant reduction in biological age in multiple organs of rats treated with young porcine plasma, suggesting that the rejuvenating effects of young blood extend across species [243]. In contrast, exposure to aged blood appears to accelerate biological aging; however, the detrimental effects seem to diminish once the exposure to aged blood is discontinued, suggesting a potential for reversal when the pro-geronic systemic environment is no longer present [373]. A significant recent study also investigated the impact of weekly injections of human umbilical cord plasma concentrate on elderly human subjects over a 10-week duration [374]. This intervention was shown to effectively reduce GrimAge, a biological age measure based on DNA methylation, highlighting the translational potential of this approach [374]. These findings strongly indicate that alterations in DNA methylation patterns may play a substantial role in contributing to the enduring rejuvenating impact observed in various aging-related phenotypes, even after only a short-term exposure to young blood.

Clinical studies involving patients undergoing hematopoietic stem cell transplantation (HSCT) extend these findings. Similarly to preclinical models, patients experience significant epigenetic rejuvenation following the procedure, as demonstrated by reductions in biological age based on epigenetic clocks. This rejuvenation is driven by the infusion of youthful hematopoietic cells from the donor, which carry a younger epigenetic signature that temporarily resets the epigenetic landscape of the recipient. However, this epigenetic rejuvenation tends to diminish over time, with biological age gradually reverting towards the recipient’s chronological age. This suggests that while the transplant offers short-term epigenetic benefits, the recipient’s systemic environment plays a crucial role in determining the persistence of these effects [375, 376].

Another key epigenetic modification affected by aging is the reduction of 5-hydroxymethylcytosine (5 hmC), which is catalyzed by the enzyme ten-eleven translocation methylcytosine dioxygenase 2 (Tet2). Decreased Tet2 expression and 5 hmC levels in the hippocampus have been associated with reduced neurogenesis and impaired cognitive function in aging [277, 356, 377, 378]. Interestingly, heterochronic parabiosis experiments have shown that exposure to young blood restores Tet2 expression and 5 hmC levels in the hippocampus, which may contribute to the observed improvements in cognitive function in aged heterochronic parabionts [49].

Histone modifications are another critical epigenetic mechanism regulating gene expression through alterations in chromatin structure [379, 380]. These post-translational modifications include acetylation, methylation, phosphorylation, ubiquitination, and sumoylation, all of which can significantly influence chromatin compaction and gene transcription. Aging is associated with widespread changes in histone modification patterns, including the hyperacetylation of certain histones and loss of histone methylation at specific sites [367, 379–381]. Emerging evidence suggests that circulating factors, particularly those enriched in young blood, may modulate these age-related histone changes [367]. However, while the potential impact of parabiosis on histone modifications is intriguing, these effects remain poorly characterized, underscoring the need for further research to elucidate the underlying mechanisms.

Central to the epigenetic regulation of mRNA transcription are the sirtuins, particularly SIRT1, a NAD⁺-dependent deacetylase that promotes chromatin compaction by deacetylating histones, thereby suppressing aberrant gene transcription. SIRT1 is crucial for maintaining genomic stability, mitochondrial biogenesis, and pathways mediating oxidative stress resistance [382, 383] including DNA repair, all of which are integral to promoting healthy aging and longevity, and prevention of the pathogenesis of age-related diseases. Previous investigations have explored how circulating factors, in the context of caloric restriction-mediated rejuvenation, regulate SIRT1-mediated cellular processes and, by extension, the epigenetic mechanisms driving rejuvenation [228, 230–232, 247, 248, 384]. Specifically, it was demonstrated that serum factors derived from caloric-restricted rodents, monkeys, and humans can upregulate SIRT1 and induce anti-aging phenotypic changes in cultured cells, including attenuation of mitochondrial oxidative stress [228, 230–232, 247, 248, 384]. However, it should be noted that while the role of SIRT1 and sirtuins in cellular rejuvenation is an intriguing area of research, our understanding of their specific contributions to the rejuvenating effects of heterochronic parabiosis remains incomplete [51]. Understanding how circulating factors affect histone modifications and sirtuin activity could provide valuable insights into the molecular pathways underlying the rejuvenating effects of young blood and offer new avenues for therapeutic interventions aimed at promoting healthy aging and combating age-related diseases.

Non-coding RNAs, including miRNAs and long non-coding RNAs (lncRNAs), have emerged as key epigenetic regulators of gene expression during aging [367, 385–387]. Research has increasingly highlighted their role as central players in the intricate interplay between circulating factors and cellular processes relevant to aging [388]. Emerging evidence from heterochronic parabiosis experiments suggests that circulating factors, such as those present in young blood, can modulate the expression and activity of non-coding RNAs [388]. These regulatory interactions have been linked to various aspects of aging, including changes in the extracellular matrix and secretion pathways [388]. Importantly, miRNAs whose expression changes with age in multiple organs are also present in circulating plasma and extracellular vesicles [388]. MiRNA-29c-3p, a member of the miR-29 family, has garnered attention for its role in the aging process and its potential implications for age-related diseases [389, 390]. Studies have shown that miR-29c-3p exhibits the most significant correlation with aging, particularly in solid organs [388]. Furthermore, miR-29c-3p has been associated with promoting cellular senescence [390] and alterations in miR-29c-3p levels have been observed in conditions like Parkinson’s disease, highlighting its relevance in the context of neurodegenerative disorders [389]. Notably, in the context of heterochronic parabiosis experiments in mice, miR-29c-3p stands out as the miRNA most prominently restored to levels akin to those found in young tissues [388]. This finding suggests that regulation of miR-29c-3p by youthful systemic factors may play a role in the rejuvenation of aging-related phenotypes. Conversely, age-related increases in miRNAs such as miRNA-185, which contribute to telomere dysfunction, may be mitigated by young blood. In addition to miRNAs, lncRNAs have emerged as key players in the regulation of gene expression and chromatin remodeling during aging [387, 391, 392]. For example, changes in the expression of lncRNAs have been linked to extracellular matrix remodeling, a process central to both tissue aging and rejuvenation [393–398]. As circulating factors can influence lncRNA expression, this offers an exciting avenue for future research into how systemic factors modulate epigenetic changes in aging.

### Effects of circulating factors on cellular senescence

Cellular senescence, characterized by a DNA damage-induced cellular stress response resulting in irreversible cell cycle arrest, plays a pivotal role in the aging process and age-related diseases [37, 399–406]. Aging is hallmarked by the progressive accumulation of senescent cells, recognized as a driving force behind aging and a contributing factor to age-related diseases [407]. In the context of cerebrovascular and brain aging, there is a notable increase in the prevalence of senescent endothelial cells within the cerebral microcirculation, accompanied by the presence of senescent microglia and oligodendrocytes in the brain parenchyma aging cell reference [37, 153, 408]. Studies in mouse models have demonstrated that interventions targeting senescent cells, both genetically and pharmacologically, can extend health span and lifespan [409], enhance cognitive function [410–414], improve cerebromicrovascular function [256, 415], and enhance the regulation of cerebral blood flow [416–419]. These promising findings highlight cellular senescence as a potential therapeutic target to combat age-related decline in cerebrovascular and brain health.

The role of senescent cells in the aging brain is multifaceted. First, senescent cells, due to their functional, morphological, and phenotypic alterations, contribute to the deterioration of organs as individuals age [407]. In the brain, senescent microvascular endothelial cells have been causally linked to impairments in microvascular function, including disruptions of the BBB [256, 420, 421] and compromised endothelium-mediated neurovascular coupling responses [416–419]. Senescent cells exhibit irreversible cell cycle arrest impairing angiogenesis, and their presence within the microcirculatory network may contribute to pathological remodeling of the cerebral microcirculation, including microvascular rarefaction [416, 417].

Second, senescent cells are characterized by the secretion of a range of proinflammatory mediators collectively known as the senescence-associated secretory phenotype (SASP), which include chemokines (e.g., CCL3, CCL4, CCL11), interferons (INF-γ), interleukins (e.g., IL-1a and IL-1β, IL-6, IL-10), other pro-inflammatory factors (e.g., MCP-1, TNFα), growth factors, proteases, and soluble receptors and ligands [422, 423]. Aged endothelial cells, in particular, often undergo cellular senescence and release various proinflammatory molecules, including pro-inflammatory cytokines, activin A, growth differentiation factor-15 (GDF15), IL8, OPN, and PAI1 [296, 424, 425]. These secreted factors can act in a paracrine manner, influencing the function of neighboring cells and amplifying tissue dysfunction even when only a small number of senescent cells are present. Heterochronic parabiosis has been shown to reprogram the mouse brain transcriptome by attenuating aging signatures in multiple cell types [151].

Third, as individuals age, cellular senescence becomes significantly more prevalent across various tissues, including liver, kidneys, pancreas, spleen, skin, brain, adipose tissue, and in circulating cells (e.g., lymphocytes), leading to elevated circulating levels of proinflammatory SASP factors like IL-6, TNFα, and chemokines [181, 422, 426, 427]. Until recently, it was not fully understood whether cellular senescence and SASP factors can mediate aging in endocrine manner. However, in a recent study, Jeon et al. demonstrated that aged plasma could induce senescence in cultured cells and that a single blood exchange between aged and young mice could trigger cellular senescence and upregulate senescence-associated gene expression in multiple organs, including the heart, liver, kidneys, and skeletal muscles of young mice [181]. This indicates that circulating factors from aged blood can drive senescence in distant tissues, further reinforcing the concept that SASP factors contribute to systemic aging. Further studies, using heterochronic parabiosis models and in vitro experiments using senescent cell-conditioned media and plasma from aged donors, revealed that detrimental effects of aged blood can be, at least in part, attributed to pro-inflammatory mediators originating from senescent cells [170, 428, 429]. Several SASP factors have been directly linked to age-related diseases. One notable example is IL-6, a cytokine secreted by senescent endothelial cells. Elevated plasma IL-6 levels in aging [430, 431] have been associated with chronic inflammation, cardiovascular diseases, reduced physical activity, and cognitive decline [431–434]. The pro-aging effects of SASP factors [428] and their impact on cerebrovascular function are further discussed in section "Cytokines, chemokines, and other inflammatory factors".

On the other hand, studies involving heterochronic parabiosis and heterochronic blood exchange have revealed that exposure to a young systemic environment can lead to the reduction of cellular senescence across multiple tissues [170, 181]. This rejuvenation is accompanied by decreased expression of the p16 gene and lower levels of SASP factors in organs such as the lungs, kidneys, forebrain, adipose tissue, liver [170, 244, 435] and endothelial cells [165]. Additionally, exposure to young blood has been shown to alleviate the senescence burden in various organs, including the spleen, skin, kidneys, brain, and liver, further underscoring the systemic rejuvenating effects of a youthful systemic milieu [244, 252, 436].

The exact mechanisms through which progeronic and anti-geronic factors in the circulation regulate cellular senescence and the sequence of events remain incompletely understood. On one hand, it is plausible that the young humoral environment reduces ROS production, diminishes DNA damage, and potentially prevents the induction of cellular senescence in the brain and other organs. Furthermore, it may facilitate the clearance of senescent cells. Importantly, the reduction in senescent cell burden in peripheral organs is expected to contribute to the mitigation of circulating pro-inflammatory mediators, which, in turn, is likely to exert protective effects on the brain. This intricate interplay between circulating factors and senescence represents a promising avenue for further exploration in the quest to unravel the complexities of cerebrovascular and brain aging.

### Effects of circulating factors on inflammatory pathways

A hallmark of aging is the presence of sterile, low-grade inflammation—commonly referred to as 'inflammaging'—characterized by chronically elevated levels of proinflammatory mediators in the circulation [21, 437, 438]. Inflammation, while serving as a protective mechanism against exogenous noxa and infectious agents, becomes maladaptive during aging. As the body ages, the inflammatory response becomes chronic and dysregulated, contributing to tissue damage rather than repair, leading to a range of age-related pathologies [438]. In the absence of infectious stimuli, chronic proinflammatory responses lead to impairments at both the cellular and organ levels, manifesting as cellular dysfunction, increased senescence, apoptosis, and the development of fibrosis and tissue atrophy [439]. The pathogenesis of inflammaging is complex, involving multiple contributing factors [438, 440]. These include ROS-induced overactivation of the NF-κB (nuclear factor kappa-B) pathway, the accumulation of senescent cells, activation of the inflammasome complex, pattern recognition receptors (PRRs) such as Toll-like and NOD-like receptors, enhanced gut permeability, immune system dysfunction, and reduced anti-inflammatory responses [437, 438, 440–442].

One area where inflammaging has pronounced effects is in the vascular system, where increased inflammation is causally linked to the progression of numerous age-related vascular pathologies, including endothelial dysfunction, arterial stiffening, atherosclerosis, atherothrombosis, and microvascular dysfunction [16, 441, 443]. Vascular inflammation shares canonical inflammatory pathways with systemic aging processes [16, 444, 445]. Vascular aging is associated with mitochondrial dysfunction in the endothelium, resulting in the excessive production of reactive oxygen species, such as H_2_O_2_, which in turn activates the pro-inflammatory NF-kB pathway. The chronically activated NF-kB pathway within endothelial cells further exacerbates inflammation through increasing the expression of proinflammatory mediators and adhesion molecules. Additionally, PPRs, including the NLRP3 inflammasome, exacerbate vascular inflammation by promoting the release of pro-inflammatory cytokines, which collectively contribute to endothelial dysfunction, loss of barrier function, and vascular remodeling [442, 446–449]. A compelling mechanistic study by Josephson et al. demonstrated that the proinflammatory state is transmissible through systemic circulation [450]. In this transgenic parabiosis study, the proinflammatory phenotype of an NF-κB-deficient mouse was transmitted to its wild-type partner through circulating factors [450]. This raised the question of whether the aged blood-mediated detrimental effects seen in heterochronic parabiosis and blood exchange experiments are also due to systemic transmission of inflammation. Indeed, several research groups have demonstrated that inflammaging negatively impacts young cells, tissues, and organs through cell non-autonomous mechanisms, confirming the systemic nature of age-related inflammation [79, 165, 181].

Comparative analysis of plasma factors enriched in aged blood has identified several proinflammatory mediators involved in the propagation of inflammaging, including CCL2, CCL11, CCL12, CCL19, haptoglobin, and B2M [79]. Some of these factors, notably CCL11, B2M, and transforming growth factor-β1 (TGF-β1), have been causally linked to decreased neurogenesis and cognitive decline in young mice subjected to heterochronic parabiosis [61, 79, 451]. Conversely, exposure to young blood has been shown to attenuate inflammation, with systemic anti-inflammatory effects observed in aged parabionts [165, 435], suggesting that rejuvenating factors may counteract proinflammatory mediators. It remains crucial to determine whether the anti-inflammatory effects of young blood predominantly stem from the action of specific anti-inflammatory mediators (e.g., IL-4, IL-10, lipoxins, resolvins) or simply from the dilution of proinflammatory factors present in aged blood.

It is equally important to assess whether age-related systemic proinflammatory changes contribute to vascular aging. A recent study by Ximerakis et al. demonstrated that brain endothelial cells are among the primary recipients of the rejuvenating effects of young blood [165]. Transcriptomic analysis of these cells revealed significant reductions in TNF-α and NF-κB signaling pathways upon exposure to young blood [165]. Indirect evidence from the analysis of young blood-mediated vascular transcriptomic signatures suggests that the NAD+ -dependent histone deacetylase, SIRT1, known for its strong vascular anti-inflammatory properties, may partially explain the anti-inflammatory effects of young blood on endothelial cells. SIRT1 plays a crucial role in modulating inflammatory pathways, and its activation in response to youthful systemic factors could help attenuate age-related chronic inflammation, thereby preserving endothelial function. Additionally, anti-inflammatory cytokines such as IL-4, IL-10, IL-13, IL-1ra, miR-126-3p [441], or TGF-β1 [452], may contribute to the anti-inflammatory properties of young blood. In contrast, exposure to aged blood induces proinflammatory pathways in brain endothelial cells [165], contributing to neuroinflammation and vascular aging. Potential mediators [79] may include soluble VCAM-1 [193], microvesicles [453], and miRNAs such as miR-21-5p [441], which have been implicated in the inflammatory response observed in aged organisms. Identifying the systemic factors responsible for mediating vascular inflammation remains a critical area of research for understanding the progression of age-related vascular dysfunction and its broader impact on brain health.

### Effects of circulating factors on stem cell dysfunction

Aging is marked by a decline in tissue and organ regeneration, which is closely tied to the decreased number and functionality of stem cells (SCs) across the body [16, 21, 173]. SCs, known for their high proliferative capacity and ability to self-renew, are responsible for generating differentiated cells required for tissue maintenance and repair. While organs like the liver or skin exhibit a high regenerative capacity with frequent cellular turnover, others, such as the brain or kidneys, have limited regenerative potential [454]. Stem cells reside in specialized microenvironments known as stem cell niches (SCNs), which maintain SC homeostasis, regulate their fate, and protect them from apoptosis and exhaustion [174, 266]. A typical SCN consists of SCs surrounded by microvessels, stromal cells, extracellular matrix components and neural inputs [455]. Although the proximity of SCNs to blood vessels allows them to respond to systemic cues [266], it also makes them vulnerable to detrimental circulating factors, which can impair self-renewal, promote SC senescence, and disrupt cell differentiation, potentially leading to tissue dysfunction [456].

Evidence suggests that cell non-autonomous mechanisms play a critical role in regulating SC function and aging [174]. Studies involving heterochronic parabiosis, heterochronic blood exchange, and transgenic parabiosis have shown that exposure to aged blood-enriched with proinflammatory factors—negatively impacts SCNs, leading to a reduction in SC numbers and function [66, 239, 457]. These changes hinder tissue regeneration, repair, and overall function. These detrimental effects have been observed in SCNs of bones, skeletal muscles, bone marrow, liver, and the brain [176, 188, 252]. Conversely, exposure to young blood has been shown to rejuvenate SCNs across various organs, including bone marrow, liver, pancreas, skin, skeletal muscles, bones, and the brain [62, 188, 252, 457]. Certain circulating factors, such as GDF-11, testosterone, CCL11, and B2M, have been implicated in the systemic regulation of SC niche aging; however, more research is needed to identify additional factors and pathways involved [60, 61, 176, 458].

In the brain, neurons, the post-mitotic cells responsible for executing brain functions, are primarily formed during embryonic and perinatal development, with a smaller portion generated throughout life via adult neurogenesis. This process is supported by neural stem cell (NSC) niches located in the subventricular zone and the subgranular zone of the hippocampus. However, adult neurogenesis declines with age, primarily due to deep quiescence and a reduction in NSC numbers [459]. Evidence suggests that these age-related changes are at least partially mediated by systemic factors. Exposure to an aged systemic milieu impairs the NSC niche, leading to reduced numbers of NSCs, neural progenitor cells, and proliferating cells, ultimately resulting in cognitive decline in young animals exposed to aged blood through heterochronic parabiosis [79, 176, 180, 193]. Proinflammatory factors, including CCL11, B2M, and sVCAM1, have been causally linked to the detrimental effects of an aged systemic milieu [79, 180, 193, 451] on the NSC niche. Conversely, factors enriched in young blood, such as GDF-11, have been shown to rejuvenate the aged NSC niche, promoting increased neurogenesis and cognitive improvements [49, 50, 460]. Additionally, young systemic factors enhance vascularization and blood perfusion in the subventricular zone, with GDF- 11 and VEGF emerging as potential mediators of cerebrovascular rejuvenation [203, 461]. Moreover, the beneficial effects of the young systemic milieu have been demonstrated in the model of spinal cord injury, where the exposure to young blood increased the number of proliferating oligodendrocyte progenitor cells and vascular density within the spinal lesion, further emphasizing proangiogenic and regenerative effects of young blood in the central nervous system [462].

Aging is also linked to increased endothelial apoptosis and reduced neovascularization, which compromises tissue vascularization and perfusion [16]. Adult neovascularization is largely dependent on circulating endothelial progenitor cells (EPCs). However, EPC numbers and function decline with age [463, 464], influenced by intrinsic factors such as increased ROS [465–467], telomere shortening, and senescence, as well as extrinsic factors like proinflammatory mediators and decreased levels of pro-angiogenic and homing signals [463, 464, 468–472]. These changes lead to impaired endothelial regeneration and neovascularization. EPCs originate from hematopoietic stem cell (HSC) niches in the bone marrow. Recent studies using heterochronic parabiosis have shown that HSC niches are particularly responsive to rejuvenation by a young systemic milieu [469]. Exposure to young blood restores the transcriptomic profile of aged hematopoietic stem and progenitor cells, increasing homeostatic cytokine expression and anti-inflammatory targets of TNF-α signaling [252, 457]. In contrast, HSPCs from young parabionts exposed to aged blood exhibit accelerated aging transcriptomes, demonstrating that SC niche aging is driven by systemic factors [457]. The age-related decline in EPCs, as well as their regulation by systemic factors, has also been shown to influence neovascularization and endothelial homeostasis [468, 470, 473–478].

Several systemic factors have been directly implicated in the regulation of EPCs. Factors such as hematopoietic growth factor (GM-CSF) [479–483], GH and IGF-1 [468, 475–478] are known to increase EPC numbers, while VEGF [484, 485], platelet-derived growth factor (PDGF) [486], GDF11 [487] and estrogen [488–494] promote their mobilization and homing to vascular zones. SDF-1 enhances HSC proliferation and prevents EPC apoptosis [495, 496], while exosomes and cytokines like MCP1 and VEGF enhance angiogenesis [251]. In contrast, TNF-α and other SASP factors reduce the EPC pool and impair their function [251]. The specific role of EPCs in cerebrovascular aging is further discussed in the “Circulating endothelial progenitor cells” section.

In summary, the regulation of SC function by circulating factors is a promising area of research in the understanding of aging processes. By harnessing the regenerative potential of stem cells and modulating their response to systemic signals, future therapies could target age-related diseases and improve health span. Further studies are required to fully elucidate the factors and mechanisms governing SC niches and their regenerative capacity in aging.

### Effects of circulating factors on cellular stress resilience

Cellular stress resilience, the ability of cells to respond to and recover from stressors, is a critical determinant of tissue and organismal health and aging trajectories [497–501]Aging is associated with impaired resilience to molecular stressors, including oxidative stress, metabolic stress and environmental toxicants [297–299, 382, 425, 502, 503]. Increased cellular stress can lead to the accumulation of damaged biomolecules, including lipid peroxidation, DNA damage, and protein misfolding, and dysfunctional organelles, contributing to the aging process and age-related diseases [504]. Cellular stress resilience encompasses a complex interplay of molecular pathways that collectively enable cells to respond to and recover from stressors effectively. These include antioxidant enzymes, DNA repair mechanisms, autophagy, heat shock response and other mechanisms of proteaostasis. Understanding how anti-geronic factors present in young blood impact these pathways is crucial for elucidating the mechanisms behind rejuvenation.

The nuclear factor erythroid 2-related factor 2 (Nrf2) pathway holds a pivotal role in protecting cells against oxidative stress [34, 299, 502, 505, 506]. Nrf2, a transcription factor, becomes activated in response to heightened ROS production, instigating the transcription of a broad spectrum of antioxidant and detoxification genes. Notably, aging is associated with Nrf2 dysfunction in the vasculature, which has been causally linked to increased vulnerability of aging cells to diverse stressors, heightened DNA damage, and the onset of cellular senescence [296–299, 507]. Intriguingly, there is compelling evidence that Nrf2 activity is up-regulated in rodents consuming a lifespan-extending caloric restricted diet, and this enhancement is mediated through circulating factors [508]. Importantly, vascular Nrf2 activity is regulated by anti-geronic hormones, including IGF-1 [509, 510]. Preliminary investigations also hint at the potential of exposure to young blood in bolstering Nrf2 activity, thereby up-regulating antioxidant defenses and other stress resilience mechanisms within aging cells [511]. Illustratively, a study focused on unraveling age-related variations in an organism’s resilience to hemorrhagic shock uncovered intriguing insights. It revealed that young mice exhibited remarkable survival following hemorrhagic shock, in stark contrast to their aged counterparts. Delving into the underlying mechanisms, researchers administered extracellular vesicles isolated from the plasma of young mice to aged mice subjected to hemorrhagic shock. This intervention substantially prolonged the lifespan of the aged mice, which was associated with a notable reduction in oxidative stress levels attributed to the upregulation of Nrf2-regulated target genes [511].

### Effects of circulating factors on proteostasis and autophagy

The proper functioning of all cells, tissues, and systems relies on the continuous synthesis, folding, and clearance of proteins. Proteostasis defines the delicate balance between protein production, folding, and degradation, which is crucial for cellular health and function [512–514]. This dynamic balance is maintained by a complex proteostasis network that regulates protein synthesis (via ribosomes), protein folding (via chaperones), and the clearance of misfolded, damaged, or aggregated proteins (involving chaperones, the ubiquitin–proteasome system, caspases, ADAMs, and the lysosome-autophagy system) [515]. In aging proteostasis becomes impaired, leading to the accumulation of faulty proteins and aggregates, which is particularly evident in long-lived cells such as neurons, myofibers, satellite cells, and brain endothelial cells [512–514]. This imbalance not only contributes to physiological aging but also to the onset of age-related diseases such as AD and Parkinson’s disease [516–518]. Similarly, a loss of proteostasis has been observed in aged vascular networks, which is linked to the development of cardiovascular and cerebrovascular diseases [519, 520].

Evidence supports the notion that several hallmarks of aging are driven by cell non-autonomous mechanisms, including the systemic environment [512]. However, our understanding of how the aging systemic milieu impacts proteostasis, particularly in the context of vascular aging, remains limited. As aging progresses, the proteostasis network gradually loses efficiency [521]. Heat shock proteins (HSPs), a group of chaperones, play a critical role in maintaining protein quality by folding, unfolding, and targeting faulty proteins for degradation, thus protecting cells from proteotoxicity [521]. The expression and activity of HSPs are regulated by heat shock factor 1 (HSF1), whose transcriptional activity is upregulated in response to cellular stressors, such as the accumulation of misfolded or aggregated proteins [522]. HSF1 regulates the expression of key HSPs, including HSP40, HSP70, and HSP90, which ensure proteome integrity [523]. With aging, the dysregulation of HSP function and expression becomes widespread across the body [524]. In the vasculature, aging leads to dysregulated expression of several HSPs, including HSP27, HSP60, HSP70, and small HSPs, all of which have been implicated in vascular aging and diseases [525–529]. Aging-related endothelial senescence has been associated with decreased expression of HSP72 and increased protein aggregation [530]. As discussed earlier, cellular senescence can be propagated systemically through elevated levels of circulating proinflammatory mediators, such as those linked to the SASP. These mediators act as inducers of inflammation and oxidation stress, known to impair proteostasis by oxidating and damaging proteins.

Interestingly, elevated HSP expression can also be a sign of impaired proteostasis. For instance, in the aged brain endothelium, both chronological aging and exposure to aged blood have been shown to alter the expresison HSPs. Notably, exposure to young blood normalizes the levels of this chaperone in brain endothelial cells, suggesting that young blood can help stabilize proteostasis [165, 531]. Plasma transfer experiments have further underscored the role of the systemic milieu in proteostasis regulation, showing that aged blood downregulates genes involved in ribosomal synthesis and processing in brain endothelial cells, while young blood promotes these processes [531]. The specific systemic factors responsible for these effects remain unclear. Given that HSF1 activity is regulated by SIRT1—a key player in the heat shock response and autophagy—and the documented cardioprotective effects of SIRT1, it is possible that young blood-mediated changes in HSP activity are partly driven by NAD^+^-mediated SIRT1 activation [532, 533]. Sirtuin signaling is one of four primary nutrient-sensing pathways, all of which are involved in the regulation of the unfolded protein response and heat shock mechanisms [534]. Autophagy, another critical component of proteostasis, is the process by which cells degrade, recycle, and clear damaged or misfolded proteins [535–539]. This process includes chaperone-mediated autophagy, microautophagy, and macroautophagy. Impairment of autophagy has been increasingly recognized as a common contributor to vascular aging and age-related vascular diseases [540, 541]. In endothelial cells, the decline in autophagic function is linked to oxidative stress, inflammation, and endothelial dysfunction. Strategies that enhance autophagy have shown promise in improving endothelial health and function [541–543]. As with other proteostatic mechanisms, autophagy declines with age across multiple organs. Remarkably, exposure to young blood has been shown to restore autophagic function in aged kidneys, liver, and brain, a process potentially mediated by pro-autophagy miRNAs [244, 436, 544, 545]. Cells also utilize extracellular vesicles to remove misfolded and/or aggregated proteins [546]. While this mechanism can protect the originating cell by offloading these damaged proteins, it carries a potential downside: vesicles loaded with misfolded or aggregated proteins may circulate and impair proteostasis in distant cells and tissues [547]. This suggests that while extracellular vesicles contribute to cell survival, they could also propagate proteotoxic stress systemically, thereby exacerbating dysfunction in other parts of the body. In summary, studies employing heterochronic parabiosis and blood exchange models have provided valuable insights into the systemic regulation of proteostasis during aging. However, further research is needed to fully elucidate the factors and pathways involved, which could lead to novel therapeutic approaches for restoring proteostasis and mitigating age-related diseases.

### Effects of circulating factors on nutrient sensing pathways

Nutrient-sensing pathways play a central role in regulating the aging process. These pathways, including mTOR (mammalian target of rapamycin), AMPK (AMP-activated protein kinase), Insulin/IGF-1 signaling, and sirtuins, are sensitive to changes in nutrient availability, energy status, and cellular stress. They coordinate critical cellular processes such as protein synthesis, autophagy, metabolism, and stress responses. Dysregulation of nutrient sensing is implicated in numerous age-related conditions, including metabolic syndrome, atherosclerosis, obesity, and neurodegenerative diseases [548, 549].

#### mTOR signaling

The mTOR signaling pathway is a key regulator of anabolic processes, activated in the presence of nutrients. It promotes protein synthesis, cell growth, and proliferation. However, during limited nutrient availability or upon pharmacological inhibition, mTOR signaling is downregulated, restoring catabolic processes like autophagy. Studies in lower organisms, rodents and non-human primates have demonstrated that inhibition of mTOR extends both lifespan and healthspan. Additionally, mTOR inhibition has been linked to vasculoprotective effects, including reduced endothelial senescence, improved vasodilation, enhanced blood–brain barrier integrity, and decreased oxidative stress and arterial stiffness [550–555]. While there is limited information on how age-related changes in the systemic milieu affect mTOR signaling, recent studies suggest that circulating factors in young blood may influence this pathway. For instance, analysis of transcriptomic changes in aortas from aged mice exposed to young blood revealed that RICTOR, a key subunit of the mTORC2 complex, was among the most affected upstream regulators [51]. Furthermore, young blood exposure reversed age-related transcriptomic shifts in several mTOR-related genes [51]. These findings imply that components of young blood could modulate mTOR signaling, potentially reversing age-related declines.

#### Insulin/IGF- 1 signaling (IIS)

Insulin/IGF-1 signaling (IIS) is another nutrient-sensing pathway critical for regulating metabolism and aging. IGF-1, secreted primarily by the liver, plays a central role in this pathway. Upon binding to its receptor (IGF-1R), it activates the PI3 K/Akt signaling cascade, which in turn activates protein synthesis, promotes cell proliferation, and mitochondrial biogenesis. The plasma levels of IGF-1 are highest in young organisms [556–558], suggesting that exposure to a young systemic milieu could replenish IGF-1 levels and modulate IIS signaling in aging.

IGF-1 plays a complex and multifaceted role in regulating aging processes, with its effects varying across different species and stages of life [559–566]. Early life deficiency of IGF-1 in rodents has been shown to modulate DNA repair pathways [345], reduce the incidence of cancer, and extend lifespan in certain strains [567], which aligns with the Developmental Origins of Health and Disease (DOHaD) hypothesis [568]. In this model, reduced IGF-1 signaling during critical developmental windows leads to long-term adaptations that influence aging and longevity, in part by decreasing metabolic and proliferative activity and enhancing cellular stress resistance and DNA repair [345, 567]However, the relationship between IGF-1 and longevity is not straightforward and appears to be species-specific. For instance, while GH/IGF-1-deficient Ames dwarf and Snell dwarf mice exhibit significantly extended lifespans [569], the scenario in mice with specific circulating IGF-1 deficiency [561] and in human studies [566] presents a more complex picture. Studies on mice engineered to have reduced circulating IGF-1 levels have not consistently shown the same longevity benefits observed in GH/IGF-1-deficient models [561]. In humans, individuals with genetic conditions that result in IGF-1 deficiency, such as Laron syndrome, do not experience the same lifespan extension seen in dwarf mice [566, 570]. In fact, these individuals often have shorter lifespans [566], primarily due to an increased risk of cardiovascular disease, diseases of the CNS and other metabolic complications, highlighting a crucial role of IGF-1 signaling in regulation of cardiovascular and brain aging [570]. Contrary to its role in early life deficiency, IGF-1 is a potent regulator of cardiovascular [571, 572], cerebrovascular [262], and brain [573–575] health later in life, where it exerts significant anti-aging effects. IGF-1 supports vascular function by promoting endothelial health, reducing oxidative stress, and enhancing nitric oxide production, thereby improving blood flow and reducing the risk of atherosclerosis. In the brain, IGF- 1 supports neurogenesis, protects against neuroinflammation, and helps maintain cognitive function. Numerous studies provide extensive evidence of the beneficial impact of IGF-1 on cerebrovascular aging, particularly in its ability to preserve key aspects of brain microvasculature. IGF-1 plays a vital role in maintaining capillary density within the brain, a critical factor for ensuring adequate blood supply and nutrient delivery to neurons. With aging, the reduction in capillary density contributes to impaired cognitive function, but IGF-1 has been shown to counteract this decline, promoting angiogenesis and vascular repair. In addition, IGF-1 is essential for neurovascular coupling, the process by which blood flow to active brain regions is adjusted to meet metabolic demands. Aging is often associated with a breakdown in this coupling, leading to reduced efficiency in brain function. However, IGF-1 has been found to enhance neurovascular coupling by improving endothelial function and facilitating the release of vasodilators, such as nitric oxide, which improve cerebral blood flow. IGF-1 also contributes to maintaining the integrity of the BBB. Moreover, IGF-1 plays a protective role against cerebral microhemorrhages (CMHs) [261], which are small, often unnoticed bleeds in the brain that become more common with aging and contribute to cognitive decline. IGF-1’s ability to stabilize the cerebral vasculature and reduce oxidative stress helps mitigate the occurrence of CMHs, thereby preserving brain function in aging populations [261]. Recent studies utilizing heterochronic parabiosis have further elucidated the role of IGF-1 in systemic rejuvenation, particularly in the vasculature. Our recent study analyzing transcriptomic changes in the aortas of aged heterochronic parabionts exposed to young blood revealed that IGF-1R activation may contribute to the rejuvenating effects of young blood [168]. This finding indicates that the rejuvenating effects of young blood may, at least in part, be mediated by increased IGF-1 signaling, which restores vascular function and delays vascular aging.

#### AMPK signaling

AMPK (AMP-activated protein kinase) plays a pivotal role in the regulation of energy homeostasis, acting as a metabolic “master switch” that responds to nutrient scarcity by promoting catabolic processes like autophagy and fatty acid oxidation. AMPK signaling is regulated by AMP/ATP ratio, and its activation restores energy balance by inhibiting energy-consuming processes and promoting energy-generating pathways, thereby protecting cells under conditions of stress. With aging, AMPK signaling becomes impaired, contributing to metabolic dysfunction in various tissues, including the vasculature [576, 577]. Experiments in lower organisms and rodent models have demonstrated an extended lifespan following the restoration of AMPK activation, either through genetic overexpression or pharmacological treatment with AMPK activators [578]. In the vascular system, AMPK activation has been shown to promote vasodilation, reduce inflammation, and protect against oxidative stress [579, 580]. These vasoprotective effects are critical in maintaining endothelial function and overall vascular health, which tend to decline with age.

Studies on the direct effects of the systemic environment on AMPK signaling are relatively sparse, but emerging evidence suggests that young blood may restore AMPK activity. For example, a heterochronic parabiosis study showed that the liver of aged parabionts exposed to young blood exhibited improved recovery following ischemia–reperfusion injury, a process that was mediated by increased autophagic activity, which in turn is linked to enhanced AMPK signaling [245]. This suggests that factors present in young blood may promote AMPK activation, potentially reversing some of the detrimental effects associated with aging. Another study using in vitro models of ischemia–reperfusion demonstrated that hepatocytes treated with young serum displayed increased autophagic activity mediated by enhanced AMPK signaling. This further underscores the potential role of the systemic environment in regulating nutrient-sensing pathways like AMPK, particularly in aging.

MicroRNAs have also been implicated in the regulation of AMPK signaling, and circulating miRNAs in plasma exosomes may play a role in the modulation of this pathway [581]. Several miRNAs enriched in young plasma have been shown to upregulate AMPK activity. For instance, miR- 200a, miR- 203b- 3p, miR- 187 - 3p, and miR- 378a- 3p are known to activate AMPK signaling, promoting autophagy and protecting against age-related metabolic dysfunction [581, 582]. Conversely, age-related increases in miRNAs such as miR- 34a, miR- 122, miR- 141, miR- 146a, and miR- 506 - 3p may downregulate AMPK signaling, contributing to age-related declines in autophagy and metabolic function by acting through the SIRT1-LKB1 axis [583, 584].

Other systemic factors that could potentially mediate AMPK signaling include leptin and adiponectin, two adipose tissue-derived hormones that have AMPK-specific effects [585]. Leptin is known to activate AMPK in specific tissues, while adiponectin also enhances AMPK activation. However, with aging, plasma levels of leptin tend to decline, while adiponectin levels increase, suggesting that the balance of these systemic factors could modulate AMPK signaling in the aging process [586, 587]. Although there are no direct studies focused specifically on the effects of young and aged systemic milieu on AMPK signaling in the vasculature, the evidence from heterochronic parabiosis and in vitro models suggests that young blood may help restore AMPK activity, leading to improved autophagic processes, reduced oxidative stress, and better vascular and metabolic health in aging. Further research is needed to identify the exact systemic factors responsible for the regulation of AMPK signaling and to explore their potential therapeutic applications in mitigating age-related diseases.

#### Sirtuin signaling

Sirtuins are a family of NAD + -dependent enzymes that act as cellular energy sensors, playing critical roles in metabolic regulation, DNA repair, autophagy, and inflammation control [383, 588–595]. The family of sirtuins, comprising seven members (SIRT1-SIRT7), operates in different cellular compartments, including the nucleus, cytoplasm, and mitochondria [383, 589–595]. They are key regulators of cellular homeostasis, responding to changes in energy availability and stress by modulating processes essential for health and longevity. SIRT1 and SIRT6, in particular, have been widely studied for their vasoprotective and neuroprotective properties [383, 589, 596–602]. SIRT1 activation promotes endothelial function by upregulating eNOS [603] and reducing oxidative stress [100, 229, 308, 598, 604–607], and protects the integrity of the blood–brain barrier [596, 599]. SIRT1 has also been shown to suppress pro-inflammatory signaling pathways such as NF-κB, further contributing to vascular health [608, 609]. SIRT6 is also involved in maintaining genome stability and DNA repair, functions that are crucial in preventing age-related vascular diseases [601, 602]. One of the key mechanisms by which sirtuins are regulated is through NAD + availability, which directly influences their enzymatic activity. NAD + levels decline with age, which diminishes sirtuin activity and accelerates aging processes across various tissues, including the cardiovascular and cerebrovascular systems [23, 100, 103, 610–615]. Restoring sirtuin activity, either through the administration of sirtuin-activating compounds (STACs) [119, 290, 303, 305, 616, 617] or by replenishing NAD + levels, represents a promising therapeutic strategy for rejuvenating cardiovascular and cerebromicrovascular function and protecting brain health during aging [100, 308, 605, 611, 618–620]. These interventions result in improved neurovascular coupling, enhanced endothelial function, and increased angiogenic capacity of endothelial cells [102, 103, 111, 290, 301, 308]. The rejuvenation of cerebromicrovascular health is associated with significant improvements in cognitive function in aged mice [103, 301, 621]. By restoring SIRT1 activity, these interventions help mitigate the detrimental effects of aging on the cerebral vasculature, thereby protecting the brain from age-related decline and enhancing cognitive performance. This highlights the potential of targeting SIRT1 activation and NAD + metabolism as therapeutic strategies to combat cognitive decline and cerebrovascular aging. Iportantly, heterochronic parabiosis studies have shown that aged animals exposed to young blood experience partial restoration of SIRT1-related gene expression in aortic tissues [51]. These findings suggest that circulating factors in young blood may promote sirtuin activity and confer anti-aging effects. Further research is needed to elucidate the specific mechanisms by which parabiosis and exposure to young systemic milieu regulate sirtuin pathways, particularly in cerebrovascular endothelial cells and the brain, to better understand their contributions to neurovascular and cognitive health.

One of the circulating factors identified in young blood that could enhance sirtuin activity is extracellular vesicles containing extracellular nicotinamide phosphoribosyltransferase (eNAMPT), an enzyme critical for NAD + biosynthesis [622]. Studies have demonstrated that plasma levels of eNAMPT decline with age, correlating with reduced lifespan and function [622]. Recent studies demonstrate that exercise stimulates the release of EVs containing eNAMPT, which enhance NAD + levels and SIRT1 activity in recipient cells, highlighting a potential exercise-mediated mechanism to counteract age-related NAD + decline and promote sirtuin activation [623]. Based on these observations, it has been proposed that EVs enriched with eNAMPT derived from young blood may hold potential for enhancing cerebrovascular function in aged parabionts, offering a novel mechanism for mitigating age-related vascular decline [624].

Circulating miRNAs have also emerged as potential regulators of sirtuin activity [625]. Several miRNAs that increase with aging, such as miR- 9, miR- 22, and miR- 34, miR- 33, miR- 146, and miR- 199, have been shown to directly downregulate SIRT1 expression [625, 626]. Conversely, other miRNAs found in young plasma may upregulate SIRT1, promoting its anti-aging effects.

Further evidence comes from caloric restriction (CR) studies, which have shown that serum from calorie-restricted animals enhances SIRT1 activity [228, 230, 232, 248], improves mitochondrial function and confer anti-oxidatkve and pro-angiogenic effects in cultured cells [229, 233]. These findings suggest that systemic factors influenced by anti-aging dietary interventions may regulate sirtuin signaling, offering a pathway for therapeutic interventions to delay cerebrovascular and brain aging. Sirtuins are also linked to other nutrient-sensing pathways. For instance, SIRT1 interacts with AMPK, forming a positive feedback loop that enhances autophagy and metabolic balance [593, 627–629]. The activation of SIRT1 also promotes mTOR inhibition, favoring catabolic processes like autophagy over anabolic processes, which is crucial for maintaining cellular homeostasis during aging [593].

### Effects of circulating factors on the extracellular matrix

The extracellular matrix (ECM) is a vital, acellular component of every tissue and organ, providing not only structural support but also playing key roles in biochemical signaling, cellular differentiation, proliferation, and intercellular communication [630]. The ECM undergoes constant remodeling, balancing between biosynthesis and degradation processes [631]. With advancing age, ECM homeostasis deteriorates in a tissue-specific manner, contributing to organismal aging [630, 632–641]. This age-related decline involves increased glycation, rendering ECM components resistant to enzymatic digestion and resulting in pathological tissue stiffening [642]. Additionally, aging induces excessive fragmentation of ECM components, which disrupts intercellular communication, impairs mechanical stability, and increases the risk of fibrosis due to uncontrolled protease activation and inflammation [630, 632–641].

Within vascular networks, the ECM provides structural support, elasticity, and mechanical integrity to the vascular wall, while concurrently regulating the communication, differentiation, and proliferation of resident endothelial and vascular smooth muscle cells [635, 640]. The dysfunction of vascular ECM during aging is a significant contributor to the decline in elasticity and functionality of blood vessels, leading to vascular stiffening and impaired vascular function [633, 643]. This decline in vascular stability is exacerbated by persistent mechanical stress and the chronic low-grade inflammation that accompanies aging [644]. As previously discussed, aging is associated with chronic inflammation and cellular senescence, both of which promote the secretion of proinflammatory cytokines, chemokines, ROS, and matrix metalloproteinases (MMPs), contributing to the degradation of ECM components [119, 633, 645–650]. MMPs are a family of 23 endopeptidases capable of degrading various ECM components [651]. Their expression is regulated by proinflammatory cytokines (e.g., IL- 1β, TNF-α) and tissue inhibitors of matrix metalloproteinases (TIMPs), the latter playing a crucial role in controlling MMP activity [644, 651]. Both MMPs and TIMPs are integral parts of the senescence-associated secretory phenotype (SASP), with specific MMPs (e.g., MMP- 1, MMP- 3, MMP- 10, MMP- 12, MMP- 13, and MMP- 14) and TIMPs (e.g., TIMP- 1 and TIMP- 2) showing dysregulated activity during aging [422]. Research has revealed significant changes in the plasma levels of MMPs and TIMPs across the lifespan, with several studies demonstrating correlations between these changes and the pathogenesis of cardiovascular and cerebrovascular diseases [652–656]. The role of MMPs and TIMPs in cerebrovascular disease pathogenesis is discussed further in the “Tissue inhibitor of metalloproteinases 2 (TIMP2)” section.

Evidence suggests that many of these age-related ECM alterations are governed by circulating factors. The renin–angiotensin–aldosterone system (RAAS), which is up-regulated during aging, plays a crucial role in mediating ECM changes [16, 119, 131, 645, 657]. The decline in circulating IGF- 1 levels with aging also contributes to ECM dysregulation, as IGF- 1 is known to support ECM integrity and vascular health [131, 658]. Additionally, collagen synthesis in the vascular wall becomes dysregulated with age, likely due to the increased paracrine action of transforming growth factor-β (TGF-β), which promotes vascular fibrosis and arterial stiffening [16]. Heterochronic parabiosis studies add to the growing evidence that age-related changes in ECM and vascular stability are mediated by cell non-autonomous mechanisms. For example, in a heterochronic parabiosis study, increased ROS levels were detected in the walls of aortas from young heterochronic parabionts exposed to aged blood [51]. Transcriptomic analysis revealed that aged-blood-induced vascular remodeling in young aortas is mediated through the inhibition of serum response factor (SRF)-, IGF- 1-, and VEGF-related signaling pathways [51, 659]. Beyond the vasculature, ECM aging has been shown to be modulated by systemic factors in other organs. For instance, in aged cartilage, exposure to young blood improved chondrocyte proliferation and normalized ECM composition (increased collagen II, decreased collagen X, and reduced MMP- 13 expression) [660]. In contrast, exposure to aged systemic milieu impaired chondrocyte proliferation, though without significant effects on ECM composition [661].

Moreover, miRNAs play an important role in regulating ECM-related genes during aging. For instance, the analysis of non-coding RNAs (ncRNAs) in plasma, liver, kidneys, lungs, and adipose tissue revealed that miR- 29 - 3c is significantly restored upon exposure to young blood [388]. This miRNA regulates the expression of numerous ECM-related genes (e.g., Eln, Col1a1, Col1a2, Col3a1, and Adam12) [388]. Furthermore, heterochronic parabiosis and blood exchange studies have shown that exposure to aged blood is associated with increased expression of MMPs, TIMPs, and their regulators (e.g., TNF-α, IL- 1β) [170, 181, 429].

Collectively, these studies suggest that modulating ECM aging through systemic interventions holds significant therapeutic potential. The ability of systemic factors to influence ECM homeostasis opens up promising avenues for combating age-related tissue deterioration. However, further studies are needed to specifically focus on the effects of young and aged systemic milieus on ECM components in both vascular and organismal aging. Understanding the interplay between aging processes, ECM components, MMPs, TIMPs, and changes in the systemic environment will be key to developing novel interventions that target age-related degeneration in tissues and organs.

## Role of systemic factors present in blood in the pathogenesis of age-related vascular and brain diseases

Systemic factors present in the blood, such as hormones, proinflammatory mediators, growth factors, metabolites, and circulating cells, are central to the development and progression of a broad spectrum of age-related vascular and brain diseases. As we age, the composition of these circulating factors changes significantly, leading to increased inflammation, oxidative stress, and cellular dysfunction. These alterations have profound effects on critical cellular components such as endothelial and smooth muscle cells, the extracellular matrix, and neural structures, driving the onset of conditions like atherosclerosis, vascular cognitive impairment, and neurodegenerative diseases such as AD and Parkinson’s disease. In this section, we will examine the impact of systemic factors on these disease processes, highlighting how both the aged and young systemic environments influence vascular and brain aging, either exacerbating or potentially ameliorating pathological changes.

### Atherosclerosis

Atherosclerosis is an age-related vascular disease characterized by the formation of atheromatous plaques within the walls of large arteries [662]. Atherosclerosis of cerebral arteries contributes to the pathogenesis of stroke and VCID [5, 663]. Atherosclerotic plaques, which consist of lipid deposits, inflammatory cells, and fibrous material, lead to narrowing and stiffening of the blood vessels, restricting blood flow and increasing the risk of cardiovascular events, such as strokes [662]. The pathogenesis of atherosclerosis involves several key steps, including endothelial cell activation, infiltration of low-density lipoproteins (LDLs) into the arterial wall, local inflammation, and the formation of fatty streaks and fibrous plaques [662]. Over time, these plaques may rupture, leading to thrombus formation that can block the vessel, resulting in ischemia or infarction in the affected tissues. Endothelial cells, which line the inner surface of blood vessels, are particularly susceptible to age-related dysfunction as they are constantly exposed to circulating blood and its constituents. This makes them highly vulnerable to pro-inflammatory and damaging factors that accumulate in aged plasma. Early parabiosis studies demonstrated that systemic factors contribute to arterial lesion formation [664]. Various pro-atherogenic factors enriched in aged blood have since been identified, including modified lipoproteins such as oxidized LDL (oxLDL), TNFα, IL- 1, fibroblast growth factor 23 (FGF23), and advanced glycation end products (AGEs) [665–670]. These factors promote inflammation, endothelial dysfunction, and plaque development, accelerating the progression of atherosclerosis. Transcriptomic analyses of aortas from young heterochronic parabionts exposed to aged blood have revealed the activation of biological pathways associated with atherosclerosis [241]. These findings provide strong evidence for the pro-atherogenic effects of the aged systemic milieu, highlighting its role in promoting vascular pathology through the modulation of gene expression and cellular processes in vascular tissues.

#### Adipokines and obesity-related factors

Adipose tissue functions as an endocrine organ, releasing a diverse array of signaling molecules collectively referred to as adipokines [671, 672]. These include leptin, adiponectin, resistin, TNF-α, plasminogen activator inhibitor- 1 (PAI- 1), fatty acids, hormones, and growth factors [671, 673]. The profile and balance of these adipokines undergo significant shifts with aging and metabolic disorders such as obesity and type 2 diabetes, driving systemic inflammation and promoting the development of atherosclerosis [674]. For instance, leptin, which regulates energy homeostasis, exhibits pro-inflammatory and pro-atherosclerotic properties at elevated levels seen in obesity. Similarly, resistin has been implicated in promoting endothelial dysfunction, vascular inflammation, and the progression of atherosclerosis. In contrast, adiponectin, another key adipokine, plays an anti-inflammatory and anti-atherosclerotic role by enhancing endothelial function, reducing oxidative stress, and inhibiting the formation of atherosclerotic plaques [674, 675]. However, its protective effects are context-dependent; paradoxically, high adiponectin levels in elderly individuals have been associated with frailty and insulin resistance. Additionally, adiponectin levels are markedly reduced in individuals with metabolic syndrome, type 2 diabetes, and coronary artery disease, conditions strongly linked to atherosclerosis. Obesity amplifies the secretion of pro-inflammatory adipokines such as TNF-α, PAI- 1, and retinol-binding protein 4, while impairing the production of protective factors like adiponectin [676]. This imbalance contributes to chronic low-grade inflammation, endothelial activation, and vascular remodeling, key processes that underpin the pathogenesis of atherosclerosis. These findings underscore the central role of adipokines as mediators of the complex interplay between metabolic health, inflammation, and vascular aging, highlighting the importance of addressing obesity and its metabolic sequelae to mitigate cardiovascular risk.

#### CHIP and inflammatory mediators

Clonal hematopoiesis of indeterminate potential (CHIP) has emerged as an important age-related condition [677] that contributes to the development of atherosclerosis [678–683]. CHIP occurs when certain hematopoietic stem cells acquire mutations that lead to clonal expansion of blood cells without overt hematologic malignancies [677]. These mutated cells, particularly in older adults, produce increased levels of inflammatory cytokines, including IL- 6 and TNF-α, which are known to exacerbate atherosclerosis [679, 681, 684, 685]. CHIP mutations in genes such as TET2 and DNMT3 A have been associated with a higher risk of cardiovascular events, largely due to their contribution to chronic inflammation in the vascular system [678, 686, 687]. The inflammatory mediators secreted by these mutant cells such as IL- 6 and TNF-α contribute to endothelial dysfunction, plaque instability, and progression of atherosclerosis, making CHIP a critical factor in the disease’s pathogenesis [679–683, 688]. This connection between CHIP and vascular aging underscores its significance as a driver of cardiovascular events in older adults. Furthermore, CHIP represents a distinct link between age-related clonal dynamics in the hematopoietic system and the systemic inflammatory milieu. While the role of CHIP in promoting vascular inflammation is well-documented, its potential contribution to the transposition of aging phenotypes through parabiosis models remains unexplored. Future research is needed to investigate whether CHIP-associated cells or their secreted factors play a role in the systemic effects of aged blood. Studies could also explore whether targeting CHIP-related inflammatory pathways might mitigate the detrimental effects of aged blood in parabiosis experiments or provide therapeutic avenues for age-related diseases. Understanding the intersection of CHIP, circulating factors, and the transposition of aging phenotypes could shed new light on the cellular contributors to the systemic pro-geronic environment.

#### Protective systemic factors against atherosclerosis

While many systemic factors drive atherogenesis, protective components within the systemic milieu play a critical role in counteracting vascular pathology. One such factor is adiponectin, which exerts potent anti-inflammatory and anti-atherosclerotic effects by enhancing lipid metabolism, reducing oxidative stress, and improving endothelial function [676, 689, 690]. Additionally, several factors enriched in young blood, including IGF- 1, GDF- 11, fibroblast growth factor 21 (FGF21), lipocalin- 2, and soluble klotho, have been shown to protect against atherosclerotic plaque formation [691–694]. These factors act through diverse mechanisms, such as promoting endothelial cell repair, reducing vascular inflammation, and improving metabolic processes, highlighting their potential as therapeutic targets for preventing or mitigating atherosclerosis.

##### Role of IGF- 1 in atheroprotection

IGF- 1 plays a pivotal role in maintaining vascular health and preventing the progression of atherosclerosis [695–699]. Epidemiological studies show an inverse relationship between circulating IGF- 1 levels and cardiovascular risk [700–708]. Lower levels of IGF- 1 have been associated with increased incidence of atherosclerosis, coronary artery disease, and stroke [709, 710]. For example, in a study of elderly individuals, those with lower serum IGF- 1 levels were found to have higher rates of carotid artery plaques and increased arterial stiffness, both of which are markers of atherosclerotic burden [711]. Conversely, higher IGF- 1 levels have been linked to improved vascular function, plaque stability, and a reduced risk of cardiovascular events [712, 713]. Insights from parabiosis studies also highlight IGF- 1 as a potential contributor to atheroprotective effects of young blood [51, 168]. Transcriptomic analysis of aged aortas exposed to young blood has shown significant upregulation of IGF- 1R signaling pathways, supporting the rejuvenating effects of IGF- 1 in the young systemic milieu on vascular tissues [168]. These findings align with IGF- 1’s well-established vasoprotective functions, including preserving endothelial function, mitigating oxidative stress, and enhancing vascular repair [131, 510, 699, 714–716].

Endothelial dysfunction is an early and critical event in atherosclerosis. IGF- 1 enhances endothelial nitric oxide (NO) production by attenuating oxidative stress and upregulating endothelial nitric oxide synthase (eNOS) activity [699, 717]. NO is a key vasodilator that inhibits platelet aggregation, reduces leukocyte adhesion, and promotes smooth muscle relaxation, collectively maintaining vascular tone and reducing plaque formation. Additionally, IGF- 1 protects endothelial cells from apoptosis under oxidative stress conditions, preserving vascular integrity [718].

Chronic inflammation plays a central role in the pathogenesis of atherosclerosis. IGF- 1 exerts anti-inflammatory effects by downregulating proinflammatory cytokines such as interleukin- 6 and TNFα [699]. These cytokines contribute to the recruitment of inflammatory cells to the arterial wall, thereby accelerating the atherogenic process. By mitigating inflammation, IGF- 1 helps protect against the formation of atherosclerotic plaques and limits vascular damage [699].

Vascular smooth muscle cells are involved in the structural integrity of blood vessels. During atherosclerosis, vascular smooth muscle cells (VSMCs) proliferate and migrate to form the fibrous cap of plaques. However, excessive VSMC apoptosis can weaken the fibrous cap, making it more prone to rupture. IGF- 1 promotes the survival of VSMCs by activating the PI3 K/Akt pathway, which inhibits pro-apoptotic signaling [719]. By enhancing VSMC survival and reducing plaque instability, IGF- 1 contributes to atheroprotection by preventing plaque rupture, a key event leading to thrombosis and cardiovascular events [696].

Oxidative stress, characterized by the excessive production of ROS, plays a significant role in endothelial dysfunction and atherosclerosis. IGF- 1 mitigates oxidative stress by enhancing antioxidant defenses in vascular cells [509, 699, 717]. It stimulates the expression of antioxidant enzymes, including superoxide dismutase and glutathione peroxidase, which neutralize ROS and prevent the oxidative damage that contributes to endothelial dysfunction and inflammation [717].

IGF- 1 promotes vascular repair by stimulating the migration and proliferation of EPCs, which are involved in the repair of damaged blood vessels [720]. In aging and atherosclerosis, the number and function of EPCs decline, leading to impaired vascular regeneration. IGF- 1 has been shown to enhance the recruitment and function of EPCs, thereby promoting vascular repair and improving blood flow. This regenerative capacity of IGF- 1 is critical for maintaining vascular health and preventing the progression of atherosclerosis [699, 720].

### Cerebromicrovascular dysfunction, CSVD, and microvascular contributions to cognitive decline

The brain relies on an intricate cerebrovascular system to ensure adequate blood flow, nutrient delivery, and waste clearance, all of which are vital for maintaining neural homeostasis and function [4, 17, 147, 721]. Central to this system is the dense network of microvessels that permeate the brain, supporting neuronal activity and protecting against injury [722]. However, aging introduces structural and functional impairments to this microvascular network, including endothelial dysfunction [17], neurovascular uncoupling [17, 147, 723], BBB disruption [253, 254], microvascular rarefaction (a reduction in capillary density) [116], and increased fragility leading to microhemorrhages [133]. These age-related cerebrovascular changes collectively result in diminished brain perfusion, chronic neuroinflammation, and heightened vulnerability to VCID. Among these changes, the development of cerebral small vessel disease (CSVD) represents a critical pathology [255, 724–727].

Over the past two decades, research has increasingly underscored the pivotal role of the systemic milieu in driving age-related changes in the cerebral microcirculation [262]. Among the most compelling evidence comes from heterochronic parabiosis experiments, which demonstrate that young systemic factors can rejuvenate cerebrovascular health [163, 164]. Exposure to young blood has been shown to enhance endothelial function, restore BBB integrity, increase microvascular density, and mitigate neuroinflammation [163, 164]. These findings highlight the potential of systemic interventions to reverse key aspects of cerebromicrovascular aging. Transcriptomic analyses further reveal that cerebromicrovascular endothelial cells are particularly responsive to the rejuvenating effects of young systemic factors [165]. Conversely, exposure to aged blood accelerates cerebromicrovascular and neurovascular decline, underscoring the deleterious impact of circulating pro-geronic factors [163, 164].

In the following sections, we explore the mechanisms and pathophysiological contributions of cerebrovascular dysfunction, including CSVD, neurovascular uncoupling, BBB disruption, microvascular rarefaction and cerebral microhemorrhages, as well as the role of systemic factors in these processes. These insights underscore the potential of targeting the systemic milieu to combat cognitive decline and age-related brain diseases.

#### CSVD

CSVD encompasses a range of age-related pathologies affecting the small arteries, arterioles, capillaries, and venules in the brain [721, 725–727]. The radiological hallmarks of CSVD include white matter hyperintensities (WMHs), CMHs (also known as cerebral microbleeds), lacunar infarcts, and enlarged perivascular spaces (PVS) [721, 728–730]. These manifestations of CSVD are indicative of significant cerebrovascular dysfunction and contribute to cognitive decline, gait disturbances, and an increased risk of both ischemic and hemorrhagic strokes. WMHs, visible on MRI as areas of increased signal intensity, result from white matter damage and are linked to BBB disruption, neuroinflammation, and impaired cerebral blood flow and oxygenation [731]. The resulting chronic ischemia and inflammation in white matter lead to cognitive decline and motor dysfunction. BBB dysfunction in CSVD increases its permeability, allowing neurotoxic molecules from the systemic circulation to enter the brain, exacerbating neuroinflammation and damaging neural structures [250]. CMHs are small, focal hemorrhages caused by the rupture of fragile cerebral microvessels [133]. These microbleeds are linked to cognitive impairment and an increased risk of hemorrhagic stroke. Lacunar infarcts, caused by the occlusion of small penetrating arteries, lead to localized brain tissue death. Though often asymptomatic in early stages, they contribute to cumulative cognitive impairment, motor dysfunction, and increased risk for vascular dementia [732, 733]. Enlarged PVS, or Virchow-Robin spaces, are small fluid-filled cavities surrounding blood vessels in the brain [734]. Enlarged PVS are thought to be markers of vascular injury and have been linked to cognitive decline. These cerebrovascular pathologies are also frequently associated with age-related microvascular rarefaction, endothelial dysfunction and impaired neurovascular coupling responses. The reduced cerebromicrovascular density in the aging brain combined with the microvascular functional impairment contribute to decreased brain perfusion and an increased vulnerability to ischemia and neurodegeneration [17]. As a result, the risk of VCID as well as neurodegenerative conditions such as AD is markedly increased in individuals with CSVD.

Systemic factors are thought to play an important role in the pathogenesis of CSVD. Circulating pro-inflammatory mediators, such as cytokines, can exacerbate endothelial dysfunction, contributing to impaired neurovascular coupling, chronic inflammation, and increased BBB permeability [735, 736]. Furthermore, systemic factors such as GDF- 11 [60], IGF- 1 [97, 98, 109, 258, 264, 556, 658], and others have shown protective effects against CSVD, suggesting that modulating the systemic milieu could be a potential therapeutic strategy for slowing CSVD progression and preventing cognitive decline.

#### Endothelial dysfunction and neurovascular uncoupling

**Fig. 7:**
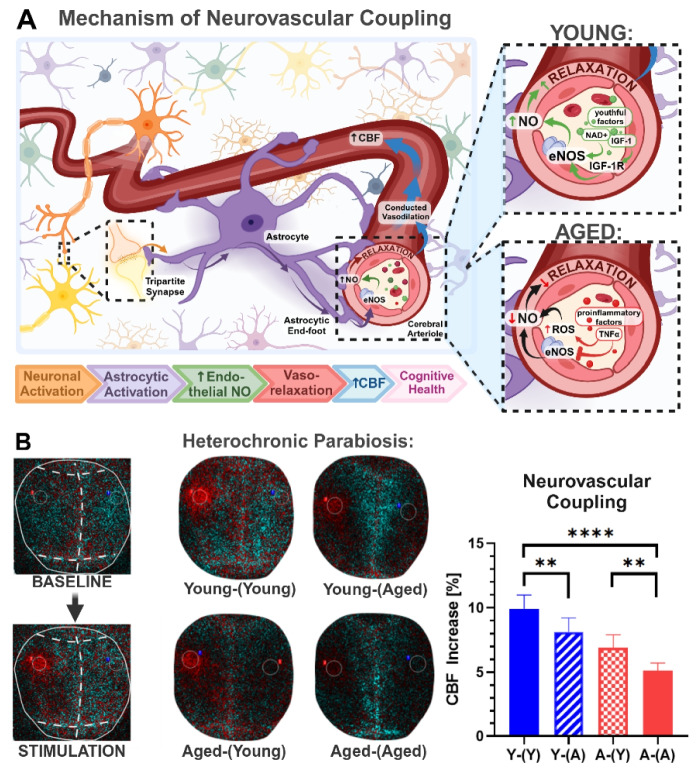
Mechanisms of neurovascular coupling and the impact of aging and young systemic milieu.** A** Schematic representation of the neurovascular unit and neurovascular coupling (NVC) mechanisms. Neuronal activation stimulates astrocytes and endothelial cells, leading to increased production of nitric oxide (NO) by endothelial nitric oxide synthase (eNOS). This promotes vasodilation, enhances regional cerebral blood flow (CBF), and supports neuronal metabolic demands by delivering oxygen and nutrients while removing metabolic byproducts. The panels on the right compare the effects of young and aged systemic milieus. In young conditions, circulating anti-geronic factors (e.g., IGF- 1, NAD^+^) enhance eNOS activity, promote NO production, activate sirtuins, reduce oxidative stress, and improve vasodilation. Conversely, the aged systemic milieu is enriched with pro-geronic factors (e.g., TNFα), which impair eNOS function, increase reactive oxygen species (ROS) production, decrease NO bioavailability, and compromise vasodilation. **B** Experimental data from heterochronic parabiosis studies showing the influence of systemic milieus on NVC. NVC responses were assessed using laser speckle contrast imaging (LSCI) over the somatosensory cortex during contralateral whisker stimulation. Representative pseudocolor images depict baseline and stimulation-induced CBF changes, with differences highlighted in red within the whisker barrel cortex. Quantified changes in CBF, representing NVC responses, are displayed in bar graphs. Results show that exposure to young blood rejuvenates NVC responses in aged heterochronic parabionts, while exposure to old blood impairs NVC in young parabionts, mimicking aging-related phenotypes. Data are presented as mean ± S.D., analyzed using one-way ANOVA with post hoc Tukey’s tests (**p < 0.01, ****p < 0.0001, *n* = 8 per group). The representative images and quantitative data shown here are reproduced with permission from the publisher [163]

Neurovascular coupling (NVC) is the process by which active brain regions receive an adequate supply of blood, oxygen, and nutrients to meet their heightened metabolic demands [17, 147]. This process is facilitated by the neurovascular unit and it operates through the endothelium-dependent dilation of arterioles [103, 290, 621, 737–740]. This ensures that cerebral blood flow (CBF) increases locally in response to neuronal activation (Fig. 7) [149]. In aging, however, NVC responses become increasingly impaired, a phenomenon known as neurovascular uncoupling, which is a hallmark of cerebrovascular aging [103, 105, 107, 163, 556, 621]. Neurovascular uncoupling in aged individuals has been associated with diminished cognitive function and increased risk of VCID [106, 112, 147, 149, 741–744]. Endothelial dysfunction plays a central role in the age-related decline of NVC [17, 103, 107, 418, 616, 621].

Heterochronic parabiosis has provided compelling evidence for the influence of systemic factors on cerebrovascular aging. Studies have demonstrated that exposing aged mice to the young systemic milieu through parabiosis can rejuvenate endothelial function and improve NVC responses [51, 178]. For example, recent research from our group showed that aged heterochronic parabionts exposed to young blood for six weeks exhibited significantly improved NVC responses compared to aged isochronic parabionts [178]. This suggests that circulating factors in young blood can mitigate the neurovascular dysfunction associated with aging. Conversely, young heterochronic parabionts exposed to aged blood developed impaired NVC responses, indicating that the aged systemic environment accelerates neurovascular aging (Fig. 7) [178].

Several systemic factors have been implicated in the rejuvenation of NVC. Insulin-like growth factor 1 is one such factor that plays a protective role in endothelial and neurovascular health [98, 109, 556, 739]. Age-related declines in circulating IGF- 1 have been linked to endothelial dysfunction, impaired NVC, and cognitive deficits [109, 556]. Previous studies have shown that IGF- 1 signaling in endothelial cells, particularly through IGF- 1 receptor (IGF- 1R), is crucial for maintaining proper NVC responses [739]. Experimental evidence suggests that genetically induced circulating IGF- 1 deficiency or genetic disruption of IGF- 1R signaling in endothelial cells can significantly impair NVC and endothelial function in young mice, mimicking the aging phenotype [97, 98, 109, 556]. Additionally, other circulating factors, including those that improve mitochondrial function, reduce oxidative stress and restore cellular NAD + levels [100, 103, 105, 107] could be implicated in young blood mediated rejuvenation. Conversely, systemic factors known for their detrimental effects on endothelial function, such as proinflammatory mediators, including TNFα, might be responsible for aged blood-induced impairments in NVC [745]. These findings highlight the plasticity of neurovascular function and suggest that both pro-aging and anti-aging circulating factors play critical roles in regulating neurovascular health. Further studies are needed to identify the specific circulating factors that mediate these effects and to develop targeted therapeutic strategies for improving neurovascular health and preventing age-related cognitive decline.

#### Blood–brain barrier disruption and neuroinflammation

Aging is associated with significant changes in the integrity of the BBB, a critical component of the neurovascular unit [114, 249, 250, 253, 254, 746, 747]. The BBB is primarily formed by highly specialized brain endothelial cells, which maintain tight junctions, selective transport mechanisms, and efflux pumps that regulate the passage of substances between the bloodstream and brain tissue. This protective barrier is essential for maintaining the homeostasis of the neural environment and preventing harmful molecules from entering the brain. However, with age, the BBB becomes increasingly compromised, leading to increased permeability and contributing to neuroinflammation, cellular damage, including WMHs, VCID and neurodegenerative diseases, including AD [148, 249, 250, 253, 254, 747, 748].

**Fig. 8:**
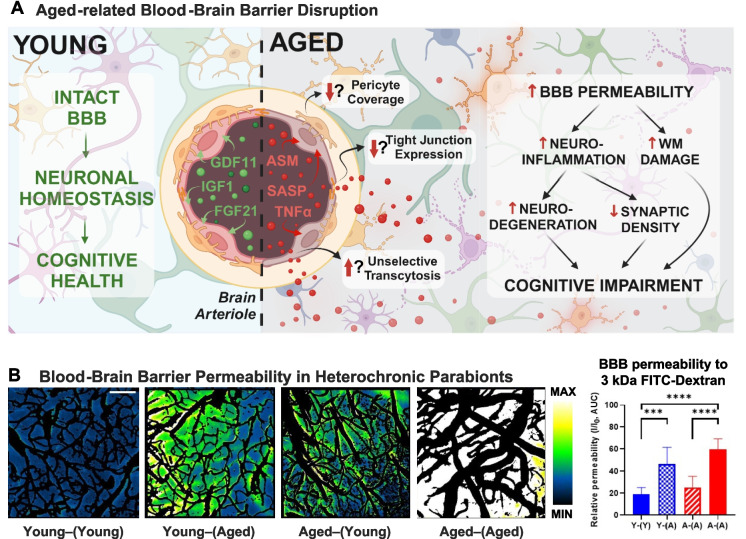
Age-related blood–brain barrier disruption and the effects of systemic milieu. **A** Schematic representation of the BBB under young and aged systemic conditions, illustrating age-related mechanisms of BBB dysfunction. In young conditions, the BBB is intact, supported by tight junction integrity and selective transcytosis, which maintain neuronal homeostasis and cognitive health. Circulating anti-geronic factors, such as GDF11, IGF- 1, and FGF21, play critical roles in maintaining BBB function. In aging, the BBB becomes increasingly permeable due to reduced tight junction expression and enhanced unselective transcytosis. The aged systemic milieu, enriched with pro-geronic factors (e.g., SASP factors), exacerbates BBB disruption, leading to neuroinflammation, white matter (WM) damage, reduced synaptic density, neurodegeneration, and cognitive decline. **B** Experimental evidence demonstrating BBB permeability changes in heterochronic parabionts, assessed by the permeability of 3 kDa FITC-dextran. Representative two-photon microscopy images depict tracer extravasation in the brain microvasculature. Minimal extravasation is observed in young isochronic parabionts, shown by the predominantly dark blue extravascular space. Isochronic aged parabionts display significant BBB permeability, highlighted by increased yellow and white fluorescence in the brain parenchyma, indicating FITC-dextran leakage. Heterochronic aged parabionts exhibit reduced tracer extravasation, suggesting that young blood restores BBB integrity. Conversely, aged blood exposure increases BBB permeability in heterochronic young parabionts, as evidenced by heightened tracer leakage. Quantitative analysis of FITC-dextran extravasation is presented in the bar graph, with data shown as mean ± SD (*n* = 7–9 per group). Statistical significance was analyzed using one-way ANOVA with Sidak’s post hoc tests (***p < 0.001, ****p < 0.0001). The representative images and quantitative data are reproduced with permission from the publisher [164]. Scale bar, 100 µm

In aging, several factors contribute to BBB disruption (Fig. 8). These include endothelial dysfunction, cellular senescence and the accumulation of proinflammatory mediators [249, 250, 255, 256, 749, 750]. The loss of tight junction integrity in the aging brain allows harmful molecules such as cytokines, inflammatory mediators and other neurotoxic substances to infiltrate the brain parenchyma, activating microglia and astrocytes, which in turn exacerbate neuroinflammatory processes [249, 250, 746]. This cascade of events not only leads to neuronal damage but also plays a significant role in cognitive decline and the development of VCID and AD [249, 250]. The mechanisms by which the systemic milieu impacts BBB integrity have become an important area of research.

Recent studies using heterochronic parabiosis models have provided insights into the role of systemic factors in regulating BBB integrity. Our research has shown that exposure to a young systemic environment can reverse BBB disruption in aged animals [164]. Through advanced imaging techniques such as in vivo two-photon microscopy, we demonstrated that aged mice exposed to young blood exhibited reduced BBB permeability across a range of molecular sizes (3 to 40 kDa) [164]. In contrast, young mice exposed to aged blood rapidly developed increased BBB permeability, underscoring the detrimental effects of progeronic factors present in aged circulation (Fig. 8) [164]. The mechanisms underlying these changes are not yet fully understood, but evidence suggests that certain circulating factors in young blood play a protective role in maintaining BBB integrity. For example, factors such as IGF- 1 [258] have been implicated in enhancing endothelial cell function and preserving tight junction proteins, which are crucial for maintaining BBB integrity. IGF- 1 was also shown to mitigate neuroinflammation [751]. Conversely, the aged systemic milieu contains proinflammatory mediators that contribute to BBB breakdown [164]. Factors such as TNF-α, interleukins, and other SASP factors have been shown to weaken the BBB by disrupting tight junctions and promoting transcytosis [260, 752, 753]. Additionally, recent research identified acid sphingomyelinase (ASM) as a potential mediator of BBB permeability in aging [754]. ASM levels increase with age, leading to enhanced caveolae-mediated transcytosis, which facilitates the transport of harmful molecules into the brain and contributes to neuroinflammation.

Our studies highlight the importance of systemic factors in mitigating neuroinflammation [164]. Preclinical studies by others also have shown that interventions using young plasma, bone marrow, or umbilical cord blood can decrease neuroinflammatory pathways, reducing microglial activation [218, 755–758]. Two recent studies have provided additional evidence for the role of systemic milieu in mitigating neuroinflammation. The study by Mehdipour et al. demonstrated that plasma dilution in aged mice improved cognitive function and attenuated neuroinflammation [759]. These authors revealed that the dilution of proinflammatory and progeronic factors in aged plasma through neutral blood exchange reduced the activation of microglia, decreased levels of inflammatory cytokines, and restored cognitive abilities in aged mice [206, 208]. This observation supports the hypothesis that circulating proinflammatory factors contribute significantly to BBB dysfunction and that systemic interventions can reverse these effects. In another study, plasma from healthy human donors was shown to protect BBB integrity in a mouse model of cerebral ischemia, primarily through the action of fibroblast growth factor 21. FGF21, a systemic factor present in plasma, was found to enhance the recovery of BBB function after ischemic injury by preserving tight junction proteins and reducing endothelial cell apoptosis. The administration of healthy donor plasma improved recovery from ischemic injury and protected against BBB disruption, highlighting FGF21 as a critical factor in promoting BBB health and mitigating ischemia-induced damage. This study points to the potential therapeutic role of plasma-derived factors like FGF21 in preventing age-related BBB disruption and neuroinflammation [760].

#### Cerebromicrovascular rarefaction, hypoperfusion and ischemic injury

**Fig. 9:**
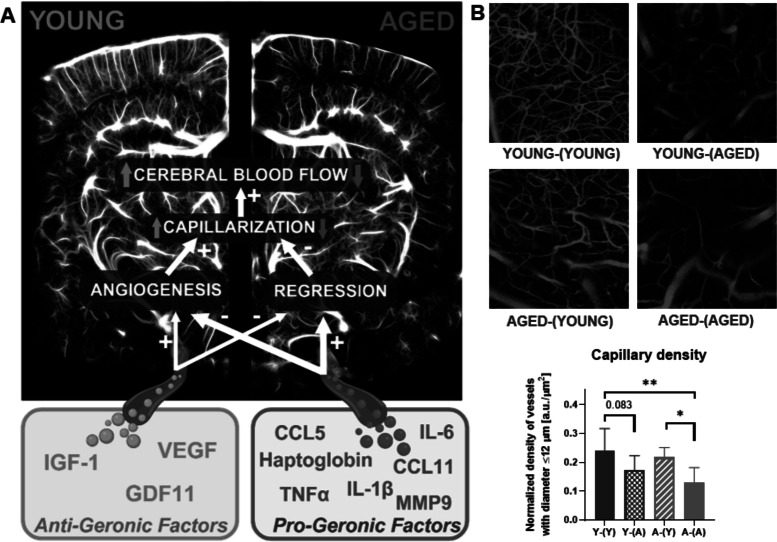
Role of circulating factors in microvascular rarefaction. **A** Microvascular rarefaction, characterized by a reduction in capillary density, is a hallmark of cerebrovascular aging and is driven by impaired angiogenesis and increased vascular regression. Systemic anti-geronic factors, such as IGF- 1, GDF11, and VEGF, positively regulate angiogenesis and inhibit vascular regression, preserving capillary density. Conversely, the aged systemic milieu contains elevated levels of pro-geronic factors, including proinflammatory cytokines, which drive vascular regression and contribute to microvascular rarefaction. These changes compromise cerebral blood flow and capillarization, exacerbating age-related cognitive decline. **B** Representative images from two-photon microscopy show cortical blood vessels labeled with WGA-AF594 (red) from each experimental group, including isochronic young (Y-Y), isochronic aged (A-A), and heterochronic parabionts (Y-A, A-Y). These images highlight the effects of systemic milieu on microvascular health. Isochronic aged parabionts exhibited the lowest capillary density, significantly reduced compared to isochronic young parabionts (*p* < 0.01). In aged heterochronic parabionts, exposure to young blood preserved capillary density, demonstrating the rejuvenating effects of youthful systemic factors. In contrast, young heterochronic parabionts exposed to aged blood showed a significant reduction in capillary density, mirroring the vascular regression observed in aging. The bar graph quantifies capillary density differences across groups, showing mean ± SD (*n* = 7–9 per group, one-way ANOVA with post hoc Sidak’s tests; *p < 0.05, **p < 0.01). These findings provide evidence for the critical role of circulating factors in microvascular rarefaction, with young systemic milieu enhancing vascularization and aged systemic milieu accelerating vascular regression. Abbreviations: IGF- 1, insulin-like growth factor 1; GDF11, growth differentiation factor 11; VEGF, vascular endothelial growth factor; IL- 6, interleukin 6; TNFα, tumor necrosis factor-alpha; MMPs, matrix metalloproteinases. Representative images and quantitative data are reproduced with permission from the original authors and publisher [164]

With aging, brain perfusion declines significantly, a change that has been causally linked to cognitive decline and increased vulnerability to ischemic injury. The efficiency of brain perfusion depends on two critical factors: the density and the vasodilatory function of the microvasculature [761]. Age-related microvascular rarefaction is defined as the reduction in both the density and complexity of the cerebromicrovascular network. This reduction impairs the brain’s ability to maintain adequate blood flow, resulting in hypoperfusion, a state in which regions of the brain receive insufficient blood supply. Hypoperfusion increases the risk of ischemic injury and is implicated in the pathogenesis of VCID as well as other neurodegenerative conditions. Region-specific microvascular rarefaction in the brain has been well documented and linked to impaired cognitive function as well as susceptibility to ischemia [761–763]. Several mechanisms have been implicated in age-related microvascular rarefaction [251]. Endothelial cell senescence, chronic inflammation, and decreased nitric oxide bioavailability are all key contributors to the age-associated decline in endothelial angiogenic capacity and microvascular rarefaction [16, 251]. These changes contribute to reduced cerebral blood flow and increased vulnerability to ischemic events. There is strong evidence on the role of systemic factors in the regulation of brain microvascular density and perfusion (Fig. 9). In an initial study by Katsimpardi and colleagues the exposure of aged mice to young blood was suggested to increase both microvascular density and cerebral blood flow in the subventricular zone [50]. Using lege artis methods to evaluate microvascular density we have demonstrated that in a heterochronic parabiosis model, we observed that young blood significantly increased microvascular density and complexity in the somatosensory cortex, while aged blood had the opposite effect, reducing both vascular density and complexity [177]. This finding suggested that circulating factors in young blood could counteract the microvascular rarefaction associated with aging (Fig. 9). These studies highlight the role of systemic factors in maintaining and potentially rejuvenating cerebrovascular health in aging brains. The precise mechanisms and molecular mediators responsible for these effects remain under investigation, but evidence points to several key candidates.

IGF- 1 has emerged as a critical factor in regulating microvascular density and protecting against age-related vascular rarefaction. IGF- 1 signaling is essential for endothelial cell survival, angiogenesis, and vascular remodeling. IGF- 1 exerts its vasculoprotective effects by activating eNOS to increase NO production, which enhances vasodilation and promotes angiogenesis [262]. In aging, circulating levels of IGF- 1 decline, contributing to endothelial dysfunction and a loss of capillary networks [764]. Studies have demonstrated that IGF- 1 supplementation or activation of IGF- 1 signaling can reverse declines in microvascular density [764]. In models of brain ischemia, IGF- 1 has been shown to promote the proliferation and survival of endothelial cells, increase vessel density, and improve blood flow to affected regions, mitigating ischemic injury [765, 766]. In the context of young systemic milieu, IGF- 1 may be a key mediator of the observed increases in microvascular density following exposure to young blood.

Circulating VEGF plays a role in regulating cerebrovascular health and potentially modulating aging phenotypes within the brain [168, 767]. As a primary angiogenic factor, VEGF is essential for maintaining vascular integrity, promoting endothelial cell proliferation, and supporting neurovascular networks critical for cerebral blood flow. Age-related declines in VEGF levels [767] are strongly associated with reduced microvascular density [768], hypoperfusion, and increased susceptibility to ischemic injury [769], which collectively contribute to VCID.

In addition to IGF- 1 and VEGF, GDF- 11 has been identified as a rejuvenating factor with profound effects on cerebrovascular health [50]. GDF- 11, a circulating factor enriched in young blood, has been shown to improve microvascular density and cerebral blood flow in aging mice. Specifically, GDF- 11 promotes endothelial cell proliferation, reduces apoptosis, and enhances angiogenesis. In aged mice treated with GDF- 11, significant improvements were observed in vascular density, particularly in the subventricular zone and cerebral cortex [50, 203]. Moreover, GDF- 11 has demonstrated neuroprotective properties in models of ischemic injury. In these models, systemic administration of GDF- 11 reduced ischemic damage by promoting endothelial repair, decreasing inflammation, and enhancing mitochondrial function in affected brain regions [770–772]. These effects translated into improved neurological outcomes and reduced infarct volumes.

The age-related decline in microvascular density contributes to hypoperfusion, which exacerbates the risk of ischemic injury in the brain. In cases of ischemia, such as stroke, reduced perfusion results in energy failure, oxidative stress, and neuronal death. Studies have shown that young systemic factors, including GDF- 11 and IGF- 1, may protect against ischemic injury by promoting vascular regeneration, stabilizing endothelial cells, and reducing inflammatory responses. For instance, Pan et al. demonstrated that young plasma protected the brain from ischemic damage, reducing infarct size and mitigating neurological deficits [773]. In contrast, the aged systemic milieu exacerbated stroke outcomes, resulting in larger infarct volumes and significantly impaired neurological recovery [773]. The authors identified haptoglobin as a pro-ischemic factor, whose levels increase with age and contribute to the deleterious effects of aged plasma on ischemic injury [773]. Systemic administration of haptoglobin mimicked the detrimental effects of aged plasma in ischemic rats [773]. Furthermore, other systemic factors, e.g., CCL5 and CCL11, could systemically exacerbate consequences of stroke [774, 775]. In rat models of stroke, isochronic blood exchange (in which ischemic animals are treated with blood from non-ischemic rats) has shown beneficial effects, reducing infarct volume, protecting the BBB and improving neurological outcomes [776]. Furthermore, whole blood exchange improved systemic inflammation (by decreasing levels of proinflammatory mediators as IL- 6, TNFα, IL- 1B), reduced infiltration of immune cells to the brain and lowered brain and plasma MMP9 levels [776]. These findings further support the role of systemic factors in modulating the severity of ischemic damage and highlight the potential for therapies targeting the systemic environment to improve outcomes in stroke and related ischemic injuries.

#### Cerebral microhemorrhages

CMHs, also known as cerebral microbleeds, are small, chronic hemorrhagic lesions in the brain caused by rupture of fragile microvessels [17, 133]. They are a common finding in elderly populations, especially in individuals with vascular risk factors [724] such as hypertension [133]. CMHs are increasingly recognized due to the widespread use of magnetic resonance imaging (MRI), particularly gradient echo T2* sequences or susceptibility-weighted imaging (SWI), which are sensitive to hemosiderin deposits left by blood degradation products [777]. The prevalence of CMHs increases with age, affecting close to 50% of people over the age of 65. Their prevalence is particularly high in individuals with CSVD [778] and Alzheimer’s disease [779]. Aging and hypertension are the two most significant risk factors for CMHs [119]. Aging leads to structural and functional changes in the cerebral microvasculature [17, 119], including pathological remodeling of the ECM and the VSMCs, autoregulatory dysfunction and increased penetration of high-pressure waves int the vulnerable distant portion of the microcirculatory network. Hypertension exacerbates these changes by imposing mechanical stress on vessel walls, leading to vessel rupture in regions of least resistance. Together, these factors promote microvascular damage, contributing to the formation of CMHs. Other risk factors include cerebral amyloid angiopathy (CAA), a condition where amyloid-β deposits accumulate in the walls of cerebral microvessels, weakening the vessel structure and increasing the risk of CMHs [130]. CMHs are best detected using MRI, specifically gradient-echo T2* weighted sequences or SWI, which are highly sensitive to small hemorrhages [780, 781]. CMHs appear as punctate areas of signal loss due to the paramagnetic effect of hemosiderin. The detection of CMHs is critical as their presence is associated with cognitive impairment, dementia, an increased risk of more severe hemorrhagic strokes [782] and exacerbation of neurodegenerative diseases including AD. CMHs in subcortical regions, particularly in the basal ganglia and thalamus, can contribute to motor deficits [783, 784] and increased risk of falls.

The pathogenesis of CMHs involve an age-related increase in microvascular oxidative stress, MMP activation, dysregulation of ECM components and ECM degrading enzymes and pathological remodeling of the microvascular wall, making vessels more susceptible to hypertension-induced rupture [133]. Furthermore, aging is associated with the accumulation of senescent cells within the vessel wall [415]. These senescent cells secrete a range of proinflammatory and proteolytic factors known as the senescence-associated secretory phenotype (SASP) and contribute to the genesis of CMHs [415]. A critical component of the SASP is MMPs, which degrade extracellular matrix proteins and compromise the structural integrity of the vessel wall [415]. MMPs, particularly MMP- 9, are activated by oxidative stress and inflammation and are strongly implicated in the pathogenesis of CMHs by compromising the structural integrity of the microvascular wall [119]. It is likely that in aged and hypertensive individuals, the overactivation of MMPs results in excessive ECM degradation, leading to focal areas of weakness where increased wall tension-induced ruptures are likely to occur [119]. CMHs cause direct neuronal damage through the rupture of fragile microvessels, leading to localized bleeding in brain tissue. This localized bleeding not only results in the destruction of neurons at the hemorrhagic site but also initiates a cascade of secondary damage due to the surrounding ischemic penumbra [785].

There is increasing evidence suggesting that circulating factors play a significant role in the pathogenesis of CMHs, particularly in the context of aging and hypertension. Among these factors, age-related IGF- 1 deficiency has emerged as a critical contributor [131, 261, 786]. IGF- 1 is essential for maintaining microvascular health, particularly in preserving microvascular structural integrity and supporting both structural and functional adaptation to hypertension [261]. IGF- 1 promotes VSMC proliferation and survival, and its deficiency is strongly associated with microvascular maladaptation to high blood pressure, leading to increased microvascular fragility [131]. In the context of hypertension, IGF- 1 deficiency induces VSMC hypotrophy and pathological alterations in the ECM, making the microvasculature less resilient to the mechanical stress imposed by elevated blood pressure [658]. Without sufficient IGF- 1 input, endothelial cells and VSMCs acquire a pro-fragility phenotype [786, 787] and become more prone to apoptosis, and the weakened vessel walls are more susceptible to rupture, increasing the risk of CMHs [131]. Additionally, IGF- 1 plays a vital role in maintaining the integrity of the ECM, which supports vessel structure and function [658]. In the absence of adequate IGF- 1 signaling, ECM remodeling becomes dysregulated, contributing further to vessel wall instability [658]. Moreover, the cerebral circulation relies heavily on myogenic autoregulatory mechanisms to protect the microvasculature from high pressure waves that could otherwise damage the more distal and fragile segments of the vascular tree. In response to chronic hypertension, this autoregulatory mechanism is normally upregulated to shift the upper limit of autoregulation to higher pressure values, thereby providing functional adaptation to increased systemic blood pressure. However, in aging [117, 118, 120] and in the presence of IGF- 1 deficiency [788, 789], this myogenic autoregulatory adaptation is impaired, allowing excessive pressure to reach the vulnerable microcirculation. This results in greater susceptibility to damage, further elevating the risk of CMHs in hypertensive individuals. Additionally, deficient IGF- 1 input has been causally linked to increased microvascular senescence [258]. The combination of VSMC dysfunction, endothelial cell senescence, ECM degradation, and impaired autoregulatory adaptation in IGF- 1-deficient individuals creates a perfect storm, increasing the likelihood of microvascular damage and the development of CMHs, particularly in individuals with hypertension.

### Decline in adult neurogenesis, synaptic plasticity, and cognition

Cognitive decline with aging is influenced by several neuronal mechanisms, including reductions in adult neurogenesis [61, 63, 79, 128, 411, 790–792], synaptic plasticity, and overall neuronal function [62, 156, 574, 793, 794]. These processes are critical for maintaining cognitive abilities such as learning, memory, and executive function. Several intrinsic, cell-autonomous mechanisms such as mitochondrial dysfunction, oxidative stress, and DNA damage accumulate in aging neurons, leading to cellular senescence and reduced plasticity [795]. Alongside these intrinsic changes, recent research has highlighted the importance of systemic factors present in the blood, which significantly modulate neuronal health and cognitive function [50, 61, 62, 79, 188, 796]. Systemic factors, including cytokines, hormones, and growth factors, can accelerate or mitigate age-related declines in neurogenesis and synaptic plasticity [61, 63, 79, 791]. In the following section, we will explore how age-related changes in the systemic milieu contribute to the decline in neurogenesis, synaptic plasticity, and cognitive function, as well as interventions that can rejuvenate these processes.

Studies by Villeda et al. demonstrated that aged systemic milieu impairs neurogenesis, synaptic function, and spatial learning [63, 79]. This led to the identification of several circulating factors enriched in aged plasma, such as CCL2, CCL11 (eotaxin- 1), haptoglobin, and beta- 2 microglobulin (B2M), which may contribute to these detrimental effects on the brain [61, 79]. For instance, CCL11 has been shown to increase neuroinflammation, reduce spine density, and impair both neurogenesis and cognitive function [79, 218]. The age-related increase in both plasma and brain levels of B2M and TGF-β1, along with their negative effects on neurogenesis, has been confirmed by several studies [61, 180, 451]. In addition to these factors, other systemic mediators, such as sVCAM- 1 and ASM, have also been recently identified as contributors to age-related cognitive decline [193, 754]. Plasma concentrations of sVCAM- 1 and ASM are elevated with age and are strongly associated with decreased neurogenesis, increased neuroinflammation, and impaired hippocampal-dependent memory function [193, 754]. Furthermore, a study by Rebo et al. demonstrated the profound effects of the aged systemic milieu on neurogenesis, showing that even a single exchange of aged blood significantly reduced neurogenesis [180]. Given the complexity of age-related changes in the systemic milieu, which involve numerous pro-aging factors, therapeutic interventions aimed at diluting or modifying the aged milieu have been explored. One such intervention, neutral blood exchange, involves replacing aged blood with a 5% albumin solution to dilute the harmful circulating factors. This treatment has shown efficacy in preclinical studies, where it was found to reduce neuroinflammation, enhance neurogenesis, and improve cognitive outcomes such as novel object recognition [206, 214]. The beneficial effects of this approach provide insight into the role of systemic factors in age-related cognitive decline, demonstrating the potential of systemic interventions to reverse aspects of aging in the brain. Further details on various interventions aimed at rejuvenating neurogenesis, restoring neuronal functions, and improving cognitive outcomes are discussed in Table 1.

**Table 1:** Systemic milieu and its impact on neurogenesis, neuroinflammation, cognitive function, and cerebrovascular health

Systemic Fraction (putative factors)	Effects on neuronal networks	Behavior and cognitive effects	Neuroinflammation and brain senescence	Effects on cerebrovasculature	References
Protective systemic fractions					
Young blood (GDF11, Oxytocin, sKlotho)	↑ Neurogenesis^a−c^ ↑ Synaptic plasticity^c^ ↑ Dendritogenesis^c^ ↑ LTP^c^	↑ Exploratory behavior^b^	↓ p21, Mcp1, and IL- 6 expression in forebrain^e^	↑ Vessel Density and perfusion in the SVZ^b^ Transcriptomic rejuvenation of BECs^d^	^a^Conboy, 2015 ^b^Katsimpardi, 2014 ^c^Villeda, 2011/14 ^d^Ximerakis, 2023 ^e^Yousefzadeh, 2020
Young plasma (THBS4, SPARCL1)	↑ Neurogenesis^a−c^ ↑ Synaptogenesis^a^ ↑ Dendritogenesis^a^ ↑ Synaptic activity^a^	↑ Contextual fear conditioning^c^ ↑ Spatial learning^c^	?	↓ Expression of inflammation-related genes in BECs	^a^Gan, 2019 ^b^Rebo, 2016 ^c^Villeda, 2014 ^d^Chen, 2020
Umbilical cord plasma (TIMP2)	↑ Neurogenesis^a−c^ ↑ Synaptogenesis^a^ ↑ LTP^b,c,d^ ↑Neuronal plasticity^b,d^	↑ Spatial learning^b,d^ ↑ Contextual learning^d^	↓ Microglial activation^a^	?	^a^Bachstetter, 2008 ^b^Cao, 2017 ^c^Cui, 2017 ^d^Castellano, 2017
Young BMT (Mesenchymal stem cells)	↑ Synaptic density^a,b^	↑ Exploratory behaviors^a^ ↑ Contextual learning^a^ ↑ Spatial learning^b^	↓ Microglial and astrocytic activation^a,b^	?	^a^Das, 2019 ^b^Nakano, 2020
Platelets (PF4)	↑ Neurogenesis^a−c^ ↑ Synaptic plasticity-related gene expression^a^	↑ NOR^b,c^ ↑ Spatial learning^b,c^ ↑ Contextual learning^b,c^	↓ Activation of microglia and complement system^b,c^	↑ Angiogenesis in the SVZ^a^	^a^Hayon, 2013 ^b^Schroer, 2023 ^c^Leiter, 2023
Plasma dilution	↑ Neurogenesis^a^	↑ NOR^b^	↓ Microglial activation^b^ ↓ Brain senescence^b^	?	^a^Mehdipour, 2020 ^b^Mehdipour, 2021
Progeronic systemic fractions					
Aged blood	↓ Neurogenesis^a,b^ ↓ LTP^a^	?	↑ Expression of Mcp1 and IL- 6 in forebrain^c^	(–) Vessel density and perfusion in the SVZ^d^	^a^Villeda, 2011 ^b^Rebo, 2016 ^c^Yousefzadeh, 2020 ^b^Katsimpardi, 2014
Aged plasma (CCL- 11, B2M, sVCAM- 1, ASM)	↓ Neurogenesis^a,b^	↓ Contextual fear conditioning^a^ ↓ Spatial learning^a^	↑ Microglial and vascular inflammation^b^	↑ Vascular inflammation^b^ ↑ BBB permeability^c^	^a^Villeda, 2011/14 ^b^Yousef, 2019 ^c^Park, 2018
Aged BMT (cyclophylin A)	↓ Neurogenesis^a^	↓ Contextual fear conditioning^a^ ↓ Spatial learning^a^	?	?	^a^Smith, 2020

Superscript letters (a–e) indicate the respective primary literature sources supporting the findings described in each row of the table

Exposure to young systemic milieu has been shown to provide broad rejuvenation of neuronal function and hippocampus-dependent learning and memory. Early studies employing heterochronic parabiosis demonstrated that young blood contains systemic factors, metabolites, and cells capable of improving neurogenesis and cognitive functions [50, 62]. These studies revealed that exposure to young blood improved vascular density and brain perfusion in the subventricular zone of the hippocampus [50]. In an in vitro study, Gan and colleagues demonstrated that cultured neurons treated with young plasma exhibited improved neuronal and synaptic activity [797]. The beneficial effects on cultured neurons were attributed to young plasma-enriched proteins, such as SPARCL- 1 and THBS4 [797].

One key systemic factor with a significant impact on neurogenesis and synaptic plasticity is IGF- 1 [574, 791, 798–802]. IGF- 1 levels decline with age, and its deficiency has been closely linked to reductions in neurogenesis and synaptic function [791, 792]. IGF- 1 promotes neuronal survival and differentiation by activating the IGF- 1R on neurons and other brain cells [803]. These pathways are critical for maintaining synaptic plasticity and promoting neuronal survival. Changes in IGF-I levels are positively correlated with hippocampal volumes in older adults [804–807]. Restoring IGF- 1 levels or enhancing IGF- 1 signaling has been shown to mitigate age-related cognitive decline by promoting hippocampal neurogenesis and enhancing synaptic plasticity [791, 801, 802]. In animal models, boosting IGF- 1 levels leads to improved performance on memory and learning tasks, indicating its potential as a therapeutic target for age-related cognitive decline [802]. Collectively, these studies illustrate the significant potential of young systemic milieu in promoting neurogenesis and enhancing neuronal activity. However, existing studies suggest that while the aged systemic milieu exerts profound detrimental effects, the neurogenic benefits of young blood appear to be comparatively less pronounced [180].

In addition to IGF- 1, several other circulating factors found in young blood have been identified as potent modulators of neurogenesis and synaptic plasticity. BDNF plays a critical role in promoting neurogenesis and enhancing synaptic plasticity, particularly in the hippocampus, by facilitating learning and memory formation [806, 808]. GDF- 11, another key factor, has been shown to rejuvenate the aging brain by increasing neural progenitor cell proliferation and improving synaptic function [50, 809–811]. Similarly, FGF21 has neuroprotective effects, mitigating inflammation and oxidative stress while promoting synaptic plasticity [812, 813]. Klotho, an anti-aging protein, enhances cognitive function by regulating synaptic plasticity [814–816] and reducing neuroinflammation [817, 818]. Together, these factors are present in young blood and represent promising targets for therapies designed to rejuvenate the aging brain and reverse age-related cognitive decline.

Besides young plasma, other blood fractions, such as human umbilical cord blood (hUCB) and platelet derivatives, have been assessed for their rejuvenative properties on neurogenesis and cognition [277, 819–821]. The hUCB contains all blood cells and is enriched with growth hormones and a favorable cytokine profile. In the brain, treatment with hUCB has been shown to mitigate neuroinflammation by reducing the expression of pro-inflammatory mediators like TNF, C1qb, and CD11b, as well as by decreasing microglial activation [758]. This treatment also improved various aspects of cognition, including object recognition, spatial learning, and contextual learning [758]. Various fractions of umbilical cord blood contribute to these improvements. For example, UCB-derived mononuclear cells (specifically T cells) have been shown to increase the proliferation of neural stem cells [758]. Moreover, mesenchymal stem cells derived from umbilical cord blood have been shown to improve synaptic density, neurogenesis, hippocampal long-term potentiation, and spatial learning in aged mice and mouse models of AD [796, 822]. The acellular fraction of hUCB also provides neuroprotective effects, improving neuronal plasticity, function, and cognition [192]. Notably, TIMP2, a key protein identified from umbilical cord blood proteome studies, has been validated as a critical mediator of hUCB-induced brain rejuvenation [192]. Another source of systemic rejuvenation is young bone marrow, which is enriched in hematopoietic stem and progenitor cells as well as various growth factors. Transplantation of young bone marrow has been shown to decrease plasma levels of CCL11, reduce neuroinflammation, and improve cognitive outcomes such as exploratory behavior and contextual learning [218]. Furthermore, Nakano and colleagues demonstrated that bone marrow-derived mesenchymal stem cells alone were able to replicate these cognitive benefits [757].

Additionally, platelets and their proteome have shown rejuvenative effects on the aging brain [823]. In preclinical stroke models, treatment with platelet lysate promoted recovery, decreased infarct volume, enhanced re-vascularization, and increased neurogenesis [824]. In aged mice, systemic administration of young platelet fraction mitigated neuroinflammation, reduced pro-inflammatory cytokine expression, and improved cognition, including object recognition, spatial learning, and contextual learning [755, 756]. PF4 (platelet factor 4), a factor derived from platelets, has been identified as a key contributor to these improvements, reducing levels of proinflammatory mediators and enhancing hippocampal neurogenesis and memory [755, 756].

### Neurodegenerative diseases

Neurodegenerative diseases are a group of age-related disorders characterized by the progressive loss of neurons, resulting in lesions, atrophy within the central nervous system, and significant cognitive and motor deficits. Some of the most common neurodegenerative diseases include AD and Parkinson’s disease (PD) [825]. The pathogenesis of neurodegenerative diseases varies between diseases, but there are many shared mechanisms, including the loss of proteostasis, protein aggregation (e.g., amyloidopathies, tauopathies), oxidative stress, neuroinflammation, and the presence of autoantibodies and reactive oxygen species-mediated damage [825–831]. Many of these mechanisms can be modulated by the systemic milieu, and they are exacerbated during aging. Immunosenescence and systemic inflammation also play a role, particularly through the accumulation of senescent cells and increased levels of circulating proinflammatory cytokines. Additionally, there is growing evidence that extracellular vesicles, circulating miRNAs, and factors derived from the gut microbiome contribute significantly to the pathogenesis and progression of neurodegenerative diseases [832, 833].

#### Alzheimer’s disease

Alzheimer’s disease is the most prevalent neurodegenerative disorder, affecting millions of individuals worldwide. AD is characterized by extracellular deposition of amyloid-β plaques, intracellular accumulation of hyperphosphorylated tau in the form of neurofibrillary tangles, synaptic dysfunction, neuroinflammation, cerebral amyloid angiopathy, and multifaceted neurovascular dysfunction [148, 834]. These pathological features lead to neuronal loss, brain atrophy, and progressive cognitive decline, memory deficits, ultimately resulting in dementia. While the precise etiology of AD remains unclear, there is a growing body of evidence suggesting that microvascular dysfunction plays a critical role in the pathogenesis and progression of the disease [148, 834]. A number of microvascular mechanisms, such as BBB disruption [249], CMHs [133, 835], neurovascular uncoupling [836–839], and impaired clearance of toxic metabolites, have been implicated in the progression of AD. For instance, BBB disruption, a hallmark of cerebrovascular aging, allows harmful blood-borne molecules, such as Aβ, to infiltrate the brain, promoting inflammation and neuronal damage. Microvascular rarefaction, a reduction in the density of small vessels, can exacerbate hypoperfusion, reducing oxygen and nutrient delivery to neurons and impairing the clearance of metabolic waste [840]. This further contributes to the buildup of Aβ and other toxic proteins within the perivascular space, potentially serving as a nidus for amyloid plaque formation. Interestingly, amyloid plaques have been observed to develop around the vasculature in perivascular spaces, reinforcing the concept that AD pathology may be initiated in the microvasculature before spreading to the rest of the brain [249, 841–843]. Additionally, neurovascular uncoupling—an age-related phenomenon where the brain’s ability to regulate blood flow in response to neuronal activity becomes impaired—leads to further hypoperfusion and neuronal stress in AD [836–839]. A well-functioning neurovascular unit is essential for clearing Aβ through the perivascular glymphatic system. When this system is compromised due to age-related or disease-induced alterations in blood vessel function, it further contributes to the accumulation of toxic proteins, exacerbating neurodegeneration [278, 844]. These findings suggest that microvascular dysfunction, including impaired clearance of Aβ, BBB disruption, and cerebrovascular structural changes, may not only exacerbate AD but could serve as one of the earliest drivers of its pathogenesis. Moving forward, studies aimed at rejuvenating the microvascular and neurovascular systems, potentially through young blood-derived factors, may offer new insights and therapeutic strategies for halting or reversing AD progression.

The involvement of systemic factors in AD pathogenesis has received increasing attention, as growing evidence suggests that AD is not solely a localized brain disease but can be systematically influenced by circulating factors. There is substantial preclinical evidence showing the beneficial effects of young systemic milieu in mitigating AD symptoms [57, 58, 845–852]. Previous studies, such as those by Middeldorp et al., employed parabiosis (where a healthy young mouse is connected to an older APP/PS1 mouse model of AD) to demonstrate that exposure to young blood can significantly reduce amyloid-β plaque burden, restore synaptic plasticity-related proteins, and improve cognitive function, such as performance in the Y-maze and novel object recognition tests [58]. These improvements were attributed to rejuvenating factors in young plasma, as subsequent experiments using repeated injections of young plasma (without parabiosis) replicated the beneficial effects [847]. Among the factors enriched in young plasma, BDNF and nerve growth factor (NGF) were suggested to contribute to enhanced synaptic plasticity and neuroprotection [58]. These findings have been validated in other mouse models of AD, where young plasma treatments reduced the burden of amyloid plaques and phosphorylated tau, decreased the activation of microglia and astrocytes, and improved both object and spatial memory [57, 853, 854]. Interestingly, plasma from exercised animals appeared to have an even stronger effect on AD symptoms [57]. Kim et al. showed that plasma derived from exercised mice reduced hippocampal mitochondrial ROS levels, apoptosis, and tau phosphorylation, and improved spatial memory in AD models [57]. These findings suggest that exercise-induced factors in plasma, including elevated BDNF, may play an essential role in cognitive protection [57]. Whole blood exchange has also been explored as a potential treatment. Studies in Tg2576 AD mice demonstrated that monthly whole blood exchange starting at 3 or 13 months of age prevented and even reversed AD-related symptoms, including amyloid-β accumulation, memory deficits, and increased levels of soluble and insoluble amyloid-β peptides (Aβ40 and Aβ42) [855]. However, the safety of this treatment remains a concern, as lower survival rates were observed in treated animals compared to controls.

Therapeutic blood exchange (TBE) has emerged as a promising translational approach to reduce the systemic factors that exacerbate AD pathology. TBE involves replacing a patient’s blood with a blood product or plasma that is low in toxic proteins, proinflammatory mediators, and protein aggregates. Recent studies have demonstrated the safety and efficacy of TBE in clinical settings, particularly in decreasing plasma levels of Aβ42 and increasing its clearance from the CSF [856–859]. Patients undergoing TBE showed improvements in cognitive performance (e.g., Boston Naming Test, Semantic Verbal Fluency) and daily living activities, alongside reduced rates of brain atrophy and metabolic dysfunction [856–860]. TBE was well tolerated, with relatively low rates of adverse events (12.5–17%) [857]. Additionally, two preliminary trials (with *n* = 47 and *n* = 18, respectively) explored the effects of young fresh frozen plasma in AD patients, reporting moderate improvements in cognitive and functional outcomes [196, 846].

Emerging studies suggest that systemic factors enriched in aged blood or blood from AD patients may contribute to the propagation of disease. As discussed in the “Decline in adult neurogenesis, synaptic plasticity, and cognition” section on adult neurogenesis, aged blood impairs neurogenesis and cognitive function through proinflammatory factors such as CCL11 (eotaxin- 1) and beta- 2 microglobulin (B2M) [61, 176]. Recent research has shown that CCL11 directly contributes to neurodegeneration [176]. Elevated levels of circulating CCL11 have been observed in patients with AD, as well as other neurodegenerative diseases [861]. CCL11 has been negatively associated with cognitive performance, suggesting that it could serve as a biomarker for neurodegeneration [862]. In addition, B2M has been linked to impaired neurogenesis, worse cognitive function, and neurodegeneration [61, 863].

IGF- 1 plays a pivotal role in promoting neuronal survival, synaptic plasticity, and cognitive function. Its levels decline with age, a reduction that has been strongly linked to cognitive deficits and heightened vulnerability to neurodegenerative diseases such as AD [864–869]. IGF- 1 exerts its neuroprotective effects by modulating key signaling pathways that influence neuronal and astrocyte health, including pathways involved in oxidative stress resistance, inflammation control, and synaptic maintenance [870–874]. Preclinical studies have demonstrated that IGF- 1 supplementation can reduce amyloid plaque formation, enhance synaptic density, and improve cognitive performance in AD models, highlighting its therapeutic potential [870]. However, despite these promising findings, there is also evidence suggesting potential challenges and controversies in the application of IGF- 1 as a therapeutic strategy. Some preclinical and clinical studies have reported limited efficacy or even detrimental effects of IGF- 1 in specific contexts [274, 875–878]. These conflicting results underscore the need for a nuanced understanding of IGF- 1’s role in neurodegenerative diseases and the mechanisms through which it mediates its effects.

GDF- 11 has also emerged as a potent factor with neuroprotective properties [809, 879]. Recombinant GDF- 11 administration has been shown to improve short-term memory and increase neural stem cell proliferation in the hippocampus [60]. Furthermore, studies have demonstrated that GDF- 11 enhances cognitive function by influencing Sox2 expression, a key factor in neurogenesis [60, 880]. Conversely, proinflammatory and neurotoxic factors like CCL11 have been implicated in exacerbating AD pathology by promoting neuroinflammation and neurodegeneration [861]. An interesting piece of evidence supporting the systemic propagation of AD pathology comes from a study by Bu et al., which demonstrated that amyloid-β (Aβ) can spread through the bloodstream [826]. Using a parabiosis model, where Aβ-transgenic mice were connected to wild-type mice, the study showed that blood-derived Aβ protein could cross the BBB and accumulate in the brain parenchyma of wild type mice [826]. After 12 months, the WT mice exhibited Aβ-related pathologies, including deficits in hippocampal neurons and features of cerebral amyloid angiopathy [826]. This study provided the first direct evidence that misfolded Aβ in the blood can induce AD-like brain pathologies, highlighting the potential of systemic factors to propagate disease. This complex interplay between beneficial and detrimental factors in the systemic environment underscores the potential of modulating the systemic milieu to influence AD progression. Given the increasing evidence that microvascular dysfunction plays a central role in AD pathogenesis, future studies should aim to determine whether rejuvenating microvascular and neurovascular systems can delay or reverse AD progression in human patients. Interventions aimed at restoring BBB integrity, improving neurovascular coupling, and enhancing the clearance of toxic proteins from the brain may hold promise for modifying the course of AD. Moreover, understanding the role of young blood factors in modulating these microvascular and neurovascular mechanisms could lead to the development of novel therapies targeting early AD pathology. Future research should focus on identifying the key factors in young plasma that contribute to microvascular rejuvenation and investigating their therapeutic potential in preclinical and clinical models of AD.

#### Parkinson’s disease

Parkinson’s disease is a common neurodegenerative disorder characterized by the progressive loss of dopaminergic neurons in the substantia nigra pars compacta, a brain region critical for motor control. The hallmark motor symptoms of PD include bradykinesia (slowness of movement), resting tremor, muscle rigidity, and postural instability. PD is also associated with non-motor symptoms such as mood disorders (e.g., depression, anxiety), cognitive impairments, sleep disturbances, dysphagia, and autonomic dysfunction. Despite extensive research, the precise mechanisms underlying neuronal loss in PD remain incompletely understood [881]. Several hypotheses have been proposed to explain the pathogenesis of PD, with evidence pointing to a combination of factors, including progressive accumulation of alpha-synuclein protein, impaired proteostasis, mitochondrial dysfunction, oxidative stress, genetic predisposition, environmental toxins, and neuroinflammation [881–883]. A key feature of PD pathogenesis is the misfolding and aggregation of alpha-synuclein into Lewy bodies, which are found in affected neurons. Alpha-synuclein pathology spreads in a prion-like manner, promoting neurodegeneration across brain regions involved in motor and cognitive functions. Neuroinflammation plays a significant role in PD, tightly linked to a heightened state of inflammation in the systemic circulation [884]. Research has shown elevated levels of proinflammatory mediators in the blood of PD patients [885]. Although studies on plasma biomarkers in PD have yielded mixed results, several inflammatory mediators, such as IL- 1β, IL- 6, TNF-α, CCL5, and CRP have been implicated in the disease [886]. Elevated plasma levels of IL- 6, and TNF-α have been negatively correlated with motor and cognitive outcomes in PD, suggesting that systemic inflammation may exacerbate both motor dysfunction and cognitive decline [887].

Recent research has identified specific circulating factors that may contribute to neurodegeneration in PD. Although levels of CCL- 11 (eotaxin- 1), a chemokine linked to aging and cognitive decline, are not elevated in PD patients, studies using murine models of PD have demonstrated that neutralizing systemic levels of CCL- 11 and CCL- 5 can protect the nigrostriatal pathway from neurodegeneration [888]. In contrast, administering CCL- 11 and CCL- 5 to PD models induced alpha-synuclein aggregation, dopaminergic neuron loss, and worsened motor function [889]. These findings suggest that circulating chemokines play a role in exacerbating PD pathology. Levels of CCL- 5, in particular, are elevated in the plasma of PD patients and correlate with disease severity, further implicating it in the progression of neurodegeneration [890, 891]. Exosomes, which are small extracellular vesicles that carry proteins, lipids, and nucleic acids, are increasingly recognized for their role in PD pathogenesis. Han et al. demonstrated that exosomes isolated from the plasma of PD patients could induce PD-like pathology when injected into mice [892]. The exosomal cargo from PD patients contained high levels of alpha-synuclein, IL- 1β, and TNF-α, and their administration led to protein aggregation, neurodegeneration, neuroinflammation, and motor dysfunction in the recipient mice [892]. This suggests that exosomes act as vehicles for the systemic spread of pathological proteins and inflammatory mediators in PD.

Heterogenic parabiosis models, where a PD mouse is surgically joined to a healthy wild-type mouse, have provided further evidence of the systemic nature of PD pathology. These studies confirmed that alpha-synuclein can be transmitted through the bloodstream from a PD mouse to a WT mouse, although the progression of PD symptoms in this model has yet to be fully explored [893]. Promising research has investigated the therapeutic potential of young plasma transfusions for PD. Two small clinical trials (NCT02968433, *n* = 15, and NCT03713957, *n* = 51) recently explored the safety and feasibility of young plasma transfusions in PD patients. The results demonstrated that young plasma was well tolerated, with minimal adverse effects. Preliminary data suggested slight improvements in PD-related symptoms, including motor function, and reductions in systemic TNF-α levels [195]. While these studies provide encouraging results, larger phase II clinical trials focused on treatment efficacy are needed to determine whether young plasma or plasma-derived factors can significantly alter the course of PD.

In summary, growing evidence supports the role of cell non-autonomous mechanisms, including systemic inflammation and circulating factors, in the pathogenesis and progression of PD. Peripheral immune activation and proinflammatory mediators, such as CCL- 11 and CCL- 5, appear to exacerbate alpha-synuclein pathology and neuronal loss. Further research is needed to elucidate the full range of systemic factors involved and to explore potential therapeutic strategies, such as young plasma transfusions, that may target these extrinsic contributors to PD.

## Putative anti-geronic and pro-geronic circulating factors that may regulate cerebrovascular and brain aging processes

Circulating factors, which may either promote or delay aging processes, include a wide range of molecules such as hormones, growth factors, cytokines [745], microRNAs, and other signaling proteins that regulate key cellular processes like proliferation, inflammation, oxidative stress, neurogenesis, and vascular health. In the context of cerebrovascular and brain aging, circulating factors play critical roles in modulating vascular homeostasis, blood–brain barrier integrity, neuroinflammation, neurogenesis, synaptic plasticity, and cognitive function. Pro-geronic factors contribute to the decline in vascular and neuronal health, promoting age-related diseases such as VCID and neurodegenerative diseases. Conversely, anti-geronic factors, which are more abundant in youth, have been shown to protect brain and vascular function, promote tissue regeneration, and potentially reverse age-related dysfunction. This section will explore key circulating factors that influence brain and vascular aging, focusing on both pro-geronic and anti-geronic factors. We will discuss how these factors originate from various tissues, including the brain [894], liver [264], muscle, the gastrointestinal tract, and adipose tissue [895, 896], and how their systemic effects contribute to the pathophysiology of brain aging. Additionally, we will review therapeutic interventions aimed at modulating these circulating factors, such as growth hormone supplementation, anti-inflammatory therapies, and young blood treatments, with the goal of rejuvenating cerebrovascular and brain health. Understanding the balance between pro-geronic and anti-geronic factors will be critical for developing novel therapeutic strategies to combat neurodegenerative diseases and age-related cognitive decline.

### Brain-derived factors

#### Growth hormone

Growth hormone (GH) is a 191 amino acid, single-chain peptide hormone produced and secreted by somatotrophic cells in the anterior pituitary gland. GH plays a crucial role in growth, metabolism, and cellular proliferation by acting directly on target tissues through the growth hormone receptor (GHR) and indirectly through the induction of IGF- 1 production in the liver and peripheral tissues [897]. GH exerts its effects through several signaling pathways, including the Janus kinase (JAK)-signal transducer and activator of transcription (STAT) pathway, MAPK-ERK signaling, and phosphoinositide 3-kinase (PI3 K)-Akt signaling. These pathways regulate processes critical to growth, such as cell proliferation, differentiation, protein synthesis, and lipid and carbohydrate metabolism [898]. Plasma levels of GH reach their peak during adolescence, supporting the growth and development of the body. However, as individuals age, circulating levels of GH gradually decline, a process referred to as somatopause [897, 899]. This age-related decline in GH levels contributes to the physical and cognitive decline seen in aging populations [900, 901]. The reduction in GH also leads to decreased production of IGF- 1, and consequently reduced IGF- 1 levels in the brain, which further impairs tissue maintenance and repair, as well as vascular health, and may contribute to age-related cognitive decline and neurodegenerative processes [573, 902–904].

In a recent heterochronic parabiosis study, the GH/IGF- 1 axis has been identified as a potentially important factor involved in rejuvenation of cerebrovasculature [168]. In a recent follow-up study, we demonstrated that the GH/IGF- 1 signaling axis plays a critical role in mediating the reversal of neurovascular uncoupling, BBB disruption, and microvascular rarefaction in response to young blood exposure (Gulej et al., in preparation). While direct studies of GH in parabiosis models are limited, it is known that IGF- 1, a downstream effector of GH, plays a significant role in promoting neuronal plasticity, enhancing synaptic function, and supporting neurogenesis [573, 905]. These findings suggest that GH and IGF- 1 could be vital factors in the rejuvenating effects observed in such models, though further research is needed to elucidate the exact mechanisms.

GH has significant effects on cardiovascular health, primarily through its actions on endothelial function and lipid metabolism [906]. Patients with GH deficiency are at increased risk for developing cardiovascular diseases due to a combination of factors, including dyslipidemia, increased visceral fat, insulin resistance, and hypertension. GH exerts vasoprotective effects by enhancing endothelial NO production, promoting vasodilation, and reducing oxidative stress [906, 907]. Additionally, GH supports angiogenesis, which is critical for maintaining adequate blood flow [764, 908, 909]. GH replacement therapy in deficient individuals has been shown to improve cardiovascular outcomes, although long-term studies are still required to fully assess the risks and benefits.

GH and IGF- 1 are also implicated in cognitive function, particularly in maintaining hippocampal neurogenesis and synaptic plasticity, processes that are essential for learning and memory, effects mediated through IGF- 1 [910, 911]. In aging individuals, the decline in GH and IGF- 1 levels correlates with cognitive decline, including deficits in memory, attention, and executive function, while GH substitution therapy resulted in improved short and long-term memory [912, 913]. Animal studies have demonstrated that GH administration can enhance neurogenesis, restore synaptic plasticity, and improve cognitive performance, particularly in models of aging and neurodegenerative diseases [914, 915]. However, while these findings are promising, the clinical efficacy of GH as a cognitive enhancer in humans remains under investigation. Despite the potential benefits of GH replacement therapy in aging and GH-deficient individuals, there are significant concerns regarding the side effects and long-term risks associated with its use. GH treatment can lead to several adverse effects, including carpal tunnel syndrome, arthralgia, insulin resistance, and an increased risk of developing diabetes. Moreover, there is evidence suggesting that GH treatment may increase the risk of certain cancers, particularly in older adults, as GH and IGF- 1 promote cellular proliferation [916, 917]. Due to these risks, GH therapy for anti-aging purposes is not approved and is considered unethical in many countries.

#### Oxytocin

Oxytocin, a peptide hormone synthesized in the hypothalamus and released through the posterior pituitary gland, is traditionally associated with uterine contractions and lactation. Beyond these roles, oxytocin also acts as a neuropeptide, influencing social bonding, emotional regulation, and cognitive functions such as memory and learning. As both a circulating hormone and neuromodulator, oxytocin affects various brain regions and holds significance in cognitive health, especially with aging. Aging impacts oxytocin levels and its signaling pathways, though research in humans is limited and somewhat inconsistent [918–920]. Animal studies, however, provide insights: in aged male mice, reduced oxytocin levels correlate with diminished tissue regenerative capacity [204]. Notably, restoring oxytocin in aged mice improves muscle regeneration by activating satellite cells [204] and enhancing liver repair through autophagy [921], indicating a role in tissue maintenance. As a neuromodulator, oxytocin also supports cognitive health by enhancing hippocampal neurogenesis, long-term potentiation, and synaptic plasticity, all of which are critical for memory and learning [922–924]. Oxytocin has broad cardioprotective effects, including antioxidant, anti-inflammatory, and pro-vasodilatory properties that protect against ischemic injury and reduce cardiovascular risk factors [925–928]. Its roles in mitochondrial health, autophagy, and endothelial function are particularly relevant in counteracting age-related vascular deterioration [929, 930]. Decreased oxytocin levels with age have been associated with sarcopenia, osteoporosis, and cardiovascular decline, suggesting it may be a valuable target for therapies aimed at addressing age-related conditions [925, 931–933].

##### MANF

Mesencephalic astrocyte-derived neurotrophic factor (MANF) (also known as arginine-rich, mutated in early-stage tumors (ARMET) or arginine-rich protein (ARP)) is a circulating protein encoded by the *Manf* gene. Levels of MANF decrease with age in model organisms, mice and humans, and MANF overexpression extends lifespan in flies [934]. A recent study reported that liver rejuvenation by heterochronic parabiosis in aged mice depends, at least in part, on MANF [934], which reduces inflammation and attenuates tissue damage. The causal role of MANF in hepatoprotection was confirmed by the findings that MANF heterozygous mice exhibit progressive liver damage, fibrosis, and steatosis and that overexpression of MANF confers protection against liver inflammation, fibrosis and hepatosteatosis [934]. Neuroprotective and anti-aging actions of MANF have also been documented both in the brain [935–939] and the retina [940]. Importantly, MANF is expressed in the vasculature [941] and it was shown to exert cerebrovascular protective effects, promoting angiogenesis in the brain [941]. Taken together, further studies are warranted to determine the role of MANF, if any, in cerebrovascular and brain rejuvenation induced by heterochronic parabiosis.

### Hepatokines

Hepatokines are liver-derived proteins that play essential roles in systemic metabolic regulation and influence various physiological processes beyond the liver. Emerging research suggests that hepatokines fundamentally impact cerebrovascular and brain aging pathways.

### IGF- 1

**Fig. 10:**
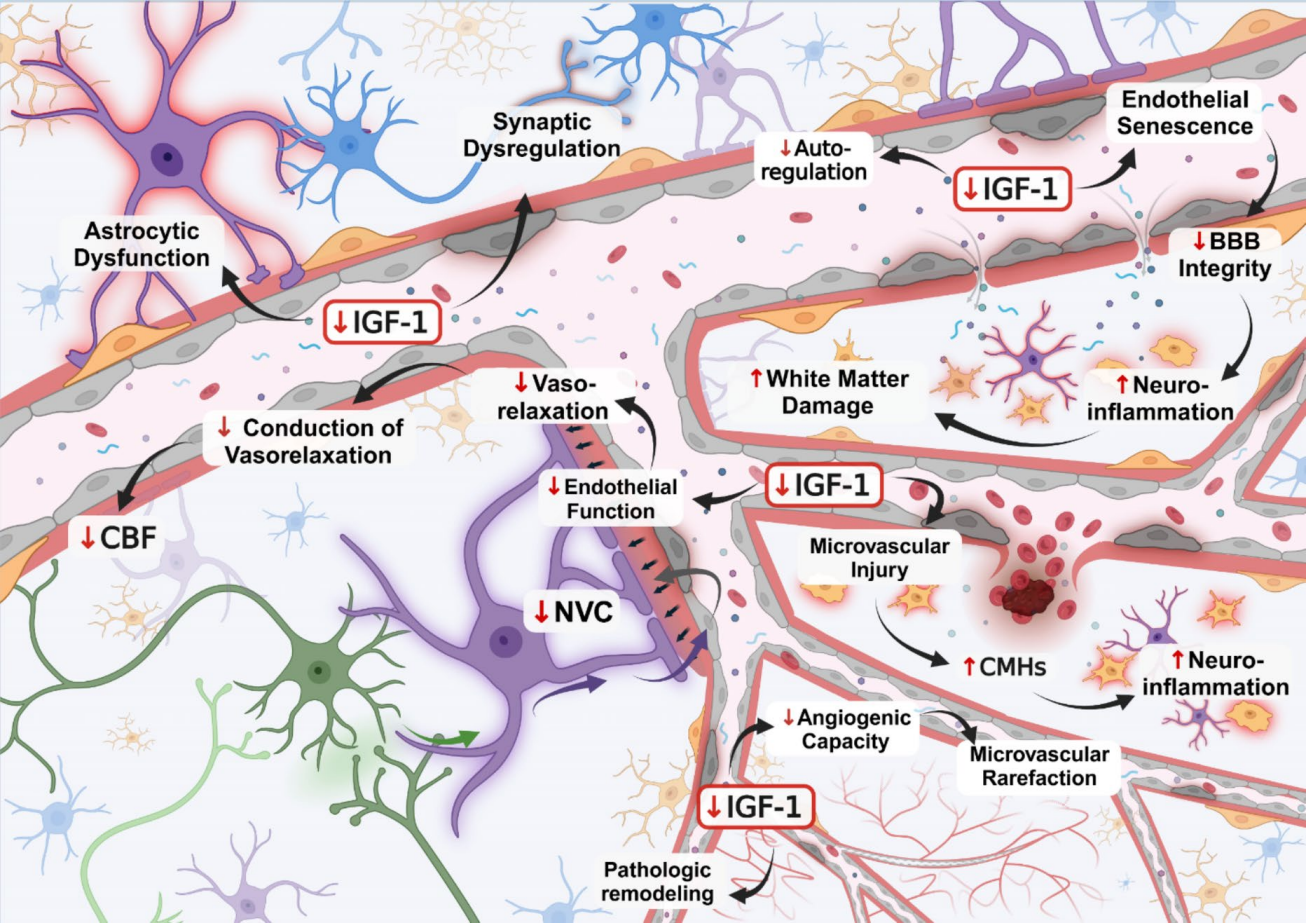
Cerebrovascular consequences of age-related deficiency in IGF- 1 signaling. This schematic highlights the multifaceted effects of age-related decline in insulin-like growth factor 1 (IGF- 1) signaling on cerebrovascular and neural health. IGF- 1, a pleiotropic vasculoprotective factor, is critical for maintaining neurovascular coupling (NVC), endothelial function, myogenic autoregulatory function, blood–brain barrier (BBB) integrity, capillarization, and structural integrity of the vascular wall. Age-related IGF- 1 deficiency impairs microvascular dilation, diminishes the conduction of vasodilatory signals, resulting in reduced cerebral blood flow (CBF). Declining IGF- 1 levels also promote endothelial senescence, increasing BBB permeability, which facilitates neuroinflammation, white matter damage, and neurodegeneration. Furthermore, IGF- 1 deficiency contributes to microvascular injury, rarefaction, pathological vascular remodeling and compromised myogenic autoregulatory protection, exacerbating ischemic vulnerability and the risk of cerebral microhemorrhages (CMHs). Collectively, these IGF- 1 deficiency-driven cerebromicrovascular pathologies culminate in cognitive impairment and highlight the critical role of IGF- 1 in maintaining cerebromicrovascular and brain health during aging

IGF- 1 is a critical anabolic hormone that regulates numerous cellular processes, including growth, differentiation, mitochondrial function, oxidative stress resilience, and tissue repair. Hepatic production of IGF- 1 is regulated by GH levels [942, 943]. IGF- 1 exerts its effects primarily through the IGF- 1R, activating downstream pathways like the MAPK-ERK and PI3 K-AKT signaling cascades, which promote cellular survival and function. In a recent study, IGF- 1/IGF- 1R signaling was identified as a likely upstream regulator involved in young blood-mediated transcriptomic rejuvenation of the aorta in aged heterochronic parabiont mice [51]. Inhibition of pathways regulated by IGF- 1 has also been implicated in old blood-mediated acceleration of vascular aging processes in young heterochronic parabiont mice [241]. Epidemiological studies and preclinical investigations also support the concept that circulating IGF- 1 acts as a young blood factor. IGF- 1 is a pleiotropic anabolic hormone that regulates cell growth, differentiation, development, mitochondrial function, production of reactive oxygen species, cellular stress resilience and tissue repair and confers multifaceted anti-aging effects in the central nervous system [97, 98, 109, 264, 573–575, 766, 788, 791, 870, 872, 944–956], the cardiovascular system in general [263, 571, 695, 957–962] and the cerebral circulation in particular [97, 98, 109, 131, 573, 658, 695, 765, 788, 963] as well as the musculoskeletal system [562, 563, 964–976]. Circulating levels of IGF- 1 are elevated in young adults and significantly decrease with age [558]. Additionally, levels of IGF binding protein (IGFBP)− 1 [977, 978], IGFBP- 2 and/or IGFBP- 3 [558] increase in older adults, further reducing free IGF- 1 levels. Age-related changes in circulating IGF- 1 [109, 131, 263, 264, 509, 510, 561, 562, 658, 788, 948] have been causally linked to the development of aging phenotypes in multiple organ systems, including the brain and the vasculature [263, 573, 574]. Importantly, decreases in circulating levels of IGF- 1 [264] may contribute to key aspects of both brain aging, including synaptic dysregulation [574, 955] and astrocyte dysfunction [98, 872, 979] and vascular aging, including, endothelial dysfunction [109, 980], impaired autoregulation of cerebral blood flow [788], pathological vascular remodeling [131, 658], atherogenesis [695, 697, 699], impaired cellular oxidative stress resilience [509, 714], impaired angiogenic processes and microvascular rarefaction (Fig. 10) [264, 981]. Low circulating IGF- 1 levels correlate with cognitive decline and likely play a role in the pathogenesis of dementia [573, 792, 982–985]. Thus, future mechanistic studies should further interrogate experimentally the role of circulating IGF- 1 in mediation of the vasoprotective and neuroprotective effects of young blood (e.g., using animal models with cell-specific knockdown of IGF- 1R as parabionts).

### FGF21

FGF21 is a member of the fibroblast growth factor family and is primarily secreted by the liver, adipose tissue, and skeletal muscle [986, 987]. FGF21 plays a crucial role in metabolic regulation, including enhancing glucose tolerance, improving insulin sensitivity, regulating lipid metabolism, and protecting cells through its antioxidant, anti-inflammatory, and anti-apoptotic properties. The protective effects of FGF21 are mediated through its binding to receptors such as FGFR1 and FGFR2, which are expressed in complexes with the co-receptor beta-klotho [987]. These receptor complexes activate several downstream signaling pathways, including PI3 K/Akt and AMPK/SIRT1 [988]. Importantly, the age-related chronic diseases, such as diabetes and obesity, lead to FGF21 resistance, limiting its ability to confer its protective effects [987, 989, 990].

The vasculoprotective action of FGF21 has been well demonstrated in preclinical models of aging, atherosclerosis, ischemic stroke and traumatic brain injury (TBI). In a mouse model of ischemic stroke, treatment with plasma from healthy mouse donors ameliorated stroke-induced BBB disruption, neuroinflammation, and neurological dysfunction through FGF21 [760]. Systemic administration of FGF21 alone restored BBB integrity, reduced neuroinflammation, protected white matter, and reduced infarct volume in the mouse model of stroke [991, 992]. These benefits were observed even when FGF21 was administered a week after stroke induction, highlighting its high therapeutic potential [993]. Similarly, in the TBI models, FGF21 restored BBB function, reduced neuroinflammation, and improved neurological recovery [994]. Moreover, FGF21 also exerts anti-atherogenic effects, partly mediated by increased adiponectin secretion [995–999]. FGF21 also exerts multifaceted anti-aging effects, protecting endothelial and vascular smooth muscle cells by reducing oxidative stress, improving mitochondrial function, restoring NO signaling, and preventing apoptosis under conditions of high glucose stress [1000] and angiotensin II-induced oxidative stress [999]. Furthermore, FGF21 has been shown to promote angiogenesis and improve endothelial function, particularly in models of cerebrovascular injury [760, 991–993, 999].

Although FGF21 expression in the brain is relatively low, FGF21 easily crosses the BBB, exerting protective effects on various cell types within the CNS [1001, 1002]. Preclinical studies have demonstrated that systemic FGF21 administration exerts neuroprotective effects in rodent models of CNS injury and cognitive impairment [991, 992]. The neuroprotective properties of FGF21 have also been explored in models of cognitive impairment induced by metabolic stress, such as high-fat diets [812, 1003, 1004]. Interestingly, reduced levels of FGF21 have been observed in patients with AD [1005] as well as in mouse models of AD [1005]. Preclinical studies suggest that FGF21 treatment in these models improves mitochondrial function, enhances neuronal survival and plasticity, reduces inflammation and tau pathology, protects against neuronal loss in the cortex and hippocampus, and preserves hippocampus-dependent learning and memory [1006]. Additionally, in a murine model of spinal cord injury, FGF21 treatment reduced glial scarring, promoted axonal regeneration, prevented neuronal loss, and improved locomotor functions [1007]. Similarly, in a traumatic brain injury model, intraventricular administration of FGF21 enhanced hippocampal neurogenesis, improved neuronal architecture, and positively influenced cognitive outcomes [1008]. Given the existing evidence, it is of particular interest to investigate how FGF21 could modulate age-related changes in cerebrovascular and brain function in aged organisms without underlying stroke or CNS injury. Further studies should also focus on developing therapeutic approaches to counteract FGF21 resistance in aging, thereby maximizing the benefits of FGF21 for cerebrovascular and cognitive health.

#### Angiotensin II and the renin-angiotensin system

Angiotensin II (Ang-II) is a peptide hormone converted from liver-derived angiotensinogen in a sequential conversion angiotensin-converting enzyme (ACE). Ang-II is the main effector of the renin–angiotensin–aldosterone system (RAAS). There are two distinct receptors activated by angiotensin II: AT_1_ and AT_2_. Activation of AT_1_ receptors leads to sodium and water retention, vasoconstriction, increased blood pressure, inflammation, oxidative stress, and fibrosis, whereas activation of AT_2_R leads to opposite outcomes [1009]. RAAS plays a crucial role in cardiovascular aging. Transgenic AT_1a_R-deficient mice are characterized by prolonged lifespan, decreased cardiac fibrosis and vascular damage, and decreased oxidative stress in cardiomyocytes and blood vessels [1010]. Aged AT_1a_R-deficient mice are protected from ROS-mediated damage in the cerebral circulation [1011]. Mice chronically treated with enalapril, an ACE inhibitor, showed reduced oxidative stress, cardiovascular and renal damage. Ang-II promotes pathological cardiovascular changes, including vascular and cardiac hypertrophy, which can be prevented by inhibiting Ang-II synthesis [1012, 1013]. Inhibition of RAAS is associated with decreased mitochondrial production of ROS, DNA damage, cardiac and vascular fibrosis, cellular senescence, and increased levels of NOS in vasculature [1014–1019]. Although the overall activity of RAAS declines with age, studies in humans show that aged individuals are more susceptible to detrimental effects of circulating angiotensin II [1020]. Besides the systematic RAAS, several organs, such as kidney, heart, and brain, have their local RAAS. A study of mouse aortas revealed age-related changes in vascular RAAS. With age, the AT_1_R-positive area and expression of Ang-II and ACE increase, whereas the expression of ACE2 and AT_2_R decreases with age [1021]. Moreover, the activity of ACE and ACE2 decrease in aged humans [1022]. ACE2 is an enzyme that converts Ang-II to Ang 1–7, a vasoprotective heptapeptide that mediates vasodilation and anti-inflammatory responses. This disbalance between detrimental ACE-Ang-II-AT_1_R axis and beneficial ACE-Ang-II-AT_2_R and ACE2-Ang- 1–7-MasR axes contributes to age-related cardiovascular pathologies [1016, 1023]. Recent studies show that inhibition of RAAS provides beneficial cognitive effects, manifested by improved cognitive function and reduced risk of cognitive impairment and dementia [1024].

### Osteokines

The skeletal system, traditionally viewed as crucial for providing structural support and facilitating body movement, has also been recognized for its role in regulating various physiological processes through the secretion of endocrine factors known as osteokines [1025]. These bone-derived factors are secreted predominantly by osteoblasts, osteoclasts, and osteocytes. Osteokines, including osteocalcin, fibroblast growth factor 23, lipocalin- 2, receptor activator of nuclear factor kappa beta (RANKL), sclerostatin, and proinflammatory cytokines, play important roles in glucose metabolism, energy regulation, vascular homeostasis, and cognition. However, with aging, systemic composition of osteokines changes significantly [1026], which is linked to the age-related diseases such as cardiovascular disease and cognitive decline [1027–1029]. Understanding the intricate relationship between these osteokines and brain and vascular health may provide new insights into how skeletal system factors influence systemic aging processes [1025].

#### Osteocalcin

Osteocalcin (OCN) is a pleiotropic hormone produced and secreted primarily by osteoblasts [1030]. It plays a multifaceted role in glucose metabolism, testosterone production, exercise adaptation, and neurodevelopment [1030]. Circulating levels of OCN decline with age in a sex-specific manner. In women, OCN levels decrease progressively over time, while men experience a more pronounced decline in midlife [1031, 1032]. Recent research highlights osteocalcin’s role in young blood-mediated rejuvenation of cognitive function [1033]. Upon crossing the BBB, osteocalcin activates Gpr158 receptors on CA3 hippocampal neurons, improving hippocampus-dependent memory [1033]. Animal studies demonstrated that osteocalcin-depleted young plasma loses its cognitive rejuvenating effects, while systemic administration of osteocalcin improves spatial learning and reduces anxiety-like behaviors in aged mice [1033]. Both clinical and preclinical studies indicate that levels of circulating osteocalcin positively correlate with cognitive functions, with significantly lower OCN levels observed in patients with dementia [1034–1037]. Moreover, systemic treatments with osteocalcin improved cognitive outcomes also in a mouse model of AD [1038]. While the specific effects of OCN on the vasculature are less understood, some evidence suggest a negative correlation between OCN levels and atherosclerosis, the longitudinal studies have yet to establish clear causality [1028]. In vitro experiments indicate that OCN may exert vasoprotective effects by increasing endothelial nitric oxide synthase expression and protecting against toxin-induced endoplasmic reticulum stress and apoptosis [1027]. Further research is essential to better understand its effects on cerebrovascular health.

#### Fibroblast Growth Factor 23 (FGF23)

Another bone-derived factor implicated in the systemic regulation of aging processes is FGF23. Primarily involved in phosphorus homeostasis, vitamin D and glucose metabolism, and neurogenesis, FGF23 exerts its effects by binding to one of four FGF receptors (FGFR1–FGFR4). In most tissues, the presence of the co-receptor alpha-klotho is essential for FGF23’s biological activity [1039]. Although circulating FGF23 levels increase with age, alpha-klotho expression decreases across organs, such as the kidneys, vasculature, and brain, resulting in FGF23 resistance, which contributes to the pathogenesis of many age-associated diseases [1039, 1040].

Clinical studies have shown an independent correlation between elevated circulating FGF23 levels and increased cardiovascular risks, including hypertension, atherosclerosis, and stroke [1041–1043]. For instance, higher FGF23 levels are positively correlated with hypertension and increased systolic blood pressures in community-dwelling older adults [1041]. Additionally, elevated FGF23 levels have been linked to increased white matter hyperintensity volumes, markers of cerebrovascular damage [1044]. Preclinical studies further indicate that FGF23 induces a phenotypic shift in vascular smooth muscle cells, leading to vascular thickening, stiffening, and calcification [1040]. In vitro experiments demonstrate that FGF23 promotes vascular calcification and arterial stiffness, exacerbating cardiovascular pathologies such as intracranial arteriosclerosis and atherosclerosis [1045, 1046]. These vascular changes increase the risk of ischemic damage to the brain, contributing to cognitive decline and neurovascular diseases, particularly in aging populations.

The role of FGF23 in cognitive decline is complex. A large study involving 1537 patients demonstrated that elevated FGF23 levels were associated with an increased risk of dementia and AD, suggesting a possible link between FGF23 and neurodegeneration [1047]. However, other studies have reported no significant association between FGF23 levels and cognitive function or the incidence of cognitive impairment [1048, 1049]. In mice, overexpression of FGF23 led to poorer cognitive performance, particularly in spatial learning tasks [1050]. Interestingly, this FGF23-induced cognitive impairment was alleviated when mice were fed a high-phosphorus diet, underscoring the role of FGF23-mediated phosphorus metabolism in cognitive health [1050]. Additionally, research suggests that alpha-klotho may mediate some of the beneficial effects of FGF23 on neurons. In the presence of klotho, FGF23 can exert protective effects on neurons and support neurogenesis [1051].

Elevated FGF23 levels have also been associated with an increased risk of silent brain infarcts and embolic infarcts, both of which are associated with cognitive decline [1045, 1052]. These silent infarcts contribute to VCID and exacerbate neurodegenerative processes in the brain. These findings highlight the need for further research into FGF23’s role in brain aging and its potential as a therapeutic target in VCID. Given its systemic effects on both cardiovascular and nervous systems, FGF23 appears to play a dual role in brain aging. Future studies are essential to clarify whether FGF23 directly drives cognitive decline and vascular aging or if its elevation reflects underlying metabolic or vascular pathologies.

##### LCN- 2

Lipocalin- 2 (LCN- 2) is one of the most recently identified osteokines, predominantly secreted by osteoblasts and adipocytes [1027]. It plays diverse roles in regulating iron homeostasis, energy metabolism, cell differentiation, apoptosis, and inflammation [1053]. Under physiological conditions, brain levels of LCN- 2 are low but essential for neurogenesis and cognitive function [1054, 1055]. An age-related brain disorders, such as mild cognitive impairment, vascular dementia, and AD, LCN- 2 levels are significantly higher [1054, 1055]. LCN- 2 can cross the BBB, and its brain levels are largely derived from circulation, as the local production within the neurovascular unit is minimal [1056]. In animal models of ischemia–reperfusion, increased LCN- 2 levels were shown to exacerbate neuroinflammation and BBB permeability, while reducing LCN- 2 levels, either genetically or pharmacologically, mitigated brain damage and improved neurological outcomes [1053, 1057–1059]. Mechanistically, LCN- 2 has been linked to NLRP3 inflammasome activation, reduced claudin- 5 expression, and increased neuroinflammation, highlighting its detrimental role in cerebrovascular and neuronal health [1060].

Interestingly, elevated CSF levels of LCN- 2 have been proposed as a biomarker for vascular dementia, distinguishing it from AD [1061]. High circulating LCN- 2 levels are also associated with increased cardiovascular disease risk, including coronary artery disease and atherosclerosis [1062–1064]. Preclinical studies have further demonstrated that LCN- 2 promotes endothelial activation and accelerate atherosclerotic plaque formation by promoting monocyte-macrophage infiltration into lesions [1064, 1065]. Furthermore, within atherosclerotic lesions LCN- 2 expression is elevated, and genetic depletion of LCN- 2 has been shown to reduce the necrotic core of these lesion [1066]. These observations suggest that LCN- 2 may be a critical link between bone-derived factors and systemic aging, particularly affecting brain and vascular health.

In conclusion, emerging evidence on the age-related disruption in paracrine crosstalk between bones, vasculature, and the brain, underscores the impact of osteokines in mediating systemic aging and the importance for further research. Understanding the contributions of osteocalcin, FGF23, and LCN- 2 to cerebrovascular aging and cognitive decline may provide novel therapeutic targets for combating age-related diseases.

### Myokines

Myokines are cytokines and peptides secreted by muscle cells in response to contraction and other physiological stimuli, representing a unique link between skeletal muscle activity and systemic health. These signaling molecules play a crucial role in inter-organ communication, impacting metabolism, inflammation, and even neurocognitive function. Age-related changes in muscle composition and function, including the progression to sarcopenia, lead to altered myokine production, potentially contributing to the broader decline in cardiovascular, metabolic, and cognitive health observed with aging. These shifts in myokine profiles not only reflect reduced muscle mass and quality but also disrupt the beneficial signaling pathways that myokines mediate between muscle and other organs. Consequently, this altered myokine production may exacerbate age-related risks for diseases such as cardiovascular conditions, insulin resistance, and neurodegenerative disorders.

Importantly, the muscle secretome undergoes significant changes during and after physical activity [1067], a phenomenon crucial to understanding how lifestyle factors like exercise can influence health outcomes in aging. Both endurance and resistance training have demonstrated beneficial effects across organ systems, particularly in preventing or ameliorating organismal aging and improving cardiovascular and cerebrovascular health. These improvements stem not only from enhanced tissue perfusion, oxygenation, and cellular metabolism but also from the endocrine actions of muscle-derived factors, collectively termed “exerkines.” Exerkines include various types of myokines, such as cytokines and growth factors (e.g., BDNF, irisin, cathepsin B, and IGF- 1), as well as small signaling molecules, metabolites, microRNAs, and extracellular vesicles (exosomes). These factors serve as systemic messengers, mediating the positive effects of physical activity on aging-related processes. Exercise-induced increases in myokines are critical for signaling pathways that support cellular resilience, metabolic function, and vascular health. Myokines such as BDNF, IGF- 1, and irisin have been shown to cross the BBB, where they contribute to neurogenesis, enhance cognitive function, and protect against neurodegeneration. In parallel, myokines support cerebrovascular health by promoting endothelial function and reducing inflammation. Thus, physical activity-induced changes in myokine secretion represent a powerful intervention in modulating the aging phenotype, potentially counteracting the deleterious effects of age-related muscle decline on whole-body health.

Parabiosis studies have provided significant insights into the rejuvenative potential of myokines and exerkines on aging tissues. Physical activity, well-established for its health-promoting effects, increases the secretion of beneficial myokines, suggesting that enhanced myokine levels from the younger or more physically active partner in a parabiosis setup could contribute to systemic rejuvenation. Several myokines, including BDNF, irisin, and follistatin-like protein 1 (FSTL1), have shown potential in promoting neuroprotection, and vascular health. While definitive conclusions on myokine-mediated effects in parabiosis are still limited, the preliminary findings underscore the relevance of muscle-secreted factors in systemic rejuvenation. Future mechanistic studies that combine parabiosis with exercise interventions may reveal critical insights into the mechanisms by which myokines exert regenerative effects, supporting their potential as therapeutic targets for counteracting age-related functional decline across various tissues.

### BDNF

BDNF is a crucial myokine with significant roles in neuroplasticity, cognitive function, and cellular resilience. It is well-recognized for its contributions to neural repair and plasticity, as well as for its neuroprotective effects that extend to the central and peripheral nervous systems. BDNF levels are observed to peak in individuals during their thirties, with a gradual decline through middle and older ages [1068]. This age-related reduction in BDNF, coupled with diminished neurotrophic support, is associated with cognitive decline, increased neurodegenerative risk, and muscle weakness, all hallmarks of aging [1069–1071]. Exercise acts as a potent inducer of BDNF production, temporarily elevating its circulating levels. Following long-term exercise training, resting BDNF decline [1072–1074]. This seemingly paradoxical decrease is thought to result from an exercise-induced increase in BDNF binding sites within muscles and nerves, which serves to address and repair micro-injuries from the oxidative and mechanical stress induced by physical activity [1075, 1076]. As a form of hormesis—a beneficial effect stemming from a low-dose response to a stressor—exercise-induced BDNF enhances resilience against stress and fosters tissue repair [1077]. In addition to its well-known roles in brain health, BDNF promotes cardiovascular health by improving endothelial function and reducing inflammatory responses [1078, 1079]. Its ability to cross the BBB [1080] enables BDNF to act directly within the CNS, aiding in neurogenesis and cognitive maintenance. The age-related decline in BDNF emphasizes the importance of sustained physical activity across the lifespan, as exercise emerges as a natural intervention to stimulate BDNF production and, in turn, support healthy neurovascular and cognitive aging.

#### Irisin

Irisin is a polypeptide myokine derived from the cleavage of the fibronectin type III domain-containing protein 5 (FNDC5), though the enzyme responsible for this proteolytic process remains unidentified [1081]. Irisin is synthesized in skeletal muscle, and its plasma levels are generally low in sedentary individuals (around 3.6 ng/mL) but rise significantly following physical exercise [1082]. This increase suggests that irisin plays a role in mediating some of the systemic benefits of physical activity, particularly on the central nervous system and vascular health. The beneficial effects of irisin on brain health are partly mediated through its relationship with BDNF. In mice, the overexpression of irisin has been shown to upregulate hippocampal BDNF levels, enhancing neuroplasticity and possibly offering protection against cognitive decline. In humans, circulating irisin correlates positively with cognitive performance [1083]. The role of irisin in aging is complex; while some studies have observed increased irisin levels in plasma and CSF in healthy centenarians, suggesting a possible protective effect [1084, 1085], other studies have reported lower irisin levels in older populations, highlighting the need for further investigation to clarify these discrepancies [1086].

In terms of cardiovascular health, irisin’s role remains under active exploration. Evidence from several studies indicates that irisin may have vasoprotective effects, including enhanced endothelium-dependent vasodilation [1087], reduced vascular calcification, decreased vascular inflammation and anti-atherogenic effects [1087]. These effects are primarily attributed to irisin’s ability to promote endothelial cell proliferation, enhance autophagy in VSMCs, and mitigate vascular inflammation [1087–1091]. The link between irisin levels and cardiovascular disease (CVD) remains inconclusive; while some studies report a protective association, others show no correlation or even a negative association with cardiovascular health [1087, 1092, 1093]. These mixed findings underscore the need for more precise methodologies to measure irisin accurately in plasma and to better understand its effects under different biological conditions.

#### Cathepsin B

Another exerkine of interest is cathepsin B (CTSB), a cysteine protease involved in regulating multiple biological processes such as angiogenesis, neurogenesis, tumor proliferation, and immune responsivity [1094]. Studies show that CTSB levels increase in skeletal muscle following exercise, leading to an upregulation of BDNF expression in neurons [1095]. Importantly, this exercise-induced rise in circulating CTSB has been observed in rodents, non-human primates, and humans, where it correlates positively with cognitive performance, particularly in tests measuring memory and learning [1095, 1096]. However, while the exercise-associated role of CTSB generally appears beneficial, its contributions to overall health, particularly in aging and disease, present a more complex picture [1094].

Despite CTSB’s positive association with exercise and cognition, it is also implicated in neurodegenerative disease pathogenesis. While initial studies highlighted potential neuroprotective effects of CTSB, such as reducing amyloid-beta levels and improving cognition in Alzheimer’s disease models, recent evidence suggests its role may extend to promoting neurodegeneration [1097, 1098]. Four key mechanisms underpin CTSB’s detrimental effects in neurodegenerative disease: (1) promoting inflammation by shifting myeloid cells to a pro-inflammatory phenotype, (2) degrading the extracellular matrix, (3) increasing mitochondrial dysfunction and oxidative stress, and (4) driving pyroptosis and apoptosis, leading to neuronal damage [1099]. Notably, elevated CTSB levels are found in plasma, serum, and CSF in individuals with neurodegenerative diseases, including AD, where higher CTSB levels correlate with worse cognitive function as measured by the Mini-Mental State Examination (MMSE) [1097, 1100]. Additionally, increased CTSB expression is documented in AD brains and transgenic rodent models, with CTSB downregulation in these models showing symptom improvements [1101].

CTSB’s role in cerebrovascular disease is similarly complex, particularly concerning atherosclerosis and cerebral aneurysms. In atherosclerosis, CTSB contributes to arterial instability and stiffness by degrading the extracellular matrix within arterial walls, promoting macrophage apoptosis, increasing neuroinflammation, inducing pyroptosis in vascular smooth muscle cells, and promoting foam cell formation [1101–1104]. Elevated CTSB levels in coronary heart disease patients correlate with increased cardiovascular events and all-cause mortality [1104]. Additionally, CTSB has been linked to cerebral aneurysm formation, with research highlighting its role in vascular remodeling and aneurysm development [1105].

With age, serum activity and circulating levels of CTSB increase, potentially exacerbating the pro-inflammatory and oxidative environment associated with aging [1106, 1107]. Importantly, CTSB deletion in aged wild-type mice has been shown to reduce oxidative stress, inflammation, and cognitive decline, indicating that CTSB could be a viable target for anti-aging and cerebrovascular protective therapies [1094]. In summary, while CTSB appears beneficial in the context of exercise, promoting neurogenesis and cognitive improvements, it may also drive degenerative processes in neurovascular aging and neurodegenerative disease. Further research is essential to determine the conditions under which CTSB could serve as a therapeutic target versus a contributor to disease progression.

##### FSTL1

FSTL1 is a myokine broadly expressed throughout the body, secreted not only by skeletal muscles but also by cardiac tissue and vascular structures [1108, 1109]. Stressors such as physical exercise, muscle injury, cardiac ischemia–reperfusion injury, and pressure overload substantially elevate FSTL1 secretion from affected tissues as a protective response [1110]. In cases of cardiovascular and cerebrovascular disease, FSTL1 levels are notably increased, underscoring its potential as a biomarker for these pathologies [1111, 1112]. With age, circulating FSTL1 levels show a modest increase, possibly reflecting the prevalence of cardiovascular disease and an overall pro-inflammatory environment in older adults [1113]. The secretion of FSTL1 during stress is beneficial, as this glycoprotein offers cardio- and vasculoprotective effects. In the cardiovascular system, FSTL1 has been shown to reduce cardiomyocyte apoptosis, cardiac fibrosis, and ischemia–reperfusion-induced infarct size, while enhancing endothelial cell angiogenesis and normalizing cardiac metabolism [1110, 1114–1117]. Its role in revascularization and reperfusion extends to skeletal muscle injury, arterial damage, and pulmonary hypertension, where it supports tissue recovery through its impact on endothelial function [1118–1120]. Endothelial cells also secrete FSTL1, underscoring its importance for endothelial health. Downregulation of FSTL1 in the endothelium is associated with adverse consequences, including cardiac hypertrophy, heightened pulmonary resistance, and vascular fibrosis [1121]. In models of atherosclerosis, both in vitro and in vivo, FSTL1 has demonstrated protective effects on endothelial cells, further supporting its critical role in maintaining vascular integrity [1122]. Although the effects of FSTL1 on brain and cognition are not yet well-established, its systemic influence through vasculoprotective and anti-inflammatory mechanisms may indirectly support cerebrovascular health, which is crucial for cognitive function.

### Sex hormones

Sex hormones, including testosterone and estrogen, play pivotal roles in regulating various physiological processes throughout life, including muscle mass, bone density, cardiovascular health, and cognitive function [1123]. As aging progresses, the decline in these hormones contributes significantly to the pathophysiology of aging-related diseases, especially in the brain and vascular systems [1124]. This section delves into the roles of testosterone and estrogen in brain and vascular aging, highlighting the impact of their decline and potential therapeutic implications.

#### Testosterone and vascular aging

Testosterone, the primary male sex hormone, plays a crucial role in maintaining muscle mass, strength, and cardiovascular health. Circulating testosterone levels peak in early adulthood and progressively decline with age [1125, 1126]. Importantly, heterochronic parabiosis studies indicate that decreased testosterone levels are causally associated with the age-related loss of muscle mass and function [458]. In addition to muscle atrophy, testosterone plays a multifaceted role in maintaining cardiovascular health. Age-related testosterone decline has been linked to increased cardiovascular risks, such as hypertension, atherosclerosis, and coronary heart disease [1127–1130]. Low testosterone levels are associated with endothelial dysfunction [1131]. Furthermore, testosterone has anti-inflammatory effects on the vascular system, reducing oxidative stress and improving NO production, which enhances endothelial function [1131–1137].

Testosterone also influences lipid metabolism, with low testosterone levels linked to unfavorable lipid profiles, such as elevated LDL cholesterol, triglycerides, and total cholesterol, as well as reduced high-density lipoprotein (HDL) cholesterol, all of which elevate the risk of atherosclerosis [1138, 1139]. Clinical studies suggest that testosterone replacement therapy (TRT) can mitigate many of these cardiovascular risks by improving lipid profiles, enhancing endothelial function, and reducing arterial stiffness [1140–1142]. Importantly, TRT has generally been well tolerated, with a relatively low incidence of adverse effects. Recent studies indicate that the risk of severe adverse events, such as thrombosis or prostate cancer, is lower than previously thought [1143, 1144]. However, given testosterone’s broad biological effects, thorough patient evaluation and careful monitoring are essential when considering TRT.

#### Testosterone and brain aging

In the brain, testosterone exerts neuroprotective effects by promoting neurogenesis, enhancing synaptic plasticity, and supporting neuronal survival [1145–1149]. Testosterone and its metabolite, dihydrotestosterone (DHT), can modulate neurotransmitter release and receptor sensitivity, affecting cognitive function and mood [1150, 1151]. In men, the age-related decline in testosterone is associated with an increased risk of neurodegenerative diseases, such as AD [1152–1154], as well as cognitive decline and mood disorders, including depression and anxiety [1155–1157].

Testosterone is known to promote the survival of neurons by enhancing mitochondrial function and reducing oxidative stress and apoptosis [1158, 1159]. Low testosterone levels have been linked to increased oxidative stress and inflammation in the brain, contributing to cognitive decline [1160, 1161]. Additionally, testosterone has been shown to exert anti-amyloidogenic effects by reducing the production and deposition of amyloid-beta plaques, which are hallmarks of AD [1154]. Furthermore, testosterone plays a role in modulating the BBB [1162]. Studies suggest that declining testosterone levels may contribute to BBB dysfunction, exacerbating neuroinflammation, and accelerating neurodegenerative processes [1161]. In women, androgens like testosterone are produced in smaller amounts, but they still contribute to brain function [1163]. Emerging evidence suggests that postmenopausal women with low testosterone levels may experience an increased risk of cognitive decline, although further research is needed to fully understand the gender-specific effects of testosterone on brain aging [1164, 1165].

#### Estrogen and vascular aging

Estrogen, the primary female sex hormone, plays a key role in maintaining cardiovascular health and protecting against vascular aging [1166]. Before menopause, women tend to have a lower risk of cardiovascular diseases than men, largely due to the protective effects of estrogen. Estrogen promotes vasodilation by stimulating the production of NO in endothelial cells, improving blood flow and reducing blood pressure [1167]. Additionally, estrogen reduces oxidative stress and inflammation within the vasculature, protecting against endothelial dysfunction and atherosclerosis [1168, 1169]. As women age and transition into menopause, estrogen levels significantly decline, resulting in a loss of this vascular protection [1170]. Postmenopausal women experience an increased risk of hypertension, dyslipidemia, and atherosclerosis, contributing to a higher incidence of cardiovascular diseases, such as coronary artery disease and stroke, compared to premenopausal women [1170, 1171]. Studies have shown that hormone replacement therapy (HRT) with estrogen can help mitigate some of these risks by restoring endothelial function, reducing arterial stiffness, and improving lipid profiles [1172–1174]. However, the use of HRT is controversial, as some studies have associated estrogen therapy with an increased risk of thromboembolism, breast cancer, and other complications [1175, 1176]. The timing of HRT initiation appears to be crucial, with the “timing hypothesis,” suggesting that early initiation of estrogen therapy may provide cardiovascular benefits, whereas delayed therapy may be less effective or even harmful [1177, 1178].

#### Estrogen and brain aging

Estrogen is also critical for brain function, influencing cognitive processes, mood regulation, and neuroprotection. In the brain, estrogen acts on estrogen receptors (ERα and ERβ) that are widely expressed in regions involved in learning and memory, such as the hippocampus and prefrontal cortex [1179, 1180]. Estrogen enhances synaptic plasticity, increases the expression of BDNF, and modulates neurotransmitter systems, including acetylcholine, dopamine, and serotonin [1181–1183]. These actions help maintain cognitive function and mood stability throughout life. The decline in estrogen during menopause is associated with an increased risk of cognitive decline, memory impairments, and neurodegenerative diseases, particularly AD [1184–1186]. Estrogen has been shown to reduce the accumulation of amyloid-beta plaques and tau tangles in the brain, key pathological features of AD [1187, 1188]. Additionally, estrogen exerts anti-inflammatory and antioxidant effects, reducing neuroinflammation and oxidative damage, both of which are linked to age-related cognitive decline [1189, 1190]. HRT has been studied as a potential intervention for preventing cognitive decline in postmenopausal women [1191, 1192]. Some studies suggest that early initiation of HRT may help preserve cognitive function and reduce the risk of AD, particularly in women with a genetic predisposition to the disease [1193, 1194]. However, like with cardiovascular health, the timing of HRT initiation is crucial. The “critical window hypothesis” proposes that initiating HRT shortly after menopause may confer cognitive benefits, whereas delayed HRT initiation may be ineffective or harmful [1177].

In conclusion, sex hormones such as testosterone and estrogen play significant roles in regulating both vascular and brain aging. The age-related decline in these hormones contributes to increased risks of cardiovascular diseases, neurodegenerative diseases, and cognitive decline. While testosterone and estrogen are traditionally viewed as male and female hormones, respectively, both hormones play important roles in both sexes. For example, estrogen has cardioprotective and neuroprotective effects in men, and testosterone is necessary for optimal cognitive function in women. The balance of these hormones, rather than absolute levels, is likely crucial for health across both sexes. Sex-specific differences in hormone decline and aging processes are evident, with men experiencing a more gradual decline in testosterone, while women undergo an abrupt decline in estrogen during menopause. These hormonal changes contribute to the sex differences observed in age-related diseases, including cardiovascular and neurodegenerative diseases. Further research is needed to fully understand the sex-specific effects of hormone decline and to optimize therapeutic strategies such as hormone replacement therapies in both men and women.

### Cytokines, chemokines, and other inflammatory factors

As organisms age, chronic low-grade inflammation, often referred to as “inflammaging,” plays a key role in driving age-related diseases and dysfunctions [1195]. This persistent inflammation is characterized by elevated levels of various pro-inflammatory cytokines, chemokines, and other inflammatory mediators in circulation [1195]. These molecules promote systemic and tissue-specific inflammation, contributing to age-associated pathologies, including cardiovascular diseases, neurodegenerative disorders, metabolic syndrome, and impaired tissue repair [439]. Among the most prominent inflammatory mediators are cytokines like interleukin- 6 (IL- 6), interleukin- 1β (IL- 1β), TNF-α, and chemokines such as CCL11 and CCL5 [1196]. Importantly, many of these factors are secreted by senescent cells as part of the senescence-associated secretory phenotype (SASP), which plays a central role in propagating chronic inflammation in aging tissues. This section will explore the role of key proinflammatory factors, focusing on their contribution to vascular and brain aging [434], as well as their potential as therapeutic targets.

#### SASP factors and other inflammatory cytokines

SASP factors, which include a variety of proinflammatory cytokines (e.g., IL- 6, IL- 1β, TNF-α), chemokines, and matrix-degrading enzymes, are secreted by senescent cells and play a central role in chronic inflammation and tissue dysfunction associated with aging [434]. Senescent cells accumulate in various tissues with age and, through SASP, promote persistent, low-grade local and systemic inflammation [1197]. These inflammatory factors, particularly when elevated in circulation over long periods, can contribute to a wide array of age-related diseases, including cardiovascular disease, neurodegeneration, and metabolic disorders [1197, 1198]. Chronic inflammation driven by these cytokines can lead to increased oxidative stress and mitochondrial dysfunction, further exacerbating the aging process. Targeting these inflammatory mediators, either by inhibiting specific cytokines or reducing the burden of senescent cells, holds promise for mitigating the detrimental effects of inflammaging and improving healthspan [1199]. Several clinical trials involving cytokine inhibitors have shown potential benefits, and further studies are needed to determine the long-term efficacy of these interventions in combating age-related inflammation [1199–1201].

##### Interleukin- 6 (IL- 6)

IL- 6 is a prominent cytokine that plays a central role in age-related inflammation and is consistently elevated in aging populations. Elevated circulating IL- 6 is strongly associated with the risk of cardiovascular disease and mortality in older adults [1202]. Studies show that IL- 6 is a reliable predictor of adverse cardiovascular outcomes [1203]. In the context of brain aging, IL- 6 has been implicated in the pathogenesis of neurodegenerative diseases like AD and VCID [1204, 1205]. Elevated IL- 6 levels can induce neuroinflammation, contribute to synaptic dysfunction, and promote Aβ plaque formation and tau hyperphosphorylation [1206, 1207]. Additionally, IL- 6 has been shown to disrupt the BBB, which exacerbates neurodegeneration [1208, 1209]. Increased IL- 6 has also been linked to cognitive decline, particularly in VCID [433, 434].

##### Interleukin- 1β (IL- 1β)

Like IL- 6, IL- 1β is a critical mediator of inflammation and is elevated in circulation as humans age [1210]. Elevated IL- 1β has been implicated in both cardiovascular disease and neurodegenerative processes [1211–1213]. In cardiovascular aging, IL- 1β contributes to atherosclerosis and vascular inflammation, which can lead to increased risks of stroke and other cardiovascular events [1214]. In the brain, IL- 1β promotes neuroinflammation by activating microglia and astrocytes, which can exacerbate neurodegenerative processes [1215, 1216]. In AD, IL- 1β has been shown to promote the formation of amyloid plaques and tau tangles, further driving cognitive decline [1216]. IL- 1β also disrupts cerebrovascular integrity by impairing endothelial function and increasing BBB permeability, contributing to both AD and VCID [1217–1219]. Clinical studies have shown that targeting IL- 1β with the antibody canakinumab significantly reduces cardiovascular and inflammatory events, suggesting its potential role in reducing brain inflammation and cognitive decline [1220–1223].

##### Tumor necrosis factor α (TNF-α)

TNF-α is another major proinflammatory cytokine that rises in the bloodstream with age and contributes to the proinflammatory state characteristic of aging [426]. Elevated TNF-α levels have been correlated with poor cardiovascular outcomes, including increased risk of myocardial infarction (MI), stroke, and heart failure [1224–1226]. Additionally, TNF-α contributes to endothelial dysfunction by promoting oxidative stress, inflammation, and apoptosis within the vasculature [1227, 1228]. Suppression of TNF-α signaling has been shown to improve mitochondrial function and reduce ROS production, suggesting a therapeutic benefit in targeting this cytokine [1229]. In the brain, TNF-α plays a key role in neuroinflammation and neurodegeneration [1230–1232]. It is elevated in patients with AD, where it exacerbates synaptic dysfunction, promotes amyloid plaque formation, and contributes to neuronal death [1230, 1233]. TNF-α has also been implicated in the progression of VCID by contributing to cerebrovascular damage, increased BBB permeability, and disruption of cerebral blood flow [1219, 1234, 1235]. Preclinical studies show that inhibiting TNF-α can improve cognitive function and reduce neuroinflammation in animal models of AD and stroke, highlighting its potential as a therapeutic target for neurovascular diseases [1230, 1236].

##### Adhesion molecules (VCAM- 1 and ICAM- 1)

Soluble forms of adhesion molecules like VCAM- 1 and ICAM- 1 increase with age and contribute to vascular inflammation by promoting the adhesion and infiltration of immune cells into vascular walls [193, 1237, 1238]. In cardiovascular aging, elevated levels of these adhesion molecules are associated with atherosclerosis and other vascular diseases [1220, 1239]. Preclinical models have shown that blocking VCAM- 1 and ICAM- 1 can reduce vascular inflammation, remodeling, and plaque formation [1240]. Soluble VCAM- 1 and ICAM- 1 play important roles in brain aging. On top of age-related increases in circulating VCAM1, expression of its insoluble form increases in cerebrovasculature with aging, inducing proinflammatory and vascular remodeling-related transcriptomic shifts in VCAM1-positive brain endothelial cells [193]. In mechanistic studies, young mice treated with soluble VCAM- 1 activates microglia and impairs hippocampal neurogenesis, mimicking effects of aged plasma treatment [193]. Moreover, treatment with anti-sVCAM1 antibody improves hippocampal neurogenesis, ameliorates microgliosis, and enhances spatial learning [193]. Collectively, VCAM- 1 and ICAM- 1 contribute to cerebrovascular endothelial dysfunction, BBB disruption, neurovascular inflammation, and cognitive impairments, particularly in conditions such as AD and VCID [1241–1245].

##### GDF11

Growth differentiation factor- 11, also known as bone morphogenetic protein 11 or BMP- 11, is a member of the TGF-β superfamily member involved in the development of many organs [1246]. In recent proteomic studies GDF11 has been identified as a putative anti-geronic circulating factor that regulates aging of skeletal muscled [237] and heart [189, 1247], although the results were subsequently disputed [1248]. Findings from heterochronic parabiosis studies demonstrated that young blood-enriched GDF- 11 is critical for rejuvenation of skeletal muscles in aged parabiont mice [237]. GDF- 11 increases the number and functionality of the satellite cells in the skeletal muscle, leading to an increased number of myofibrils, improving physical performance in aged mice [237]. GDF- 11 was also suggested to contribute to young blood-mediated rejuvenation of the cardiac muscle, increasing the number of cardiomyocytes [189]. Subsequently, GDF- 11 was also shown to exert rejuvenating effects in the aged mouse brain [50, 809, 946].

GDF- 11 is expressed in many tissues (e.g., skeletal muscle, kidney, nervous system). As a cytokine from the TGF-β family, GDF11 signals through ALK5/TGFβR2 and pSMAD2/3, similar to TGF-β1. As it does not cross the BBB, it is proposed that GDF- 11 confer its rejuvenating effects in the brain via its effects on the cerebral microvascular endothelial cells [60]. GDF- 11 was reported to increase secretion of VEGF from cerebral endothelial cells, promoting local angiogenesis, neuronal activity, and neurogenesis in the hippocampus [176, 203]. GDF- 11 was reported to improve endothelial function and exert atheroprotective effects in apolipoprotein E-null mice [691]. In vitro studies revealed that peripheral blood endothelial progenitor cells treated with GDF- 11 were characterized by improved migration and tube formation without changes in proliferation and adhesion [1249]. Additionally, GDF- 11 improved the viability of human umbilical vein endothelial cells but not proliferation and migration [1250]. In the cerebral ischemia–reperfusion injury model, administration of GDF- 11 increased proliferation of ECs and endothelial expression of CD34 (endothelial cell differentiation marker) within the periinfarct tissue, leading to increased branching and density of blood vessels [771]. However, there are many observations that are inconsistent regarding the GDF- 11 as a key circulating anti-aging factor [1248]. First, methodological problems with the detection of GDF- 11 have been identified, which likely confounded the results of earlier studies on age-related changes in GDF- 11 and their role in regulation of aging processes [1248]. Further, exogenous administration of GDF- 11 induces cardiac and skeletal muscle dysfunction and wasting [1251, 1252].

##### GDF15

Growth differentiation factor- 15, also known as macrophage inhibitory cytokine- 1 (MIC- 1), is a member of the transforming growth factor-beta superfamily. GDF15 is a versatile cytokine involved in regulating immune and inflammatory responses, predominantly released in response to cellular stress [362]. Closely linked to the SASP, GDF15 contributes to the pro-inflammatory and pro-geronic environment created by senescent cells [424, 1253–1256]. Initially discovered in macrophages, GDF15 is also secreted by other cell types, including adipocytes, endothelial cells, and fibroblasts, particularly under conditions of oxidative stress, DNA damage, mitochondrial dysfunction, and resultant senescence [424, 1253–1256]. As aging progresses and senescent cell burden increases across various tissues, circulating GDF15 levels also rise with age [1257]. Elevated circulating GDF15 is observed in various pathological conditions, including cardiovascular disease, cancer, metabolic syndrome, and neurodegenerative diseases, making it an important biomarker for age-related diseases and overall mortality risk [1005, 1258, 1259].

GDF15 has emerged as a significant player in cardiovascular aging. Elevated circulating levels of GDF15 are associated with a range of cardiovascular conditions, including heart failure, myocardial infarction, and atherosclerosis. Studies have shown that GDF15 is a highly predictive biomarker of cardiovascular events and mortality in patients with heart disease and myocardial infarction [1260, 1261]. In older adults, GDF15 levels also correlate with frailty, increased morbidity, and reduced physical function [1262–1264]. These elevated levels are believed to reflect GDF15’s role in the cellular stress response. Sustained elevation of GDF15 has been also linked to poor prognosis and increased mortality in patients with cerebrovascular diseases [1265–1268]. While persistently high levels of GDF15 are indicative of chronic stress or inflammation and can contribute to adverse cardiovascular outcomes, emerging evidence suggests that GDF15 may also play a role in limiting acute inflammation in a context-dependent manner [1269–1274]. This context-dependent effect highlights the need for further research to unravel the multifaceted mechanisms of GDF15 in cardiovascular and cerebrovascular pathophysiology [1258].

In the central nervous system, GDF15 plays a complex role in neuroinflammation and neurodegeneration [1265]. Elevated GDF15 levels have been observed in patients with neurodegenerative and cerebrovascular diseases, including AD, PD, and dementia, where plasma concentrations of GDF15 correlate with disease progression and cognitive impairment [1005, 1265, 1275–1277]. Extensive research, including large-scale cohorts such as the Whitehall II study, the Atherosclerosis Risk in Communities (ARIC) study, and the UK Biobank, has consistently associated elevated GDF15 levels with a heightened risk of dementia [1278–1282]. However, its causal role in these diseases remains unproven [1283]. Given the complex and multifaceted effects of circulating GDF15 [1283–1285] in cerebrovascular and neurodegenerative diseases, further mechanistic studies are needed to elucidate how GDF15 influences cellular pathways and contributes to long-term brain health.

#### Eotaxin (CCL11)

Eotaxin, also known as CCL11, is a chemokine primarily involved in the recruitment of eosinophils to sites of inflammation. However, recent studies have identified CCL11 as a significant factor in the aging process, particularly in the context of neuroinflammation and cognitive decline [79]. Eotaxin levels are known to increase with age and are linked to various age-related conditions, including neurodegenerative diseases [1286, 1287], VCID [862, 1287], and general brain aging [861, 1288].

CCL11 has been shown to play a detrimental role in the brain, contributing to age-related cognitive decline [79, 1286]. Studies using heterochronic parabiosis have demonstrated that elevated levels of eotaxin in older animals can impair neurogenesis and cognitive function [79]. Similarly, administration of CCL11 to young mice resulted in a reduction in hippocampal neurogenesis and cognitive function, reinforcing the notion that CCL11 negatively affects brain plasticity and learning capacity [79]. Additionally, it has been demonstrated that young bone marrow transplant-induced rejuvenation of neurogenesis and synaptic plasticity is mediated by reduction of CCL11 [218]. The CCL11/CCR3 signaling axis has been suggested to promote neuroinflammation by attracting eosinophils into the brain, which could enhance the local inflammatory response, particularly in neurodegenerative diseases such as AD and VCID [861, 1289–1291]. Moreover, CCL11 induces microglia to produce ROS, resulting in neurotoxicity and neuronal loss [1292].

Blood derived chemokines, such as eotaxin, contribute to BBB dysfunction, a hallmark of both aging and neurodegenerative diseases [861, 1293–1295]. The breakdown of the BBB facilitates the infiltration of peripheral immune cells into the brain, further amplifying neuroinflammation [94, 1291]. In AD, elevated levels of eotaxin have been associated with the progression of the disease irrespectively of age [1296, 1297]. Elevated CSF levels of CCL11 and hippocampal expression of its receptor, CCR3, have been observed in mice models of AD [1298]. In APP/PS1 double-transgenic AD mice XX mice, downregulation of CCR3 rescued symptoms of AD, manifested by decreased amyloid beta production, tau hyperphosphorylation, neuroinflammation, ameliorated dendritic synaptic loss, and rescued impairments in spatial learning and memory [1298]. Moreover, the authors demonstrated that hippocampal cultures treated with CCL11 demonstrated increased tau phosphorylation and amyloid beta production [1298]. Lastly, another C–C motif chemokine, CCL2, has demonstrated even better prognostic properties in estimating cognitive decline in AD [1299].

Beyond its role in AD, eotaxin has been linked to cerebrovascular diseases such as VCID [862, 1287]. Inflammation plays a significant role in VCID, and chemokines like eotaxin exacerbate this process by promoting oxidative stress, senescence, endothelial dysfunction and neuroinflammation [1294, 1300]. The increase in CCL11 levels observed in older individuals may contribute to VCID by further disrupting cerebral blood flow and enhancing the pro-inflammatory state of the vasculature. Given the role of eotaxin in promoting neuroinflammation and cognitive decline, targeting CCL11 may offer therapeutic potential in aging and neurodegenerative diseases [1295]. Inhibiting CCL11 signaling or reducing its levels could potentially restore neurogenesis, improve cognitive function, and reduce neuroinflammatory responses. Some experimental therapies aimed at modulating the systemic environment to lower levels of pro-aging factors like CCL11 have shown promising results in preclinical models. Further research is needed to determine the efficacy and safety of these approaches in human subjects.

#### ActA

Activin A (ActA) is a member of the TGF-β superfamily, a group of proteins that regulate a wide range of biological processes, including cell proliferation, differentiation, and apoptosis. Activin A signals through type I and type II activin receptors, triggering the phosphorylation of SMAD2/3, which then translocate to the nucleus to regulate the transcription of target genes [1301]. Research into the role of activin A in aging has revealed both beneficial and detrimental effects, a phenomenon often explained by the concept of antagonistic pleiotropy, where genes or factors have positive effects early in life but negative consequences during aging. In early life, activin A plays an essential role in regulating normal physiological processes such as cell growth, development, and tissue repair. It is involved in the differentiation of various cell types, the regulation of reproductive functions, and the modulation of immune responses [1301]. However, with age, activin A’s role becomes more complex, as it has been implicated in the promotion of chronic inflammation, cardiovascular disease, neurodegeneration, and cancer [1302]. As organisms age, levels of Activin A increase, contributing to the inflammatory environment often referred to as “inflammaging” [1301, 1303, 1304].

One of the major sources of increased circulating Activin A in the elderly is senescent cells, which accumulate with age and secrete pro-inflammatory factors such as activin A as part of the SASP [1305]. Senescent cells contribute to tissue dysfunction and promote chronic inflammation through their SASP factors, including activin A [424, 1306]. These SASP-associated factors drive pathological changes in various tissues, including the vascular system and the brain, exacerbating age-related diseases such as cardiovascular disorders and neurodegeneration.

Several studies have shown a strong association between elevated plasma levels of activin A and an increased risk of age-related cardiovascular diseases [1307]. High circulating levels of activin A have been linked to hypertension, diabetes, carotid intima-media thickness, metabolic syndrome, and frailty [1308–1310]. Additionally, activin A has a dose-dependent effect on endothelial cells. At low concentrations, it promotes endothelial cell proliferation and tubular formation, supporting angiogenesis. However, at higher levels, which are common in older individuals, Activin A impairs endothelial function by increasing oxidative stress, promote endothelial activation and impair angiogenesis [1311–1316]. This dysfunction can lead to vascular inflammation, permeability, and atherosclerosis, accelerating vascular aging and contributing to cerebrovascular diseases such as stroke and vascular dementia [1302].

In the brain, activin A has a complex role [1310, 1317, 1318]. Studies in rodents have demonstrated that activin A can induce the proliferation, differentiation, and survival of neural progenitor cells, suggesting a potential role in promoting neurogenesis and cognitive function. In models of chronic cerebral ischemia, activin A improved neuronal plasticity and cognitive performance [1318]. However, human studies paint a more complicated picture. Higher plasma levels of activin A are associated with cognitive impairment, particularly in older adults. This apparent contradiction may be explained by the antagonistic pleiotropy concept [1302]. While activin A might promote neurogenesis and repair in younger individuals, in older adults, its chronically elevated levels may contribute to neuroinflammation, BBB disruption, and neuronal damage, thereby accelerating brain aging. Studies also indicate that activin A levels in CSF increase with age [1319], particularly in individuals with cognitive impairment.

Given the role of activin A in promoting vascular aging, neuroinflammation, and cognitive decline, targeting this cytokine or modulating its signaling pathways holds potential as a therapeutic strategy to combat age-related diseases. Several experimental approaches, including senolytics to reduce the burden of senescent cells or neutralizing antibodies against activin A, are being explored as ways to mitigate its pro-aging effects [1320–1323]. However, due to its pleiotropic nature, careful consideration is required to balance its beneficial and detrimental effects across different stages of life and disease states.

##### TIMP2

TIMP2 is part of a family of endogenous proteins that inhibit MMPs, enzymes responsible for degrading the extracellular matrix. This function is crucial for maintaining tissue homeostasis, regulating matrix remodeling, and ensuring proper cellular function. However, the balance between MMPs and TIMPs becomes disrupted with age, contributing to various age-related pathologies such as cardiovascular disease and neurodegeneration [1324].

TIMP2 is a circulating factor that declines with age, as demonstrated in multiple studies [1325, 1326]. Castellano and colleagues showed a significant reduction in plasma and hippocampal levels of TIMP2 in aged mice [192]. Interestingly, systemic supplementation with TIMP2, sourced from human umbilical cord plasma, improved cognitive function and synaptic plasticity in older mice [192]. Their research highlights the potential of TIMP2 to reverse age-related neuronal dysfunction, supporting its role as a key factor in the rejuvenation of hippocampal function and cognitive performance [192]. In the context of aging, TIMP2’s neuroprotective effects extend beyond its ability to inhibit MMPs. TIMP2 has been shown to enhance neurogenesis, promote synaptic plasticity, and improve learning and memory in aging models [1327], indicating its potential for therapeutic intervention in age-related cognitive decline, neurodegenerative diseases and VCID [1327]. The homing mechanisms of mesenchymal stem cells, which are crucial for tissue repair and regeneration, are also regulated by TIMP2, further underscoring its role in preserving brain function and neurovascular health [1328].

While TIMP2 shows promise in neuroprotection, its role in cardiovascular health is more complex. TIMP2 is involved in regulating MMP activity in the cardiovascular system, where it modulates processes such as vascular smooth muscle cell migration, plaque stability, and cardiac remodeling following injury. In the heart, TIMP2 deficiency has been shown to inhibit pathological cardiac remodeling after myocardial infarction by reducing the activity of membrane type 1 matrix metalloproteinase, which is involved in the degradation of the extracellular matrix during injury recovery [1329]. However, studies in clinical cohorts suggest that elevated TIMP2 levels may be associated with adverse cardiovascular outcomes. Kelly et al. reported that increased plasma levels of TIMP2, along with TIMP1 and TIMP4, were linked to a higher risk of adverse cardiovascular events following acute myocardial injury [1330]. This dichotomy suggests that TIMP2 may have context-dependent effects, providing beneficial outcomes in certain scenarios while contributing to pathogenesis in others.

MMPs, particularly MMP- 2 and MMP- 9, play a pivotal role in vascular aging and atherosclerotic processes by promoting leukocyte infiltration, VSMC migration, and plaque formation, all of which can lead to plaque rupture and acute cardiovascular events [1331]. TIMP2 acts as an endogenous inhibitor of MMPs, helping to maintain the integrity of the vascular extracellular matrix and reduce the risk of plaque rupture [1331, 1332]. Studies have shown that the balance between MMPs and TIMPs shifts with age, leading to an increased risk of cardiovascular diseases [1333, 1334]. For example, levels of MMP- 2, MMP- 9, and TIMP1 increase in circulation in patients with peripheral arterial disease (PAD) [1335, 1336]. These findings suggest that while TIMP2 may help regulate vascular remodeling and inflammation, its dysregulation can contribute to the progression of atherosclerosis and other vascular pathologies. Moreover, aging is associated with imbalance between circulating TIMP1 and TIMP2, which might exacerbate age-related vascular diseases [1325].

Given its involvement in both neuroprotection and cardiovascular health, targeting TIMP2 may offer therapeutic potential for mitigating age-related diseases. In the context of neurodegeneration, TIMP2 supplementation has shown promise in improving cognitive function and reversing age-related neuronal deficits, making it a potential candidate for interventions aimed at extending cognitive healthspan. In the cardiovascular system, however, the dual role of TIMP2 requires a more nuanced approach. While inhibiting excessive MMP activity through TIMP2 supplementation could help stabilize plaques and reduce vascular inflammation, elevated TIMP2 levels in certain conditions may also contribute to adverse outcomes. Therefore, future therapeutic strategies will need to carefully balance TIMP2’s effects in both the brain and cardiovascular system to maximize its beneficial outcomes while minimizing potential risks.

#### Meteorin-like (Metrnl)

Meteorin-like (also known as meteorin-beta/IL- 41, subfatin or cometin) is a small, secreted cytokine encoded by the *Metrnl* gene, which is highly expressed in adipose tissue, heart, muscle, mucosal tissues, skin and activated macrophages [1337, 1338]. Expression of *Metrnl* is down-regulated in pathophysiological conditions associated with accelerated aging (e.g. in older patients with type 2 diabetes mellitus [1339]) and is up-regulated by exercise [1338, 1340–1343]. Based on findings from isochronic parabiosis experiments joining wild-type and whole-body *Metrnl* knock-out (KO) mice, Metrnl has been proposed to contribute to the regulation of muscle regeneration [965]. Metrnl crosses the blood brain barrier and is abundantly present in the cerebrospinal fluid [1344]. On the basis of its homology with meteorin (with which its shows approximately 40% amino acid identity) it is thought to be able to regulate angiogenesis in the brain and to confer neurotrophic effects [1345–1348]. Important for the present discussion, Metrnl also seems to confer multifaceted cardiovascular protective effects [1337, 1349]. For example, lower serum Metrnl is associated with endothelial dysfunction, and atherosclerosis [1350]. Low serum Metrnl is also associated with higher cardiovascular mortality in patients with heart failure [1349]. The biological effects of Metrnl have not been yet investigated in the context of heterochronic parabiosis.

##### SCF

Stem cell factor (SCF) [1351], also known as c-kit ligand, is a cytokine that plays a critical role in hematopoiesis. It functions primarily by binding to the c-kit receptor (CD117) present on HSCs and progenitor cells, promoting their survival, proliferation, and differentiation [1352]. SCF is essential for maintaining the stem cell niche and supporting tissue regeneration, particularly in the context of bone marrow, but its influence extends beyond hematopoiesis to other tissues and organ systems, including the brain and vasculature. SCF has been implicated in the regulation of mesenchymal stem cells (MSCs), improving their homing and survival, thereby promoting tissue regeneration and repair [1353, 1354]. SCF also plays an important role in vascular biology. It is known to regulate the migration and survival of EPCs, which are essential for maintaining vascular integrity and promoting angiogenesis [1355]. In the CNS, SCF plays a role in neurogenesis and the maintenance of neural stem cells (NSCs). It has been demonstrated that SCF/c-kit signaling is important for the survival and proliferation of NSCs in the hippocampus, a brain region critical for learning and memory [1356–1358].

Stem cell dysfunction is a hallmark of aging. Studies have shown that SCF levels tend to decline with age, which may contribute to the reduced regenerative potential observed in older individuals. The number and function of EPCs decline with age, leading to impaired vascular repair and an increased risk of cardiovascular disease. As SCF levels decline with age, there is a corresponding reduction in neurogenesis, which may contribute to age-related cognitive decline and the development of neurodegenerative diseases. SCF has been shown to counteract some of the deleterious effects of aging by enhancing the proliferation and function of stem cells [1351, 1359]. In preclinical studies, supplementation with SCF has been shown to improve endothelial function, reduce vascular inflammation, and enhance blood flow recovery in models of ischemia [1360]. Research in animal models has shown that increasing SCF levels or activating SCF/c-kit signaling can enhance hippocampal neurogenesis, improve cognitive function, and provide neuroprotection against age-related neurodegeneration [1356, 1358]. SCF’s ability to recruit neural progenitor cells and support their differentiation into mature neurons suggests that it may have therapeutic potential for treating age-related cognitive disorders and promoting brain repair following injury or neurodegeneration. However, there are challenges associated with SCF therapy, including the risk of promoting tumorigenesis, as SCF/c-kit signaling has been implicated in the progression of certain cancers, particularly gastrointestinal stromal tumors and acute myeloid leukemia. Therefore, further research is needed to develop safe and targeted approaches for harnessing the regenerative potential of SCF while minimizing the risk of adverse effects.

### Other systemic factors

#### Soluble Klotho

Soluble Klotho (s-Klotho) is a circulating protein that has garnered significant attention for its role in aging [1361], cardiovascular health [1362], and neuroprotection. It is derived from the cleavage of the membrane-bound Klotho protein, which was first discovered in 1997 and named after one of the three Fates in Greek mythology, responsible for spinning the thread of life [1363]. The membrane-bound form of Klotho serves as a co-receptor for fibroblast growth factors (FGFs), especially FGF23, and is highly expressed in the kidneys and brain [1364]. s-Klotho, the soluble form, is released into circulation and can be detected in plasma, cerebrospinal fluid, and urine, exerting a variety of systemic effects on multiple organs [1364].

One of the key roles of s-Klotho is its protective effect on the vasculature. s-Klotho has been shown to enhance endothelial function, reduce oxidative stress, and inhibit vascular calcification, mechanisms central to cardiovascular diseases like atherosclerosis and hypertension [693, 1365]. In studies involving Klotho-deficient mice, the absence of Klotho leads to accelerated endothelial dysfunction and vascular aging, characterized by increased stiffness of the arterial walls and impaired NO bioavailability, which is critical for maintaining healthy blood vessel function [1366, 1367]. s-Klotho attenuates endothelial dysfunction through several mechanisms. It promotes NO production [692, 1368], limits cellular senescence [1369, 1370], and reduces inflammation [1371] by inhibiting the expression of adhesion molecules like VCAM- 1 and ICAM- 1, which are key mediators of endothelial activation and leukocyte recruitment [1367, 1372]. Parabiosis experiments have provided compelling evidence for the anti-aging effects of s-Klotho [693, 1368]. These studies have shown that the transfer of blood containing higher levels of s-Klotho from young mice to old mice can reverse some of the effects of vascular aging, including restoring endothelial function and reducing vascular stiffness [693, 1366, 1367].

s-Klotho also plays a crucial role in brain health and cognitive function. As individuals age, levels of s-Klotho decline, which has been associated with cognitive deficits and an increased risk of neurodegenerative diseases such as AD [1373]. Animal studies have demonstrated that increasing s-Klotho levels enhances neurogenesis, improves synaptic plasticity, and protects against cognitive decline [1374, 1375]. In particular, s-Klotho has been shown to exert neuroprotective effects by reducing neuroinflammation and oxidative stress, two key contributors to neurodegenerative processes [1376]. It does so by inhibiting the activation of inflammatory pathways and improving the integrity of the BBB [1367, 1375]. Importantly, s-Klotho also interacts with other signaling molecules like FGF23 or platelet factor 4, facilitating its anti-neuroinflammatory and neuroprotective actions [1371, 1377]. In the brain, FGF23 signaling through Klotho has been shown to protect neurons and improve cognitive function, further underscoring the importance of s-Klotho in maintaining brain health as individuals age [1378]. Given its wide range of protective effects, s-Klotho has emerged as a potential therapeutic target for combating both vascular and neurodegenerative diseases associated with aging. Research has shown that higher circulating levels of s-Klotho are associated with better cardiovascular health and a lower risk of diseases such as atherosclerosis, hypertension, and heart failure [1367].

#### Low-Density Lrp1

Low-density lipoprotein receptor-related protein 1 (Lrp1) is a multifunctional receptor widely expressed in various tissues, including the brain, liver, lungs, and vascular endothelium [1379]. Lrp1 plays crucial roles in lipid metabolism, clearance of Aβ, cellular signaling, and maintaining vascular integrity [1380–1383]. As a member of the LDL receptor family, it is involved in mediating the endocytosis of multiple ligands, such as lipoproteins, proteases, protease inhibitors, and extracellular matrix proteins [1384]. In the vasculature, Lrp1 modulates smooth muscle cell function, endothelial cell migration, and response to injury [1382, 1385]. It is also involved in regulating the signaling pathways that control vascular remodeling, including the TGF-β pathway, which influences smooth muscle cell proliferation and differentiation [1386, 1387]. Lrp1 plays a protective role against vascular diseases by modulating the inflammatory response and inhibiting the formation of atherosclerotic plaques [1379, 1383, 1388]. Deficiency in Lrp1 can lead to enhanced susceptibility to vascular injury, development of atherosclerosis, and impaired vessel wall repair [1382, 1385, 1388, 1389].

In the brain, Lrp1 plays a significant role in neuronal health, synaptic function, and protection against neurodegeneration [1381, 1390, 1391]. One of its most important functions is the regulation of brain Aβ levels, through controlling its production and clearance [1379, 1381, 1392]. Lrp1 facilitates the transport of Aβ across the BBB and its subsequent clearance from the brain [1380, 1393, 1394]. Impaired function or reduced expression of Lrp1 in the brain has been associated with decreased Aβ clearance, leading to its accumulation in the brain [1380, 1381]. Moreover, Lrp1 play significant roles in neurotransmission and synaptic plasticity in the adult CNS [1390, 1395, 1396]. It also participates in intracellular signaling pathways that regulate neuronal responses to injury and stress, making it essential for maintaining cognitive function as individuals age [1391].

With advanced age, the expression and functionality of Lrp1 decline in both the brain and the vasculature [1380, 1397]. This reduction is linked to the progression of age-related diseases such as atherosclerosis and neurodegenerative diseases [1397, 1398]. In aging vasculature, Lrp1 deficiency exacerbates endothelial dysfunction, increases vascular inflammation, and promotes plaque formation, contributing to the development of cardiovascular diseases [1382, 1399]. In the aging brain, decreased Lrp1 levels are associated with impaired clearance of Aβ and reduced synaptic plasticity, leading to cognitive decline and an increased risk of neurodegenerative diseases [1397, 1400]. Experimental models have shown that restoring Lrp1 function improves Aβ clearance and mitigates cognitive deficits, suggesting that Lrp1 could be a potential therapeutic target for preventing or treating neurodegenerative diseases in aging populations [1401]. However, other studies demonstrated that global down-regulation of LRP1 led to decreased AB and plaque deposition in the brain [1392], underscoring the complexity of putative treatments aimed at suppressing or enhancing LRP1 activity. While LRP1 is associated with broad anti-inflammatory, anti-atherogenic, and vasculoprotective effects, well-designed and controls studies need to be conducted to establish the potential of therapies aimed at LRP1.

##### VEGF

Vascular endothelial growth factor (VEGF) is a critical signaling protein that regulates angiogenesis, vascular permeability, and the maintenance of vascular homeostasis [1402]. It is a member of a family of growth factors that includes VEGF-A, VEGF-B, VEGF-C, VEGF-D, and placental growth factor (PlGF). VEGF-A is the most studied and is primarily responsible for promoting endothelial cell proliferation, survival, and migration during angiogenesis [1402]. As individuals age, the levels and bioactivity of VEGF decline due to increased levels of soluble VEGFR1 and VEGFR2, which can impair the body’s ability to repair and regenerate blood vessels, contributing to age-related vascular diseases [1403]. A reduction in VEGF signaling is associated with endothelial dysfunction, diminished capillary density, and impaired angiogenesis in older adults [1404–1407]. These changes can exacerbate the development of cardiovascular diseases such as atherosclerosis, hypertension, and ischemic heart disease [1408–1411]. VEGF also has neuroprotective effects in the brain, where it promotes neurogenesis [1412–1414]. Reduced VEGF levels in aging may also contribute to cerebrovascular dysfunction [1408]. In AD, a decrease in VEGF input has been implicated in the progression of both vascular and neurodegenerative pathology [1415]. Additionally, reduced angiogenesis in the aging brain can lead to microvascular rarefaction, further contributing to impaired cerebral blood flow and cognitive dysfunction. Conversely, experimental models have shown that VEGF overexpression or administration can restore some of the damaged vascular and neural tissues in the brain [1416, 1417]. Animal studies demonstrate that increasing VEGF levels can promote neurogenesis, enhance synaptic plasticity, and even improve cognitive functions in models of aging and neurodegeneration [1413, 1418]. Lastly, recent studies have shown that the beneficial effects of young blood-derived factor, GDF11, might originate from increased systemic and brain endothelial cell levels of VEGF and its positive effects on angiogenesis and endothelial function in the brain [203].

#### Apelin

Apelin is an endogenous peptide that functions as the ligand for the G protein-coupled receptor APJ, also known as the apelin receptor (APLNR). The apelin/APJ signaling pathway plays an essential role in maintaining vascular and brain health, particularly during aging [1419, 1420]. In the vascular system, apelin acts as a potent regulator of angiogenesis [1421, 1422], vasodilation, and endothelial function [1423–1426]. Age-related decline in apelin levels is associated with endothelial dysfunction [1427] and loss of vascular integrity [1428]. Apelin has also emerged as an important player in the regulation of brain function, including neuroprotection, cognitive processes, and cerebrovascular integrity, particularly in the context of stroke and ischemic injury [1422, 1429–1431]. Apelin and its receptor are expressed in various regions of the brain, including the hippocampus and cerebral cortex, which are critical for learning, memory, and cognition [1432]. In the context of aging, decline in apelin levels potentially contribute to age-related cognitive decline, neurodegeneration, and cerebrovascular dysfunction [1433]. In animal models of stroke and AD, apelin administration has been associated with improved cognitive outcomes and reduced brain damage [1434, 1435].

#### Thrombospondin- 4 and SPARCL1

Thrombospondins (TSPs) and SPARCL1 (also known as Hevin) are extracellular matrix proteins that play crucial roles in regulating cellular interactions, tissue remodeling, angiogenesis, and synaptic plasticity, making them significant players in both vascular and brain aging [1436–1438]. These proteins are involved in maintaining tissue homeostasis and have been associated with age-related processes, such as inflammation, fibrosis, and neurovascular dysfunction [1439, 1440]. Gan and Südhof employed a systematic screening to explore the effects of specific factors present in the blood of young mice on synapse formation and neuronal function [797]. Their findings indicate that thrombospondin- 4 (THBS4) and SPARCL1, enriched in young serum, directly promote synaptogenesis and increase dendritic arborization in neurons derived from human embryonic stem cells [797]. These factors were found to significantly enhance synaptic connectivity by increasing synapse numbers, promoting dendritic branching, and boosting NMDA receptor-mediated responses. In contrast, serum from old mice did not show these beneficial effects. The study suggests that the rejuvenating effects observed in heterochronic parabiosis are likely driven, in part, by THBS4 and SPARCL1, which directly influence neuronal health and synapse formation [797]. Thrombospondins have also been linked to neurodegeneration. In AD, the accumulation of Aβ plaques may be influenced by the dysregulation of TSP- 1 and TSP- 2, which affect inflammatory and repair processes in the brain [1441–1443]. In neurodegenerative conditions decreased levels of SPARCL1 are correlated with synaptic loss and increased neuroinflammation [1444, 1445].

### SRF pathways

Our recent transcriptomic analysis identified inhibition of SRF-driven pathways as a putative mediator of the effects of old blood on the aorta [241]. It was discovered in 1984 that addition of young serum to quiescent cells in culture rapidly stimulates *c-fos* and thereby promote cell growth [1446]. Subsequent studies showed that several growth factors exert synergistic effects and in the promoter region of serum-responsive genes a short DNA sequence was identified (termed Serum Response Element or SRE) [1447]. SRF is the transcription factor that binds to SRE [1448, 1449] and it was shown to mediate, at least in part, the effects of a range of growth factors present in young sera, including growth hormone [1450], fibroblast growth factor 21 (FGF21) [1451], VEGF [1452], PDGF [1453], and IGF- 1 [1454]. Vascular cells, including vascular smooth muscle cells, are sensitive to the stimulation by young sera, and SRF was demonstrated to regulate several genes expressed in the vasculature [1455–1459] and to regulate angiogenesis [1452]. Genetic inactivation of SRF in mice results in aging-like phenotypic and functional changes in the vasculature, including impaired vasodilation [1456], a pro-fragility microvascular phenotype [1460], and impaired angiogenesis [1460]. It is possible that inhibition of SRF-regulated pathways may also contribute to the effects of old blood in other organs. For example, SRF is down-regulated in aged skeletal muscle in mice and humans and muscle-specific knockdown of SRF was shown to promote muscle atrophy [1461]. Important studies suggest that dilution of young blood factors may contribute to the pro-geronic action of old blood [206, 759]. The role of this dilution effect and the role of specific factors present in old blood, which may interfere with the young blood factors activating SRF-driven pathways should be elucidated in future studies.

### Modulation of NAD^+^ levels

Circulating levels of NAD^+^ and its precursors, such as nicotinamide riboside (NR) and nicotinamide mononucleotide (NMN), decline with age, leading to reduced mitochondrial function, impaired DNA repair, and increased oxidative stress [1462, 1463]. This decline contributes significantly to the aging process across various tissues, including the vascular system [1464, 1465]. SIRT1, a key NAD^+^-dependent deacetylase, plays a central role in regulating endothelial nitric oxide synthase, mitochondrial function in endothelial cells, angiogenesis, and overall vascular homeostasis [1464, 1466–1468]. The reduction in NAD^+^ levels with age limits SIRT1 activity, exacerbating vascular dysfunction and increasing the risk of cardiovascular diseases [1466, 1469, 1470]. Restoration of the NAD^+^/SIRT1 pathway has shown promise in counteracting these age-related effects. Supplementation with NAD^+^ precursors like NMN have been demonstrated to improve endothelial function, restore cerebromicrovascular health, and enhance cognitive performance in aged mice [103, 1471, 1472]. These interventions also reverse age-related transcriptomic changes in cerebrovascular endothelial cells, notably restoring mitochondrial gene expression and reducing oxidative stress [100]. Up to date, several clinical trial tested the efficacy of NAD^+^ boosters in various age-related diseases, including cardio- and cerebrovascular diseases, cognitive disorders, and neurodegenerative diseases [1469, 1473, 1474]. These studies showed that restoring NAD^+^ levels is safe and confers beneficial effects on the vasculature and overall health, including ameliorated arterial stiffness, systolic and diastolic blood pressure, and frailty [1475–1478].

Parabiosis studies have provided initial evidence that young blood can restore the NAD^+^/SIRT1 pathway in aged tissues [179]. Specifically, aged aortas exposed to young blood exhibit transcriptomic profiles that significantly overlap with those activated by NMN and SIRT1, suggesting that factors in young blood may rejuvenate aged tissues by modulating the NAD^+^/SIRT1 pathway [179]. Aging is associated with decline in circulating levels of extracellular nicotinamide phosphoribosyltransferase (eNAMPT), which is carried in extracellular vesicles [622]. Yoshida and colleagues demonstrated that supplementation of extracellular vesicle-contained eNAMPT exerted anti-aging effects, manifested by ameliorated frailty and extended lifespan [622]. These findings demonstrate that age-related decline in NAD^+^/SIRT1 signaling is implicated in cell non-autonomous mechanisms of organismal aging and reinforce the potential of NAD^+^ restoration therapies in mitigating both vascular and cognitive aging.

### Circulating cells

Parabiosis experiments have provided remarkable insights into the potential for blood-based rejuvenation. These studies have consistently demonstrated that young blood can reverse age-related functional, structural and transcriptomic changes in tissues, while old blood accelerates aging processes in young animals, effectively transposing aging phenotypes between the paired organisms. While much attention has been given to the acellular fraction of blood as key drivers of these effects, emerging evidence suggests that the cellular components of blood, including leukocytes, thrombocytes, and endothelial progenitor cells, may also play significant roles in both rejuvenation and degeneration. Understanding how these circulating cells contribute to the expression of aging phenotypes in tissues offers new dimensions for research into age-related decline and potential therapeutic interventions.

#### Circulating leukocytes

Leukocytes are essential to immune surveillance and tissue repair. As organisms age, the population and function of leukocytes shift, contributing to chronic inflammation, known as “inflammaging,” and reduced regenerative capacity. Evidence from parabiosis studies indicates that young blood can enhance immune function and reduce inflammation in aged animals, likely mediated in part by leukocytes [758, 1479–1482]. Bachstetter et al. demonstrated the rejuvenating potential of human umbilical cord blood (hUCB) mononuclear cells on adult neurogenesis [758]. These cells increased the number of proliferating neurons and reduced reactive microglia in the aged hippocampus. Further research identified that CD4^+^ and CD8^+^ T cells derived from hUCB specifically mediated these beneficial effects by reducing inflammation and promoting neurogenesis in the dentate gyrus of aged rats [1479]. Despite these promising findings, the exact mechanisms by which hUCB T cells induce rejuvenation remain elusive. One hypothesis is that age-related increases in BBB permeability allow youthful leukocytes to infiltrate the brain more easily, where they may exert direct or indirect anti-geronic effects on neural tissue through secreted factors. Additionally, in the elderly, clonal hematopoiesis of indeterminate potential (CHIP) has been observed in 10–20% of individuals aged 70 or older [677, 681, 683, 685, 687, 1483, 1484]. CHIP is characterized by somatic mutations in leukocytes and has been linked to chronic inflammation and increased risks of atherosclerosis and cardiovascular diseases [677, 681, 683, 685, 687, 1483, 1484]. This phenomenon may represent another way in which changes in circulating leukocytes contribute to the aging phenotype.

#### Circulating thrombocytes

Thrombocytes are primarily known for their essential role in coagulation and wound healing. Beyond these functions, platelets also contribute to inflammation, immune response, and tissue regeneration by secreting a wide range of bioactive molecules, including growth factors (platelet-derived growth factor, VEGF, IGF- 1, and TGF-β), anti- and pro-coagulation factors, chemokines and cytokines (RANTES), pro- and anti-angiogenesis factors, enzymes, and small molecules [1485]. This secretome allows platelets to influence vascular integrity, tissue repair, and cell survival, making them crucial not only for hemostasis but also for broader physiological processes involved in aging and rejuvenation [1485]. Platelets release a rich mix of signaling molecules upon activation, including growth factors, such as PDGF, BDNF, VEGF, and TGF-β among others. These factors have been shown to have profound effects on vascular and brain health [1413, 1486–1488]. PDGF is a critical regulator of vascular smooth muscle cell proliferation and migration, helping to maintain vascular integrity and promote vessel repair [1489]. PDGF also exhibits neuroprotective effects by supporting the health of neural cells, promoting angiogenesis, and possibly enhancing neurogenesis in specific contexts [1490–1493]. Platelets are also a significant source of VEGF and TGF-β, which are essential for angiogenesis, endothelial cell function, and cerebrovascular health (described in detail above). Furthermore, systemic treatment with young platelets and specifically PF4 have been demonstrated to decrease neuroinflammation and improve hippocampal-dependent cognitive function in aged mice [755, 756, 1377]. On the other hand, with age, platelets undergo several functional changes that contribute to an increased risk of thrombosis, inflammation, and cardiovascular disease. Aging platelets exhibit increased activation and are more likely to aggregate, which elevates the risk of clot formation [1494–1496]. This heightened state of activation is associated with increased secretion of pro-inflammatory molecules, which can exacerbate vascular inflammation and contribute to endothelial dysfunction [1497, 1498]. These age-related alterations in platelet function may not only accelerate the progression of cardiovascular aging but could also have implications for cerebrovascular health and the aging brain, potentially increasing the risk for conditions such as stroke and vascular dementia.

The role of thrombocytes in parabiosis-induced rejuvenation remains largely unexplored, yet given their diverse secretory profile, platelets may contribute to rejuvenative or degenerative processes. Exploring the impact of young versus aged platelet secretome in the context of parabiosis could enhance our understanding of the mechanisms driving vascular and brain health during aging and rejuvenation. In this context it is important that platelet-rich plasma (PRP) therapy has shown promising rejuvenating effects across various tissues, especially in wound healing, dermatology, musculoskeletal repair, and potentially even neuroprotection [1499–1501]. PRP is derived from blood, with concentrated platelets that release growth factors, cytokines, and other bioactive proteins known for their regenerative properties. PRP is widely used in dermatology for its anti-aging benefits [1499, 1502]. Also, PRP therapy has gained popularity in orthopedics and sports medicine for enhancing the repair of tendons, ligaments, muscles, and cartilage [1500]. The growth factors in PRP, including VEGF and IGF- 1, stimulate cellular proliferation and matrix production, promoting tissue repair and potentially delaying degenerative changes. Although PRP has not been as extensively studied for cognitive effects, some preliminary animal studies indicate that PRP may have neuroprotective potential [1501, 1503, 1504]. Further research on the role of platelet-derived factors in tissue maintenance and regeneration could provide insights into potential therapeutic strategies that leverage platelet function to combat age-related vascular and cognitive decline.

#### Circulating endothelial progenitor cells

EPCs play a crucial role in vascular repair and regeneration, maintaining the integrity of the endothelial lining of blood vessels [1505]. With advancing age, the number, health, and function of circulating EPCs decline, resulting in reduced capacity for vascular regeneration and repair [470, 473, 474]. This decline is closely associated with increased cardiovascular risk and poorer recovery outcomes after vascular injury [1506–1509]. EPCs, defined by their expression of VEGFR2^+^/CD34^+^, are mobilized from the bone marrow in response to tissue injury and contribute to both the formation of new blood vessels and the repair of damaged endothelial linings [1510]. Studies have shown that elevated levels of EPCs correlate with better outcomes in tissue repair and recovery following ischemia, including in the brain and cardiovascular system [1511–1516]. For instance, Wang et al. demonstrated that EPC administration protected renal tissue from vascular injury and fibrosis [1517], while Taguchi et al. showed improved recovery from ischemic brain injury in rats following young bone marrow transplantation [222]. These findings underscore the potential role of EPCs in mitigating age-related decline in vascular health. Interestingly, the transposition of aging phenotypes observed in parabiosis suggests that the rejuvenating effects of young blood may also extend to the mobilization and function of EPCs. With young blood exposure, aged animals may benefit from enhanced EPC-mediated vascular repair, whereas young animals exposed to old blood may experience a decline in EPC function, mimicking the vascular aging processes typically seen in older individuals.

### Role of exosomes and extracellular vesicles

Exosomes and extracellular vesicles (EVs) are lipid bilayer-enclosed particles secreted by cells, involved in intercellular communication by transferring proteins, lipids, mRNA, microRNA, and other molecules to neighboring and distant cells [1518]. These vesicles vary in size, origin, and cargo composition, with exosomes (30–150 nm in diameter) being one of the most studied EV subtypes [1519]. Originally thought to be cellular waste, EVs are now recognized as essential mediators of various biological processes, including immune modulation, tissue repair, neuroprotection, and metabolic regulation [1520, 1521]. Their potential for therapeutic applications has gained significant attention, particularly for organismal rejuvenation and amelioration of age-related diseases.

The content and function of exosomes change significantly with age, contributing to the systemic effects of aging [1521]. Aging cells often exhibit increased secretion of EVs enriched in pro-inflammatory cytokines, proteolytic enzymes, detrimental miRNAs, and SASP factors [622, 1521–1524]. These vesicles can spread aging signals throughout the body, promoting cellular senescence and age-related tissue dysfunction [1525]. For example, exosomes from senescent cells can induce DNA damage and oxidative stress in other cells, leading to a transposition of the aging phenotype across tissues [1526]. Importantly, EVs can play both protective and detrimental roles [1527]. Studies have demonstrated that exosomes derived from young cells or tissues possess rejuvenative properties [242, 1528–1532]. It is possible that in parabiosis models, young blood-derived exosomes are responsible for some aspects of organismal rejuvenation [1533, 1534]. These exosomes carry beneficial molecules like growth factors, anti-inflammatory cytokines, and antioxidant enzymes, which can counteract age-associated damage in various tissues such as the brain [1535]. Specific rejuvenative effects include improved mitochondrial function, reduced inflammation, and increased proliferation of stem cells [1535]. In the brain, young-derived exosomes have shown potential in enhancing neurogenesis, ameliorating neuroinflammation, and improving synaptic plasticity and cognitive function, presenting an exciting therapeutic avenue for neurodegenerative conditions [1536–1538]. On the contrary, aging alters the cargo of exosomes, increasing levels of pro-inflammatory and amyloidogenic factors that may contribute to neurodegenerative diseases, such as AD [1539].

Exosomes are also pivotal regulators of vascular health, carrying factors that influence endothelial function and vascular smooth muscle cell phenotypes [1540, 1541]. With aging and in the presence of cardiovascular diseases, endothelial cells secrete more EVs that contain pro-inflammatory and pro-coagulant molecules, contributing to endothelial dysfunction, atherosclerosis, and increased cardiovascular risk [1542, 1543]. We have recently reported that in older patients with MCI, levels of cerebrovascular EVs are increased, which associate with white matter hyperintensities and impaired neurovascular coupling responses [741]. Conversely, treatment with stem cell-derived exosomes enhanced aortic SIRT1 and NOS expression, reduced arterial stiffness, and ameliorated hypertension in aged mice [1544], emphasizing potential role of stem cell- and young blood-derived EVs in amelioration of age-related vascular pathologies. This suggests that targeting or supplementing beneficial EVs may be a promising strategy for treating or preventing age-related cardiovascular pathologies. In summary, exosomes and EVs are emerging as critical players in the regulation of aging and age-related diseases. Their role in intercellular communication and ability to carry pro-aging or rejuvenative signals highlight the importance of continued research into the role of exosomes in parabiosis or plasma transfer-mediated tissue rejuvenation.

### Circulating microRNAs

Heterochronic parabiosis experiments have provided insights into the rejuvenating effects of young blood factors, including circulating exosomes and EVs that carry miRNAs [242, 1545]. These small, non-coding RNA molecules are released by various cells and play significant roles in intercellular communication [1546]. Enclosed within EVs, miRNAs are secreted into the circulation, where they mediate local and systemic effects in organs both close to and distant from their source [1546]. As miRNAs influence gene expression, they are increasingly recognized for their roles in aging and age-related diseases [1547]. Certain miRNAs exhibit distinct expression patterns with aging [1545, 1548]. For example, miR- 126 - 3p, a miRNA involved in vascular health, is elevated in healthy elderly individuals but decreases in patients with metabolic disorders [1549]. The administration of miR- 126 - 5p mimics to atherosclerosis-prone ApoE −/− mice has shown vasculoprotective effects, such as increased endothelial repair, while downregulation of miR- 126 - 5p exacerbated atherogenesis [1550]. Additionally, miR- 126 - 3p may play a role in vascular calcification regulation, positioning it as a potential therapeutic target for cardiovascular diseases [1551, 1552]. Other miRNAs also change with age. MiR- 30c, for instance, rises with age in healthy individuals but decreases in patients with coronary heart disease, with the lowest levels observed in those with concurrent diabetes, indicating a potential role in cardiovascular and metabolic health [1553]. Additionally, c-miR- 21 - 5p increases up to age 80 but then declines in centenarians, suggesting that lower levels may be associated with extended longevity and healthy aging [1548].

In AD, miRNAs in circulation are not only biomarkers of disease but may also contribute to its progression by influencing neuroinflammation, Aβ deposition, and tau pathology [1554]. For instance, miR- 34a has been linked to increased Aβ production, tau phosphorylation, and neuronal apoptosis, and impaired cognitive functions, all of which are hallmark features of AD pathology [1555–1557]. Conversely, miR- 132, typically reduced in AD, is crucial for synaptic plasticity and neuronal health, suggesting that restoring its levels could be neuroprotective [1558]. Studies have shown that miR- 126 - 3p and miR- 146a may impact AD by modulating inflammatory pathways [1559].

Emerging evidence indicates that specific circulating miRNAs could directly impact cellular and molecular aging processes [1548]. Research has shown that hypothalamic-derived exosomal miRNAs influence systemic aging by modulating inflammatory and senescence-related pathways in peripheral tissues [1560]. Further studies are needed to establish whether targeting specific circulating miRNAs can provide therapeutic benefits by modulating their age-related effects on tissues. As our understanding of miRNA functions expands, these molecules hold potential as therapeutic targets, particularly in age-related vascular and CNS diseases.

### Circulating metabolites

Circulating metabolites play significant roles in maintaining systemic and tissue-specific homeostasis. These metabolites are derived from various physiological processes, including nutrient metabolism, hormone signaling, and the activity of enzymes and cellular organelles. Importantly, the composition and concentration of circulating metabolites change significantly with age, which is closely associated with age-related functional decline in the cardiovascular system and the CNS [1561, 1562]. Notably, recent research suggests that metabolites may contribute to the rejuvenating effects observed in parabiosis and plasma exchange experiments, where young blood or plasma appears to confer restorative effects on aged tissues [51, 1563, 1564].

In vascular aging, certain circulating metabolites influence endothelial function, inflammation, and oxidative stress. For instance, amino acids such as tryptophan and its metabolite kynurenine play dual roles in vascular health by modulating inflammation and oxidative pathways [1565]. The kynurenine pathway, for example, has been implicated in vascular aging, with elevated levels of kynurenine associated with endothelial dysfunction and chronic inflammation [1566, 1567]. Similarly, polyamines like spermidine exhibit cardiovascular protective effects, improving endothelial function and reducing vascular stiffness [1568, 1569]. Supplementation with spermidine has been linked to improved autophagy and reduced cellular inflammation in vascular cells, suggesting that polyamine modulation may help counteract age-related vascular decline [1568, 1569].

Changes in circulating metabolites are also closely linked to brain aging, neurodegeneration, and cognitive impairment. Short-chain fatty acids (SCFAs), primarily derived from gut microbial metabolism, are essential for maintaining the integrity of the BBB and reducing neuroinflammation [1570]. Studies have shown that SCFAs like butyrate and propionate can influence microglial function, reducing inflammation and promoting neuronal health [1570]. In AD and other neurodegenerative conditions, reductions in SCFA levels have been correlated with increased neuroinflammation and cognitive decline [1571, 1572]. Another notable metabolite, nicotinamide adenine dinucleotide (NAD^+^), declines with age and has been extensively studied for its impact on cellular energy metabolism, mitochondrial function, and DNA repair. Restoration of NAD^+^ levels in aging mice has shown to improve cognitive function and reduce neuroinflammation, suggesting that NAD^+^ repletion could be a promising strategy for mitigating brain aging (as discussed above) [1573].

Aging is characterized by shifts in circulating levels of metabolites, including acylcarnitines, amino acids, and lipid species. For instance, elevated levels of branched-chain amino acids (BCAAs) and specific lipid metabolites are common in older adults and mice with diabetes. Mice treated with BCAA developed symptoms of AD [1574, 1575]. Simultaneously, antioxidants like glutathione tend to decline with age, resulting in increased oxidative stress and cellular damage [1576]. Lifestyle interventions, including caloric restriction, time restricted feeding and exercise, can modulate circulating metabolites in protective ways. Caloric restriction and ketogenic diets increase ketone bodies like beta-hydroxybutyrate, which may offer neuroprotection and support cognitive resilience against age-related challenges [1577]. In summary, circulating metabolites bridge metabolism, aging, and disease, with profound implications for vascular and brain health. Their potential role in mediating the beneficial effects observed in parabiosis and plasma exchange highlights their value as therapeutic targets for promotion of healthy vascular and cognitive aging.

### Bacterial breakdown products

Bacterial breakdown products, derived from the gut microbiome, play a significant role in modulating processes of aging. With advancing age, the gut microbiome experiences shifts in its composition, leading to increased production of metabolites associated with chronic inflammation, vascular dysfunction, and cognitive decline [1578–1580]. This section examines age-related shifts in microbiome composition, the phenomenon of “leaky gut” and the role of circulating bacterial metabolites in affecting systemic and central nervous system health.

In a healthy adult, the gut microbiome is typically diverse and stable, producing beneficial metabolites like SCFAs, which support immune function and reduce inflammation [1581]. However, with aging, microbiome diversity tends to decline, often accompanied by a reduction in beneficial bacterial species and an increase in pathogenic or opportunistic microbes [1581]. This dysbiosis can shift the balance of microbial metabolites toward a pro-inflammatory profile, leading to the accumulation of harmful bacterial byproducts such as lipopolysaccharides (LPS), secondary bile acids, and trimethylamine N-oxide (TMAO) [1581]. These products can impact systemic health by contributing to chronic inflammation, oxidative stress, and immune dysfunction [1581].

An age-related phenomenon known as “leaky gut” is characterized by increased intestinal permeability, which allows bacterial metabolites to translocate into circulation [1582]. The breakdown of the gut barrier enables bacterial products like LPS to enter the bloodstream, where they activate immune cells and, acting through Toll-like receptors (TLRs), contribute to increased oxidative stress, mitochondrial dysfunction and low-grade, chronic inflammation—often termed “inflammaging” [1583, 1584]. This persistent inflammatory state is linked to numerous age-related conditions, including cardiovascular disease, metabolic syndrome, and neurodegenerative disorders, and underscores the significant impact of microbiome integrity on systemic aging [1585–1587].

Specific bacterial metabolites have direct effects on the cardiovascular system [1588]. For instance, TMAO, produced from dietary choline and carnitine by gut bacteria, has been linked to an increased risk of atherosclerosis and cardiovascular disease [1588]. Elevated TMAO levels are associated with endothelial dysfunction, platelet hyperactivity, and enhanced vascular inflammation, all of which contribute to age-related cardiovascular decline [1589–1592]. SCFAs, conversely, have protective effects on vascular health by promoting anti-inflammatory responses and supporting the endothelial health. Conversely, secondary bile acids and SCFAs have protective effects on vascular health by promoting anti-inflammatory responses and supporting the endothelial health [1593].

Emerging research underscores the influence of the gut microbiome on brain health through the microbiome-gut-brain axis. This axis links microbial metabolites and signaling molecules to brain function and neuroinflammation. SCFAs, such as butyrate, support BBB integrity, reduce neuroinflammation, and promote microglial homeostasis, which are critical for maintaining cognitive function [1581, 1594, 1595]. With aging, reduced SCFA production can compromise the BBB, allowing pro-inflammatory and neurotoxic metabolites to enter the brain [1596, 1597]. Bacterial byproducts have been implicated in the development of neurodegenerative diseases, including AD [1596, 1598, 1599]. Restoring a youthful microbiome profile, or specifically targeting microbial metabolites, may offer promising avenues for therapeutic interventions that support healthy brain aging and longevity.

### Micropeptides

Micropeptides are a recently discovered class of small proteins, typically under 100 amino acids, that were once overlooked in genomic studies due to their small size and the historical assumption that they lacked functional importance [1600, 1601]. Advances in high-throughput sequencing and proteomics have now revealed that micropeptides play vital roles across various biological processes, including regulation of muscle function, metabolism, and cellular signaling. These peptides are encoded by short open reading frames (sORFs) found within previously classified “non-coding” regions of the genome, including lncRNAs and circular RNAs [1600]. Among the best-characterized micropeptides are myoregulin (MLN), dwarf open reading frame (DWORF), sarcolipin (SLN), and humanin, each of which may impact cellular processes of aging in distinct ways [1601]. Although the research on micropeptides in the vascular system and the CNS is still emerging, several key findings link micropeptides to mechanisms that influence vascular integrity, endothelial function, neuroprotection, and cognitive resilience [1602, 1603].

Micropeptides like MLN, SLN, and DWORF play a role in calcium homeostasis, which is crucial for the function of vascular smooth muscle cells and possibly influence age-related changes in VSMC function [1604, 1605]. Sarcolipin’s role in energy expenditure and metabolic balance is relevant to vascular and CNS health as it influences mitochondrial function [1606]. Some micropeptides are associated with anti-inflammatory pathways that may counteract vascular inflammation. Humanin, for instance, has been shown to reduce inflammatory signaling and protect against oxidative stress in endothelial cells [1603, 1607]. Humanin, encoded within the mitochondrial genome, is also noted for its neuroprotective effects [1608, 1609]. By mitigating oxidative stress and reducing apoptosis, humanin contributes to neuronal survival and resilience, protecting neurons from the toxic effects of amyloid-beta [1610]. While specific studies on micropeptides and the BBB are limited, their general effects on endothelial cells and mitochondrial function could extend to cerebromicrovascular health. In parabiosis studies micropeptides may be among the rejuvenating factors or be activated by young blood factors, contributing to observed improvements in endothelial health, and cognitive function. Understanding the interplay between these small peptides and age-related blood factors in parabiosis experiments may reveal new approaches to combating age-related vascular and cognitive decline.

### Circulating autoantibodies

With aging, the immune system undergoes profound remodeling, a phenomenon termed immunosenescence, which includes a heightened tendency toward the generation of autoantibodies. While classical autoimmune diseases are marked by overt autoantibody production that drives pathology and aids diagnosis, accumulating evidence indicates that many older adults also exhibit low-grade, subclinical autoantibody activity. This age-associated autoimmunity, though subtle, is increasingly implicated in the development of “silent” or subclinical pathologies, particularly affecting the vasculature and CNS. Such autoantibodies may exacerbate low-grade inflammation, promoting chronic conditions in the brain and cardiovascular system. Interest in these subclinical autoantibodies has intensified through research on long COVID [1611–1615], where many patients produce autoantibodies that target cardiovascular and endothelial tissues [1614–1616]. These immune responses are believed to underlie a range of long COVID symptoms, including fatigue and cognitive impairment, as well as increased BBB permeability [1614, 1615, 1617]. By elevating vascular inflammation and impacting the CNS, these COVID-associated autoantibodies highlight how low-level autoimmune activity in the elderly might similarly drive age-related vascular and neurological conditions [1618], offering new insights into the role of autoantibodies in aging. Notably, these immune-mediated effects are not exclusive to SARS-CoV- 2 infection—severe infections more broadly appear to accelerate cerebrovascular aging and increase the risk for VCID and AD [1619, 1620]. Thus, the presence of age-related autoantibodies may represent a previously underappreciated mechanism linking systemic inflammation, immune dysfunction, and neurovascular aging. Parabiosis and blood- or plasma-exchange studies have opened new avenues for understanding how immune factors in the blood may influence systemic aging processes. In heterochronic parabiosis, studies have shown that old blood accelerates aging phenotypes in young animals, which could be partly attributed to the transposition of autoantibodies. Plasma exchange, which dilutes or replaces blood plasma, can reduce circulating autoantibodies and thereby alleviate age-related inflammation.

### Role of dilution of circulating pro-geronic factors

It has also been proposed that dilution of old blood plasma per se confers anti-geronic effects in multiple tissues. Importantly, plasma dilution was suggested to attenuate neuroinflammation and improve cognitive function in old mice [759]. It appears that even a single heterochronic blood exchange is sufficient to exert rejuvenating effects in the skeletal muscle of aged mice, decreasing fibrosis and improving tissue regeneration [180]. On the basis of these preclinical findings it was proposed that plasmapheresis could be potentially used as part of rejuvenation therapy to dilute the systemic pro-geronic factors in humans [214]. This is a very interesting idea, which warrants further rigorous testing, both in preclinical and translational settings. The existing data on the effects of TPE (or plasma exchange therapy) in older adults (usually for glomerulonephritis and autoimmune diseases [240]) have not yet revealed anti-geronic effects similar to those observed in experimental models. During TPE the removed plasma is replaced by donor plasma, albumin, or a combination of albumin and saline (usually 70% albumin and 30% saline). An important limitation is that in most cases TPE is performed in patients with significant comorbid conditions that confound the effects of plasma dilution on aging processes. Further, it is likely that rejuvenation by modulating circulating factors requires longer time (e.g., by modulating epigenetic regulatory pathways). TPE can have potentially serious complications, including thrombogenesis, bleeding, transfusion reactions, exposure to transfusion transmitted viral diseases, and suppression of the patient’s immune system (by removal of protecting antibodies).

## Effects of lifestyle on circulating anti-geronic and pro-geronic factors

**Fig. 11:**
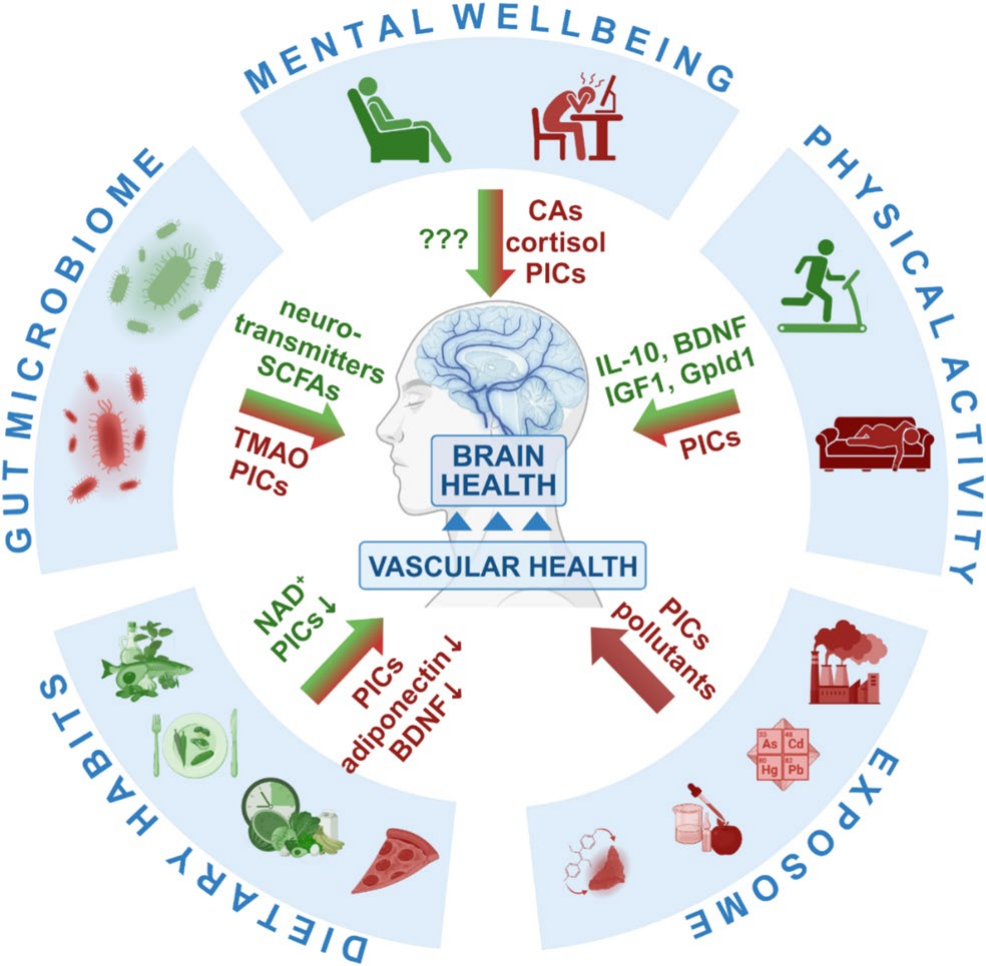
Lifestyle and exposome influence on cerebrovascular and cognitive health via cell non-autonomous mechanisms. This schematic outlines how key environmental and lifestyle factors shape cerebrovascular and cognitive health through systemic, cell non-autonomous mechanisms. Five major categories are depicted, with their respective positive (green) and negative (red) impacts on vascular and brain health. Arrows illustrate the systemic mediators through which these exposures exert their effects, such as circulating anti-inflammatory factors, pro-inflammatory cytokines, growth factors, metabolites, and oxidative stress markers. For example, physical activity enhances levels of IGF- 1, BDNF, and VEGF, promoting cerebrovascular and cognitive resilience. Conversely, chronic stress and environmental toxins elevate SASP factors, inflammatory cytokines, and reactive oxygen species, contributing to vascular dysfunction and cognitive decline. This figure emphasizes the interplay between lifestyle and systemic factors, providing insights into modifiable risk and protective mechanisms for preserving brain health

Organismal aging is influenced not only by intrinsic genetic and molecular factors but also by lifestyle and environmental factors that affect circulating plasma factors involved in regulation of aging processes. Key lifestyle elements such as diet, gut microbiome, exposure to environmental pollutants, chronic stress, sleep quality, and physical activity, significantly impact the circulating systemic milieu, either accelerating or mitigating degenerative changes (Fig. 11). By modulating systemic factors involved in inflammation, oxidative stress, cellular senescence, and mitochondrial function, these lifestyle interventions influence the brain and cerebrovascular systems, and ultimately the aging process.

### Diet

Diet plays a critical role in both promoting health span and pathogenesis of age-related diseases, particularly those impacting cerebrovascular and brain health. Dietary patterns, including food composition, meal frequency and timing, and portion sizes, significantly influence mechanisms of organismal aging, impacting metabolic health, inflammation, and oxidative stress. Recent research demonstrates how dietary habits alter circulating plasma factors, with certain diets promoting a pro-inflammatory, pro-geronic environment, while others foster anti-aging effects (Fig. 11) [1621–1623].

#### The impact of the Western diet on cerebrovascular and brain aging

The Western diet, high in processed foods, refined sugars, unhealthy fats, and food additives, has been associated with accelerated brain aging and increased risk of cerebrovascular diseases, VCID and AD [1624, 1625]. Rich in calories and low in essential nutrients, this diet drives metabolic dysregulation, oxidative stress, and chronic inflammation, promoting neurovascular dysfunction and cognitive decline [108, 1626]. Large epidemiological studies have highlighted strong associations between long-term consumption of saturated fats, obesity, and poor cognitive outcomes [1627–1629]. These diets are linked to increased risk of dementia, reduced cognitive flexibility, and memory impairments.

At the cellular level, high-fat diets (HFDs) in animal models induce substantial cognitive deficits, stemming from widespread neuroinflammation, oxidative stress, and impaired neurogenesis [1630–1632]. Chronic HFD has been shown to exacerbate age-related BBB disruption, endothelial dysfunction, and neurovascular uncoupling, which impairs cerebral blood flow and cognitive functions [108, 1631, 1632]. It also promotes pro-amyloidogenic gene expression and impairs synaptic communication [1631, 1633, 1634]. Plasma from animals on the HFD increases oxidative stress in endothelial cells and promotes activation of microglia, the resident immune cells of the brain, underscoring how poor diet indirectly contributes to cognitive decline via circulating inflammatory mediators [1632, 1635]. In terms of circulating plasma factors, individuals on a Western diet show elevated levels of pro-inflammatory cytokines (TNF-α, IL- 6, IL- 1β, MCP- 1) and atherogenic lipids (LDL, triglycerides) [1632]. Conversely, protective factors like adiponectin and BDNF are significantly reduced, exacerbating the inflammatory milieu in circulation [1636, 1637]. These systemic effects are crucial in mediating Western diet-induced cerebrovascular and brain dysfunction.

The Western diet, which is often rich in red meat and other animal products, is also high in methionine. A high methionine diet has been implicated in accelerating brain and cerebrovascular aging through several molecular mechanisms [1638]. Excess methionine intake can lead to elevated levels of homocysteine, a metabolite associated with increased oxidative stress and inflammation. This condition, known as hyperhomocysteinemia, has been linked to mitochondrial dysfunction, DNA damage, and epigenetic alterations, all of which contribute to cellular aging and impaired cerebrovascular health [1638]. High methionine consumption has been shown to increase oxidative stress within endothelial cells, decreasing NO-mediated vasodilation, which is crucial for proper blood flow and vascular health. It also promotes cerebromicrovascular rarefaction and vascular inflammation. Over time, this oxidative stress and inflammation can lead to endothelial dysfunction, reduced cerebral blood flow, and neurovascular uncoupling. Additionally, methionine-related metabolites may contribute to BBB disruption, facilitating neuroinflammation and potentially increasing the risk of neurodegenerative diseases.

#### Anti-aging dietary interventions and their protective effects

In contrast to the Western diet, anti-aging dietary interventions like caloric restriction (CR), intermittent fasting (IF), and adherence to the Mediterranean diet are linked to improved healthspan, decreased risk of cerebrovascular diseases, and enhanced cognitive resilience [1639, 1640]. As one of the most studied anti-aging interventions, CR reduces caloric intake by 20–40% without malnutrition, conferring protection against cerebrovascular and neurodegenerative diseases [1640, 1641]. Preclinical studies demonstrate that CR extends lifespan across various rodent species, accompanied by broad vasculoprotective and neuroprotective effects [1642]. CR enhances endothelial function by increasing NO bioavailability, reducing vascular oxidative stress, and enhancing mitochondrial health [1642]. By promoting angiogenesis, reducing neuroinflammation, improving neurovascular coupling, and enhancing BBB and CBF, CR helps maintain cerebrovascular and cognitive health [325, 1640, 1643, 1644]. Mechanistically, CR activates nutrient-sensing pathways, such as AMPK, mTOR, and SIRT1, which regulate cellular metabolism, autophagy, and stress resilience [1645]. Notably, CR induces favorable shifts in circulating plasma factors, including increased levels of NAD^+^ and decreased levels of pro-inflammatory cytokines, which contribute to its cerebrovascular protective effects [229, 1646].

While CR is a powerful intervention, its restrictive nature presents adherence challenges. IF, which restricts eating to specific time windows, is a more feasible alternative shown to offer similar benefits [1647]. IF has been associated with reduced systemic inflammation and lower levels of CRP, TNFa, and IL- 6, all contributors to vascular aging and cognitive decline [1648, 1649]. In preclinical studies time restricted feeding (TRF) was shown to confer endothelial protective effects, which associate with improved cognitive performance in aged mice [1650]. Moreover, IF increases circulating ketone bodies, which provide an alternative energy source for neurons and offer neuroprotective effects by reducing oxidative stress and enhancing mitochondrial function [1651]. Produced via oxidation of free fatty acids during fasting or low-carbohydrate intake, ketone bodies (e.g., beta-hydroxybutyrate (BHB)) serve as alternative energy sources, especially in the aging brain [1652]. Not only do ketones support cognitive function, but they also act as signaling molecules with neuroprotective and vasculoprotective effects [1653]. Research highlights that BHB promotes mitochondrial biogenesis, reduces oxidative stress, and upregulates anti-inflammatory pathways [1653], resulting in improved neurovascular coupling, BBB stability, and cognitive function ([1654]; Tarantini S. et al., 2024, unpublished observations). Since aging decreases metabolic flexibility and ketone levels, IF or pharmacological interventions that increase ketone production may represent a promising anti-aging strategy to counteract age-related cerebrovascular decline.

Rich in fruits, vegetables, whole grains, olive oil, and fish, the Mediterranean diet is linked to longevity and reduced risk of cerebrovascular diseases, VCID and AD [1655–1657]. This diet reduces circulating pro-inflammatory markers, such as TNF-α and CRP, while boosting anti-inflammatory and antioxidant compounds like omega- 3 fatty acids and polyphenols, which activate anti-aging cellular signaling pathways (e.g., SIRT1 signaling) [1658–1661]. In terms of cerebrovascular health, the Mediterranean diet enhances endothelial function by stimulating nitric oxide production, reducing arterial stiffness, and improving cerebral blood flow, thereby decreasing the risk of stroke and VCID [1658, 1662, 1663]. Clinical studies show associations between the Mediterranean diet and improved cognitive performance [1664–1666], suggesting its benefits may stem from systemic effects on inflammation, lipid metabolism, and oxidative stress.

#### Plasma transfer studies in dietary interventions

Parabiosis and plasma exchange studies provide additional insights into the systemic effects of dietary factors on aging. Transferring plasma from animals on calorie-restricted diets or animals following a healthy diet to those on an HFD has demonstrated the potential of diet-modulated circulating factors to counteract negative effects or mediate aging processes on the cerebrovasculature [1632]. Plasma from calorie-restricted animals improves vascular function, reduces oxidative stress, and enhances neurogenesis in recipient animals, indicating that plasma factors from a healthy dietary background carry anti-geronic potential [1635, 1667]. Similarly, studies using plasma transfer from young or caloric-restricted animals to older recipients highlight the systemic influence of diet on circulating factors, supporting the idea that certain dietary habits can shape the systemic environment to protect against vascular and neurodegenerative decline. Future research is needed to further elucidate the exact circulating factors involved and to explore therapeutic applications for humans.

Taken together, diet is a major modulator of circulating plasma factors that influence systemic aging processes, impacting both cerebrovascular and brain health. While the Western diet accelerates age-related pathologies through pro-inflammatory and oxidative stress-inducing effects, dietary patterns such as caloric restriction, intermittent fasting, and the Mediterranean diet show potential to protect against these age-related declines. Their effects, mediated through specific circulating factors, offer insights into potential dietary interventions to slow vascular and neurodegenerative aging, highlighting the importance of diet in shaping the systemic environment in support of brain and vascular health.

### Obesity and accelerated aging

Obesity is increasingly recognized as a significant factor in accelerated aging, with effects that extend beyond metabolic health to impact vascular integrity, brain function, and lifespan [110, 257, 1668], contributing to chronic conditions associated with deregulated nutrient sensing, telomere attrition, epigenetic alterations, mitochondrial dysfunction, stem cell exhaustion, and altered intracellular communication [1669]. Characterized by excessive accumulation of adipose tissue, obesity influences systemic aging processes, particularly through an altered profile of circulating factors [1670]. This profile includes adipokines, inflammatory cytokines, and metabolic hormones, all of which collectively contribute to cerebrovascular and brain aging [1671, 1672]. As a pro-inflammatory and pro-oxidative state, obesity exacerbates age-related dysfunctions in the vascular system and the CNS, driving the pathogenesis of VCID and AD [1673, 1674].

In obesity, adipose tissue functions as a dynamic endocrine organ, releasing numerous bioactive molecules known as adipokines [1672]. These molecules include leptin, adiponectin, resistin, and range of pro-inflammatory cytokines such as IL- 6 and TNF-α [1672, 1675]. Adipose tissue also secretes free fatty acids, which circulate in excess during obesity and contribute to a pro-inflammatory and pro-atherogenic state [1676]. This altered circulating milieu is central to the impact of obesity on cellular and molecular mechanisms of aging, linking obesity to accelerated brain and cerebrovascular aging [1632, 1677]. In particular, the chronic low-grade inflammation and oxidative stress associated with obesity accelerates the development of atherosclerosis, endothelial and neurovascular dysfunction, microvascular rarefaction, BBB disruption and neuroinflammation [1632, 1678–1683]. These obesity-induced cerebrovascular and brain pathologies have been linked to impairments in memory, executive function, and spatial cognition, and acceleration of age-related cognitive impairment [257, 1682, 1684].

### Gut microbiome

The gut microbiome, a complex community of thousands of microbial species in the gastrointestinal tract, goes beyond its core roles in digestion and metabolism to influence immune regulation, hormone modulation, and critical interactions with the brain. Through the gut-brain axis, the microbiome communicates with the brain by releasing bioactive compounds, such as neurotransmitters, cytokines, bacterial metabolites, and SCFAs [1685, 1686]. Notably, anti-inflammatory SCFAs, including butyrate, acetate, and propionate, help maintain BBB integrity and support cerebral blood flow regulation [1687]. Furthermore, gut-derived neurotransmitters like serotonin, dopamine, and GABA influence mood, behavior, and cognition, while the microbiome also affects the hypothalamic–pituitary–adrenal (HPA) axis, modulating cortisol levels and the body’s stress response [1688].

Lifestyle factors, including diet, exercise, chronic stress, and sleep, significantly impact microbiome composition [1689]. A diet high in processed foods and refined sugars but low in fiber, for example, leads to dysbiosis, characterized by reduced microbial diversity and an overgrowth of pro-inflammatory bacteria. This imbalance results in the release of harmful pro-inflammatory factors (e.g., TNF-α, IL- 6, IL- 1β) and bacterial products like lipopolysaccharides, contributing to systemic inflammation and oxidative stress [1690]. Furthermore, long-term adherence to a Western diet, high in fats and sugars, worsens these effects by fostering the production of TMAO and other vasculotoxic metabolites, linking dysbiosis with vascular damage and cognitive impairment [1691, 1692]. Shifts in microbiome composition associated with unhealthy aging, including reduced microbial diversity and a decline in beneficial butyrate-producing bacteria, further exacerbate systemic inflammation and neuroinflammation [1693, 1694].

Experimental models have demonstrated that age-related dysbiosis can be partially reversed. For example, co-housing aged mice with young ones or transplanting young microbiota improves hippocampal neurogenesis, enhances BBB function, and reduces neuroinflammation, likely by restoring butyrate-producing bacteria [1695, 1696], while fecal microbiota transplantation from aged or AD mice increased neuroinflammation, worsened brain resilience, and impaired learning and memory [1697, 1698]. Additionally, interventions like heterochronic parabiosis and heterochronic plasma transfer suggest that a young systemic milieu can rejuvenate the aged microbiome, reducing LPS levels and restoring beneficial microbial species [1699]. The close relationship between the microbiome, systemic milieu, and brain health highlights potential therapeutic targets. Modulating the gut microbiome through lifestyle changes, dietary interventions, and possibly microbiome-based therapies may provide avenues for preserving brain health and mitigating age-related cerebrovascular and cognitive decline.

### The exposome: environmental factors in vascular and brain aging

Environmental exposures collectively shape the “exposome,” impacting the body’s systemic environment by triggering inflammation, oxidative stress, mitochondrial damage, and metabolic dysfunction, thus accelerating the pathogenesis of age-related chronic diseases [1700]. Key components of the exposome, such as air pollutants, dietary additives, pesticide residues, heavy metals, and endocrine-disrupting chemicals, contribute to chronic low-grade inflammation and oxidative stress, affecting vascular, metabolic, and brain health (Fig. 11) [1701]. These exposures may act synergistically with age-related pro-geronic factors to exacerbate cerebrovascular and cognitive decline, emphasizing the importance of integrative, exposome-based models of brain aging.

As metropolitan populations grow, driven by urbanization, industrialization, and car-centered city planning, air pollution has become a serious public health concern [1702]. Air pollution contains a mix of hazardous components, including fine particulate matter (PM2.5), nitrogen dioxide (NO_2_), ozone (O_3_), and volatile organic compounds (VOCs) [1703]. The health consequences of air pollution are profound and extend beyond the respiratory system [1703]. Once inhaled, pollutants like PM2.5 and NO_2_ enter the bloodstream, inducing systemic inflammation through the release of pro-inflammatory cytokines [1704]. These molecules amplify systemic inflammation and exacerbate oxidative stress [1704], which promote endothelial dysfunction, atherogenesis, BBB disruption, neurodegeneration [1705–1707].

The increasing reliance on processed foods in modern diets has resulted in greater exposure to a variety of dietary toxicants, including preservatives, artificial sweeteners, pesticide residues, and food additives. These substances, used to prolong shelf-life and enhance taste, might accelerate pathogenesis of chronic diseases in long-term exposure [1708]. Preservatives (e.g., nitrates, nitrites, and sulfites), artificial sweeteners (aspartame and its metabolites, sucralose, saccharin), residues of pesticides (organophosphates), emulsifiers (carboxymethylcellulose and polysorbate 80), and certain food colorants (tartrazine) have been shown to promote gut dysbiosis, mitochondrial dysfunction, oxidative damage, and inflammation, leading to neurotoxicity and neurodegeneration [1709–1712]. Although the effects of individual food additives or pesticide residues might seem negligible, their interactions with other toxicants, long-term exposure, and synergy with components of the Western diet (such as refined sugars and trans and saturated fats) may amplify their harmful effects, worsening cerebrovascular health and increasing the risk of neurodegenerative diseases [1713].

Another significant environmental threat to public health is exposure to heavy metals such as lead, mercury, cadmium, and arsenic, particularly in urban and industrial regions where air, soil, and water contamination are common. These heavy metals come from various sources, including industrial emissions, outdated plumbing systems, tobacco smoke, and contaminated seafood and water [1714]. Once absorbed, these metals increase circulating levels of free radicals and pro-inflammatory cytokines such as TNF-α, interleukin- 1β, interleukin- 6, interleukin- 8, and interleukin- 12, promoting oxidative stress, systemic inflammation, and organ toxicity [1714]. Chronically, these metals accumulate in body tissues leading to endothelial damage and dysfunction, vascular inflammation, BBB breakdown and neuroinflammation, neurodegeneration, and cognitive impairment [1714–1716].

In modern life, endocrine-disrupting chemicals (EDCs) have become nearly impossible to avoid, as they are found in a wide range of everyday products. These chemicals, present in plastics and food packaging (e.g., bisphenol A), cosmetics (e.g., phthalates), and pesticides (e.g., DDT), interfere with the body’s endocrine system [1717]. EDCs mimic or disrupt natural hormones like estrogen, testosterone, thyroid hormones, and cortisol, leading to hormonal imbalances that contribute to chronic inflammation, oxidative stress, and metabolic dysfunction [1718]. The impact of EDCs is reflected in alterations to the systemic milieu, as chronic exposure elevates levels of pro-inflammatory cytokines such as TNF-α, CRP, and IL- 6, fueling systemic inflammation [1719].

It becomes evident that the toxic effects of environmental pollutants are often mediated by circulating endogenous and exogenous factors, including pro-inflammatory cytokines (TNFα, IL- 6, CRP), oxidative stress markers (MDA, 4-HNE), lipopolysaccharides, TMAO and other microbial metabolites, as well as endogenous hormones (estrogen, testosterone) and the pollutants themselves (e.g., PM2.5, heavy metals, xenobiotics). Mechanistic in vitro studies have shown that endothelial cells treated with sera or extracellular vesicles from mice or humans exposed to environmental factors exhibit increased oxidative stress, inflammation, permeability, and apoptosis [1720–1722]. As exposure to environmental pollution becomes increasingly unavoidable, identifying and targeting these circulating factors and their biological consequences may offer new strategies to mitigate the harmful effects of environmental exposure and chronic diseases that such exposures would otherwise induce.

### Stress (hypothalamic-pituitary axis)

Chronic stress accelerates aging and contributes to numerous chronic diseases through its impact on the systemic milieu [1723–1725]. The body’s stress response, primarily regulated by the hypothalamic–pituitary–adrenal (HPA) axis and the sympatho-adreno-medullary (SAM) axis, involves the release of cortisol and catecholamines, which are essential for short-term adaptation [1726]. However, chronic stress dysregulates this system, leading to sustained inflammation, oxidative stress, and hormonal imbalances [1727]. A significant consequence of prolonged stress is metabolic dysfunction, marked by elevated LDL cholesterol, triglycerides, insulin resistance, and reduced protective adipokines like adiponectin (Fig. 11) [1728–1730].

Furthermore, chronic stress is also associated with increased cardiovascular disease risk [1731]. The vasculature is particularly susceptible to stress-related damage [1732]. Elevated cortisol and catecholamines promote systemic inflammation by raising pro-inflammatory cytokines and circulating immune cells, which exacerbate vascular aging mechanisms [1731]. These factors promote vascular inflammation, which is further exacerbated by elevated levels of circulating catecholamines (e.g., epinephrine and norepinephrine). Additionally, chronic stress-induced mitochondrial dysfunction and inflammation lead to increased oxidative stress, damaging the vasculature and reducing nitric oxide bioavailability, thereby impairing endothelial function and compromising blood flow to various organs [1732, 1733]. Chronic stress also causes damage to the brain’s microvasculature, leading to the formation of white matter lesions, cerebral microbleeds, and microvascular rarefaction [1734–1737]. These structural changes, coupled with endothelial dysfunction, result in brain hypoxia and impaired cerebral perfusion, accelerating cognitive decline [1733, 1737].

Besides the cerebrovasculature, other components of the neurovascular unit, particularly neurons, are also severely affected by chronic stress. Elevated and sustained levels of cortisol lead to neuroinflammation and neurotoxicity, causing the loss of both neurons and synapses, observed in prefrontal cortex and hippocampus, brain regions critical for decision making, learning, and memory [1738, 1739]. Moreover, chronic stress accelerates brain aging, not only through structural damage but also by promoting neuroinflammation, oxidative stress, and telomere shortening. These processes contribute to cognitive decline and increase the risk of neurodegenerative diseases such as Alzheimer’s disease [1739]. Stress-induced BBB dysfunction, increased amyloid-beta accumulation, and the disruption of normal tau protein metabolism also create an environment conducive to Alzheimer’s disease pathology, further compounding the effects of aging on the brain [1739, 1740].

### Physical activity

Physical activity is fundamental for maintaining health, yet with modern advancements, daily life has become increasingly sedentary, contributing to metabolic dysfunction, obesity, and accelerated aging [1741–1743]. Lack of exercise contributes to systemic inflammation, oxidative stress, and impaired cerebral blood flow, elevating risks for cerebrovascular and neurodegenerative diseases [1744–1747]. These effects stem from endothelial dysfunction, mitochondrial dysfunction, and inflammatory cytokine elevation, which collectively compromise vascular and brain health [1746]. Fortunately, the negative consequences of a sedentary lifestyle can be mitigated through regular physical activity. A growing body of clinical evidence highlights the positive impact of exercise on both cerebrovascular and cognitive health in healthy individuals, as well as those suffering from metabolic disorders, obesity, MCI, and AD [1748, 1749]. Exercise has been demonstrated to ameliorate BBB disruption and neuroinflammation, enhance cerebral vascularization and endothelial function, all of which contribute to better cognitive health and resilience [1749–1751].

The beneficial effects of exercise are largely mediated by changes in circulating plasma factors. Animal studies have shown that transferring plasma from physically active mice to sedentary mice significantly reduces neuroinflammation, promotes neurogenesis, and improves cognitive function [1752]. These improvements are driven by exercise-induced circulating factors known as “exerkines,” which include a variety of neurotrophic factors (e.g., gpld1, clusterin, BDNF, IGF- 1), anti-inflammatory cytokines (e.g., IL- 10, IL- 1RA, IL- 6), myokines (e.g., irisin, cathepsin B, FGF21), adipokines (e.g., adiponectin), and short-lived mediators like ATP and NO [1073, 1752–1756]. Moreover, physical activity ameliorates the dysfunction of HPA axis, reducing cortisol levels and protecting against stress-induced vascular and CNS damage [1757, 1758]. Overall, the multifaceted systemic effects of physical activity underscore its role as a powerful modulator of brain and cerebrovascular health, counteracting the detrimental effects of aging and offering a promising approach to prevent cognitive decline. Further research on exerkines and their potential in therapeutic applications is essential for translating these benefits to clinical settings.

## Translational considerations

The rapidly expanding field of rejuvenation and aging research has uncovered multiple promising strategies for potentially translating findings from preclinical studies to human therapies. To bring these therapeutic approaches into clinical use, several avenues merit exploration. One traditional approach involves the pharmaceutical development of isolated molecules, such as NAD^+^, GDF11, or specific exerkines, that have demonstrated robust anti-aging properties. This would follow the classical pathway of drug development, encompassing preclinical studies, phased clinical trials, and regulatory approvals. Alternatively, leveraging an organism’s intrinsic capacity to produce these rejuvenative molecules may hold promise. However, the complexity and risk associated with non-synthetic bioactive molecules, particularly of non-human origin, present a barrier to intravenous administration in humans. Consequently, human blood products or their derivatives offer a feasible route for safely testing these therapies.

### Clinical investigations

The use of blood and plasma products in clinical medicine, particularly in autoimmune and inflammatory diseases, has sparked interest in their potential applications for age-related conditions, including neurodegeneration. Plasma exchange, for instance, has been a valuable approach for removing pathogenic antibodies and inflammatory mediators in conditions such as autoimmune disorders and sepsis. Inspired by the rejuvenative effects observed in heterochronic parabiosis studies, current research explores how similar modulation of the systemic milieu might mitigate brain aging and cognitive decline [1759].

A promising direction in clinical trials includes evaluating the effects of young plasma and umbilical cord blood plasma, which are rich in youthful systemic factors that could positively influence brain health. In this context, Alkahest’s clinical trials involving young plasma for late-stage AD and Parkinson’s disease stand out as pioneering efforts [1760]. In their initial studies, the authors demonstrated that treatment with specific fractions of young plasma (e.g., GRF6021, GRF6019) are safe and well tolerated in AD and PD patients (Table 2). Additionally, other researchers have shown that repeated intravenous injection of young plasma are safe for patients with neurodegenerative diseases and can improve their quality of life [195, 1761]. Importantly, small clinical trials aimed at determining the safety of human umbilical cord blood (hUCB)-based treatments shown that these treatments are well-tolerated and associated with minor adverse effects [374, 1762]. It has been demonstrated that repeated intramuscular injection of hUCB ameliorated biomarkers of aging and biological age [374], Table 2. Moreover, there are several undergoing clinical trials testing the efficacy of young blood-based therapies in the context of organismal aging (e.g., young platelet-rich plasma, NCT03647917).

**Table 2:** Clinical investigations on blood-based therapies in neurodegenerative diseases and aging

Type of therapy	Intervention and research strategy	Targeted disease and key findings	Safety and tolerability	Study IDs and references
Young blood plasma	Young fresh frozen plasma One unit of yFFP weekly for four weeks, With or without within-control group, *N* = 18	AD: safety and tolerability study. Sample size too small to draw conclusions on effectiveness.	Safe and well-tolerated, AEs include hyper-tension, dizziness, and headache.	NCT 02256306 Sha et al., 2019
	Young plasma fraction GRF6019 Daily intravenous injection of GRF6019 for five days or placebo, *N* = 18/8	AD: Safety and Tolerability Study. Sample size too small to draw conclusions on effectiveness.	Safe and well-tolerated, rate of AEs comparable between groups.	NCT 03765762 Hannestad et al., 2021
	Young FFP One unit of yFFP twice a week for 4 weeks, *N* = 15.	PD: Exploratory outcomes: improved phonemic fluency and quality of life (PDQ- 39)	Safe, no severe AEs were reported	NCT 02968433, Parker et al., 2020
	Young plasma fraction GRF6021 Two treatments consisting of five daily *i.v. *GRF6021 injections or placebo; N= 53/26	PD: Mixed results regarding cognitive outcomes and no improvements in quality of life. Fine motor skills and reaction time improved	Safe, no severe AEs were reported	NCT03713957
Human umbilical cord blood transfer	Human umbilical cord blood transfer Ten intramuscular injections of hUMB concentrate, one a week, *N* = 18	Aging: Decreased biological age (DNA methylation-based epigenetic clock) and ameliorated more than 20 biomarkers of aging.	Safe	Clement at al., 2022
	hUCB-derived mesenchymal stem cells Three intracerebroventricular injections, once a month, low/high dose, *N* = 3/6)	AD: Safety and tolerability were the primary outcomes measured in this study.	Transient symptoms such as fever, headache, and nausea observed in majority of patients.	NCT 03172117
Therapeutic plasma exchange	TPE with albumin +/− IVIG (4 groups: low-albumin, low-albumin+IVIG, high-albumin+IVIG, and control, *N* = 82/92/68/87).	AD: Improved cognitive outcomes, ameliorated cognitive regression	Safe and well tolerated. Number of AEs comparable between groups	NCT 1561053 Boada et al., 2020
	TPE with 5% albumin (six TPEs over 3–6 weeks, *N* = 21, or sham TPE, *N* = 21)	AD: Decreased plasma Aβ_1–40_ and Aβ_1–42_, improved cognitive functions	AEs such as device-related infections, anemia, and anxiety	NCT 00742417 Boada et al., 2017
Isolated molecules	Enhancing the NAD+ pathway (2 groups: Placebo control, Nicotinamide riboside (NR) treatment (N= 10/10)	MCI: Increases NAD+ levels, alters CBF in DMN regions linked to MCI and AD	A 10-week, 1 g/day oral NR admin-istration is safe in older adults with MCI	NCT02942888 Orr et al., 2023
	Estrogen-based hormone therapy (4 groups: 17β-estradiol (17βE), conjugated equine estrogen (CEE), discontinued 17βE therapy, discontinued CEE therapy, *N* = 16/12/13/4)	Dementia: Women who discontinued HT showed decline in the medial frontal cortex; both discontinuing 17βE-based HT and continuing CEE-based HT led to posterior cingulate/precuneus decline, with no change for those continuing 17βE-based HT or discontinuing CEE	Safe but not specify the tolerability	NCT 00097058 Rasgon et al., 2014
Intravenous immunoglobulin treatment	*I.v.* Immunoglobulin Bimonthly IVIG infusions for 18 months (0.2 g/kg, *N* = 98, 0.4 g/kg, *N* = 94) or low-dose albumin (placebo group, *N* = 90)	AD: No significant differences on the 11-item cognitive subscale of the AD Assessment Scale	Safe and well-tolerated in moderate AD patients	NCT 00818662, Relkin et al., 2017
	*I.v.* Immunoglobulin Five infusions of 10% IVIG (0.4 g/kg) or 0.9% saline, every 2 weeks (*N* = 26/26)	AD: Cognitive improvements and ameliorated brain atrophy observed 1 year after treatment subsequently decreased over 5 years.	Good tolerability with slightly increased incidence of mild AEs	NCT01300728, Kile et al., 2018 Kile et al., 2021

TPE is a well-established treatment in traditional medicine, commonly used for managing antibody-mediated disorders and familial hypercholesterolemia. In aging research, plasma dilution, a process of removing and replacing portions of plasma while retaining cellular elements, offers a theoretical approach to reduce pro-aging factors without completely altering the systemic environment. Preclinical studies have shown that TPE can ameliorate amyloidopathy, neuroinflammation, and cognitive impairment in animal models of AD and in healthy aged mice [759, 1763, 1764]. Importantly, recent studies have assessed the safety, tolerability, and efficacy of TPE with albumin replacement, as well as intravenous immunoglobulin (IVIG) injections, in AD patients [856, 859, 1765–1767]. These studies demonstrated that TPE, TPE with albumin replacement, and IVIG are safe, well-tolerated, and produce a similar rate of mild adverse effects compared to control groups. Research by Mercè Boada has shown that TPE with albumin replacement can improve cognitive symptoms and slow cognitive decline in AD patients [856, 859], while IVIG-based treatments yielded mild-to-moderate cognitive improvements up to two years post-treatment [1765–1767]. Importantly, several clinical trials aimed to assess the safety and efficacy of plasmapheresis in age-related cognitive and neurodegenerative diseases are currently enrolling patients (e.g., NCT06234436). As the field of blood-based therapeutics advances, the development of plasma and blood exchange therapies for brain aging and neurodegeneration will require further rigorous clinical investigations. Establishing the long-term safety and efficacy of these therapies is crucial to transitioning them from experimental models to viable treatments.

### Risks and side effects of blood transfusions and therapy with plasma and plasma fractions

Although transfusions and plasma therapies are essential tools in medicine with broad clinical applications, they carry inherent risks that must be carefully managed, particularly as they are explored for potential anti-aging applications. Transfusions are routine in patient care, particularly to replenish cellular components such as red blood cells or platelets and to address coagulation deficiencies through fresh frozen plasma (FFP). More recently, interest has expanded to therapeutic applications in regenerative and anti-aging contexts, primarily in research settings. However, translating plasma or blood therapies from clinical necessity to exploratory treatments in aging requires a comprehensive understanding of both known risks and long-term safety considerations [1768].

#### Non-immune-mediated complications

Non-immune-mediated complications include transfusion-associated circulatory overload (TACO), transfusion-transmitted infections, and metabolic imbalances. TACO, caused by volume overload from transfusion, poses a significant risk to older adults and those with cardiovascular conditions. Additionally, although strict screening has reduced transmission risks, transfusions still carry the potential for infection, especially in immunocompromised individuals. Further, metabolic shifts, such as electrolyte disturbances, may arise due to stored blood product transfusion, particularly in large volumes [1769].

#### Immune-mediated complications

Immune-mediated reactions include acute and delayed hemolytic transfusion reactions (AHTR and DHTR), transfusion-related acute lung injury (TRALI), and transfusion-associated graft-versus-host disease (TA-GVHD) [1769]. AHTR and DHTR occur when recipient antibodies attack transfused RBCs, leading to symptoms ranging from mild fever to severe multi-organ failure. Pre-transfusion compatibility testing is essential in mitigating these reactions. TRALI, often due to donor antibodies targeting recipient leukocytes, can result in acute lung injury in predisposed individuals. TA-GVHD occurs when transfused donor lymphocytes attack recipient tissues. The original preventive strategy against TA-GVHD has been the irradiation of blood products, more recently, leukoreduction and pathogen inactivation have greatly contributed to the profound decrease in risks. A notable long-term effect of repeated exposure to foreign cells through transfusion is the development of antibodies, particularly against human leukocyte antigens (HLA). This immunization process complicates future compatibility for organ transplantation, as sensitized patients may reject a broader range of potential donor organs due to the presence of these antibodies [1769].

In cases where there is an unintended mismatch, donor anti-RBC allo-antibodies may interact with the recipient’s red blood cells. Such reactions, however, are typically mild and short-lived, as donor antibodies are quickly used up. Similarly, donor-derived anti-platelet antibodies can occasionally target recipient platelets, causing transient platelet destruction (platelet-lysis) that usually resolves without noticeable symptoms. In some instances, recipient antibodies and donor cytokines may contribute to febrile non-hemolytic transfusion reactions, which are generally mild and respond well to antipyretic treatment. In rare cases, recipient antibodies may target soluble elements in the transfused product, causing allergic transfusion reactions. Another delayed and uncommon complication is post-transfusion purpura, where recipient antibodies against donor platelet antigens lead to a temporary drop in platelet counts [1769].

Transfusion-related immunomodulation (TRIM) describes the potential of blood products to alter immune responses, which may have therapeutic or adverse effects [1770, 1771]. In autoimmune or inflammatory conditions, TRIM may provide immune benefits; however, it could also exacerbate immune aging, increasing susceptibility to infection or chronic inflammation. TRIM’s full impact on age-related and neuroinflammatory processes remains an area for continued research.

Long-term or repeated exposure to foreign plasma proteins may sensitize the immune system, creating antibodies against transfused proteins or cells, which complicates future transfusions. Chronic transfusions could potentially provoke autoimmunity, unwanted inflammation, or, as observed in other transfusion settings, increase the risk of adverse cardiovascular events. Additionally, while passive immunity is useful in treating infectious diseases, transferring plasma for non-therapeutic purposes carries potential risks that remain incompletely understood.

#### Plasma and component therapy for aging research: caution in clinical translation

Emerging preclinical research into plasma and blood component therapies for age-related rejuvenation has garnered significant interest, particularly for modulating factors that impact the vasculature and brain. However, while preclinical models, such as heterochronic plasma transfer in animals, indicate potential rejuvenative effects, translating these findings to clinical applications is complex and fraught with challenges.

While preclinical studies indicate plasma therapies can hold promise for age-related conditions, it is critical to underscore that current findings are far from sufficient to endorse clinical anti-aging plasma therapies. Existing research supports plasma therapies primarily in traditional clinical contexts, such as treating coagulation disorders or providing immune support, but applying these techniques to anti-aging is speculative at this stage. Future development must involve rigorous clinical trials to test efficacy and safety, with a focus on targeted component therapy to maximize therapeutic benefit and minimize adverse reactions.

The primary focus in translational anti-aging research has been on selective component therapy rather than whole plasma transfusions. Therapies focused on plasma fractions present some practical advantages, such as minimizing the volume administered, reducing allergic response risks, and lowering the potential for transfusion-transmitted infections through virus inactivation protocols. The advantages of component therapy also include enhanced compatibility, standardization, and reproducibility. This approach holds promise as it allows for targeted delivery of specific rejuvenative molecules while reducing exposure to unnecessary plasma components that may provoke unwanted immune reactions. Nevertheless, whole plasma and component transfusions both present immune-related risks. These complications underscore the need for controlled administration and patient monitoring, as well as virus inactivation and stringent compatibility testing. The aforementioned features make component therapy a viable pathway for research, but strict safety protocols and extensive validation are critical before any clinical application.

In summary, while plasma and blood component therapies offer a compelling research direction for addressing age-related degeneration, their clinical translation for anti-aging remains premature and should not be pursued outside well-controlled research settings.

## Future directions

The field of aging and rejuvenation research is rapidly expanding, with an increasing focus on understanding how circulating factors, cellular interactions, and systemic pathways impact age-related decline across different organ systems. Groundbreaking studies in heterochronic parabiosis, heterochronic blood and plasma exchange, and the identification of rejuvenating and progeronic factors have provided strong evidence that the aging process is influenced not only by intrinsic cellular mechanisms but also by systemic signals that modulate vascular and CNS health. Future research directions should emphasize the identification and characterization of these factors and their pathways, with a particular focus on how they impact vascular integrity, neuroprotection, cognitive function, and resilience against age-related diseases. Population-based cohort studies, such as the Whitehall II, Health ABC Study, Cardiovascular Health Study and UK Biobank, have revealed that systemic biomarkers—including GDF15 and inflammatory mediators—strongly correlate with frailty, cognitive decline, and cerebrovascular pathology [64, 1282, 1772]. Integrating such data with experimental findings can help bridge model-to-human translation and guide precision strategies for vascular aging and dementia prevention.

One of the most pressing goals is to identify the specific factors within young blood or plasma that contribute to rejuvenation in aged tissues, particularly in the vasculature and CNS. While factors like GDF11, IGF- 1, and specific microRNAs have shown promise, the precise combination of these elements that produce the most significant anti-aging effects remains to be determined. Advanced omics technologies, such as proteomics, metabolomics, and transcriptomics, are essential for uncovering the full profile of these rejuvenating agents, as well as for determining how these elements might be optimized for therapeutic interventions. Recent advances in single-cell proteomics, metabolomics, and DNA methylation-based epigenetic clocks offer powerful tools to decode the systemic aging process in humans. These technologies hold the potential to identify circulating signatures of cerebrovascular aging, monitor therapeutic efficacy, and stratify patients based on biological rather than chronological age. When applied longitudinally in large cohorts, they could further illuminate the systemic pathways contributing to cognitive decline and dementia.

Understanding the cellular and molecular mechanisms through which these circulating factors exert their effects is critical for translating these findings into effective therapies. Many identified factors, such as NAD^+^, IGF- 1, and exosomes containing regulatory RNAs, signal through pathways that influence endothelial function, blood–brain barrier integrity, inflammation, and neuroprotection. Elucidating these mechanisms is particularly important for uncovering how these factors may protect against vascular aging, promote cerebral blood flow, and enhance cognitive resilience in the aging CNS. Different tissues, including the vascular endothelium and CNS, may exhibit unique responses to circulating factors, underscoring the importance of understanding tissue-specific effects.

Aging is a complex, multifactorial process that impacts multiple organ systems. Combining approaches that target different aspects of aging, such as enhancing vascular health, promoting neurogenesis, reducing neuroinflammation, and supporting cellular stress resilience, may offer synergistic effects. For example, integrating therapies that restore NAD^+^ levels with those that support blood–brain barrier integrity could amplify the protective effects on the CNS. Research into combination therapies that incorporate rejuvenating factors identified through parabiosis, blood or plasma exchange experiments, anti-inflammatory agents, and senolytics holds promise for extending healthspan, mitigating age-related decline and reducing the incidence of age-related diseases. While the identification of rejuvenating factors and the development of therapies hold significant potential, their safety and efficacy over long periods remain critical considerations. Certain circulating factors, if misregulated, may pose risks, such as promoting tumorigenesis or impairing immune function. It will be essential to understand dose–response relationships, timing, and potential side effects, particularly in older individuals with compromised immune systems. Establishing rigorous safety protocols and conducting extensive preclinical and clinical trials will be key for translating these discoveries into clinical applications.

To answer these questions, novel methodologies and experimental models are essential. Single-cell RNA sequencing and high-resolution mass spectrometry allow researchers to investigate the effects of rejuvenating factors on a cellular level, identifying specific changes in gene expression, metabolite profiles, and protein interactions. These tools are particularly valuable for studying heterochronic parabiosis and other models of blood exchange, where identifying differential effects in young and old animals can illuminate pathways involved in rejuvenation. Machine learning algorithms and AI-based predictive models are also becoming integral to this research. By analyzing large datasets from omics studies, researchers can predict candidate rejuvenating factors and potential side effects, accelerating the translation of these findings into therapeutic applications. Additionally, CRISPR-based gene editing may allow scientists to test the functional importance of individual factors and pathways implicated in rejuvenation.

The discovery of rejuvenating factors and the exploration of their systemic impacts provide a foundation for future therapeutic interventions. Translating these findings from laboratory models to clinical applications will require scalable, minimally invasive treatments that are accessible to the aging population. Small molecules, biologics, or engineered nanoparticles designed to mimic the effects of rejuvenating factors offer promising avenues for clinical applications, provided their safety and efficacy can be established through rigorous clinical testing. Plasma dilution, as a practical alternative to plasma exchange, may also hold potential for reducing age-associated pro-inflammatory factors in a controlled manner.

## Conclusion

The studies of aging and rejuvenation have revealed that the aging process is not solely an outcome of intrinsic cellular changes but is also profoundly shaped by systemic influences, including circulating factors that impact the vascular and central nervous systems. This insight opens up new ways to explore therapeutic interventions that can promote vascular and neurological health, potentially slowing or even reversing aspects of age-related decline. The identification of specific factors within young blood, along with advancements in our understanding of how these factors affect tissues, has opened new possibilities for addressing age-related deterioration. Research into circulating molecules, such as growth differentiation factors, microRNAs, and exosomes, has shown that these elements can support endothelial integrity, cognitive resilience, and cellular repair mechanisms. Importantly, this work underscores the value of multifaceted approaches that may combine blood-based rejuvenative therapies with strategies aimed at mitigating inflammation and promoting cellular resilience. The ultimate goal in this field is to translate these discoveries into safe, accessible therapies that can enhance quality of life and health span in aging populations. By refining our understanding of the mechanisms underlying these rejuvenating effects and carefully assessing their therapeutic potential, the field is moving toward interventions that could substantially reduce the burden of age-related diseases and improve overall well-being in later life.
